# Cellular senescence mediates retinal ganglion cell survival regulation post‐optic nerve crush injury

**DOI:** 10.1111/cpr.13719

**Published:** 2024-07-18

**Authors:** Yao Yao, Xin Bin, Yanxuan Xu, Shaowan Chen, Si Chen, Xiang‐Ling Yuan, Yingjie Cao, Tsz Kin Ng

**Affiliations:** ^1^ Joint Shantou International Eye Center of Shantou University and The Chinese University of Hong Kong Shantou China; ^2^ Department of Ophthalmology and Visual Sciences The Chinese University of Hong Kong Hong Kong China

## Abstract

Traumatic optic neuropathy refers to optic nerve (ON) injury by trauma, including explosion and traffic accident. Retinal ganglion cell (RGC) death is the critical pathological cause of irreversible visual impairment and blindness in ON injury. We previously investigated the patterns of 11 modes of cell death in mouse retina post‐ON injury. Here we aimed to identify additional signalling pathways regulating RGC survival in rodents post‐ON injury. RNA sequencing analysis identified the upregulation of inflammation and cellular senescence‐related genes in retina post‐ON injury, which were confirmed by immunoblotting and immunofluorescence analyses. Increased expression of senescence‐associated β‐galactosidase (SA‐βgal) in RGCs and activation of microglia were also found. Transforming growth factor‐β receptor type II inhibitor (LY2109761) treatment suppressed p15^Ink4b^ and p21^Cip1^ protein and SA‐βgal expression and promoted RGC survival post‐ON injury with decreasing the expression of cell death markers in retina. Consistently, senolytics (dasatinib and quercetin) treatments can promote RGC survival and alleviate the reduction of ganglion cell complex thickness and pattern electroretinography activity post‐ON injury with reducing SA‐βgal, p15^Ink4b^, p21^Cip1^, microglial activation and cell death marker expression. In summary, this study revealed the activation of cellular senescence in rodent retina post‐ON injury and contribute to RGC survival regulation. Targeting cellular senescence can promote RGC survival after ON injury, suggesting a potential treatment strategy for traumatic optic neuropathy.

## Graphical abstract

This study revealed the activation of cellular senescence in rodent retina post‐optic nerve injury and contribute to retinal ganglion cell survival regulation. Senolytics and TGF‐β receptor inhibitor can be the potential treatment strategies promoting retinal ganglion cell survival after optic nerve injury by targeting cellular senescence.

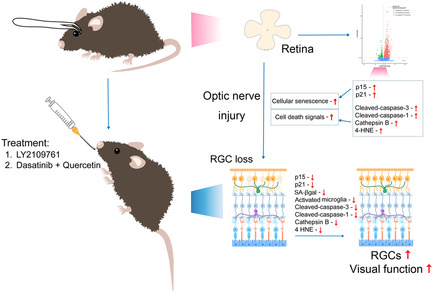

## 1 INTRODUCTION

Traumatic optic neuropathy refers to an ocular condition with direct or indirect acute injury to the optic nerve (ON) by trauma, resulting in irreversible visual impairment or even blindness.^1^ Traumatic optic neuropathy can be resulted from explosion, traffic accidents, sports and recreational activities.^2^, ^3^ The World Health Organization has estimated that trauma has led to 1.6 million binocular blindness, 2.3 million people with both eyes low vision, and 19 million monocular blindness worldwide.^4^ Direct ON injury includes penetrating trauma and impinging injury by bone fragments or orbital haemorrhage, whereas indirect injury commonly occurs during head or globe injury when the ON is sheared and avulsed by the rotational forces.^5^ Progressive retinal ganglion cell (RGC) loss and axonal degeneration are the major causes of vision loss in ON injury.^6^ There is still no effective clinical treatment rescuing RGC death in ON injury. Exploring new treatments to promote RGC survival post‐ON injury is warranted.

As the neurons in the central nervous system, the intrinsic self‐repair ability of RGCs is very limited. Besides, the lack of appropriate trophic support and aberrant cell death also contribute to the RGC loss post‐ON injury.^7^, ^8^ Our recent study has revealed the activation of apoptosis, autolysis, pyroptosis and ferroptosis as the major modes of cell death in RGCs post‐ON injury.^9^ Moreover, our previous investigations on RGC survival promotion treatments with the administration of stem cells, green tea extract, and growth hormone‐releasing hormone analogs demonstrated the involvement of inflammation, oxidative stress, and survival signalling pathways in RGC survival regulation post‐ON injury,^10^, ^11^, ^12^, ^13^, ^14^, ^15^ indicating that multiple signalling pathways could be involved in the regulation of RGC survival post‐ON injury. However, the mechanisms regulating RGC death post‐ON injury are still not completely elucidated. Here we aimed to identify additional signalling pathways involved in the regulation of RGC survival in rodents post‐ON injury. Retinal transcriptomes post‐optic injury was determined by RNA sequencing analysis. Expression analyses and intervention treatments were applied to confirm the involvement of the identified pathway in RGC survival regulation post‐ON injury.

## 2 MATERIALS AND METHODS

### 2.1 Animals

Young adult male Fischer F344 rats (8–10 weeks, 150–200 g) and male C57BL/6 mice (6–8 weeks, 20–25 g) were purchased from Beijing Vital River Laboratory Animal Technology Co. Ltd. (Beijing, China) and maintained in specific pathogen‐free grade animal facility at 22 ± 1°C and 40 ± 10% humidity with 12‐h dark/light cycle. Standard rodent chow and water were provided ad libitum. The experimental protocols have been approved by the Animal Experimentation Ethics Committee of Joint Shantou International Eye Center of Shantou University and the Chinese University of Hong Kong (approval number: EC20191119(5)‐P01). All animal experiments followed the guidelines of the Association for Research in Vision and Ophthalmology Statement on Use of Animals in Ophthalmic and Vision Research and the Animal Research: Reporting of In Vivo Experiments (ARRIVE) guidelines. Three rats were used in each group for the RNA sequencing analysis, and five mice were used in each group for other experiments. A schematic diagram of the experimental design was presented in Supplementary Figure 1.

### 2.2 Optic nerve injury

Unilateral ON injury was induced in rats and mice by the ON crush surgery according to our previously established protocol.^9^, ^14^ Briefly, the rodents were first anaesthetised with intramuscular injection of a mixture (0.1 mL/100 g) of 70 mg/mL Zoletil® (Virbac, Carros, France) and 20 mg/mL xylazine (Sigma‐Aldrich, St. Louis, MO). A horizontal incision was made at the fornical conjunctiva of the right eye. After separating the adipose tissue and extraocular muscles, the ON was exposed and crushed at 1–1.5 mm behind the eyeball for 5 s without damaging the ophthalmic artery using an angled jeweller's forceps (Dumont #5; Roboz, Rockville, MD) under an operating microscope. Effective ON injury was confirmed by the presence of a clear gap across the entire ON at the crush site.

### 2.3 RNA sequencing analysis

Adhered to our previous study,^14^ the rats, at Day 7 post‐ON injury, were sacrificed, and the retina was dissected. Total RNA of the retina was extracted and purified with the TRIzol reagent (Thermo Fisher Scientific, Waltham, MA) according to the manufacturer's protocols. RNA sequencing experiments were conducted by the Novogene Co. Ltd. (Beijing, China). Briefly, the RNA integrity was evaluated by the Agilent Bioanalyzer 2100 (Agilent Technologies, Santa Clara, CA) using the RNA Nano 6000 Assay Kit (Agilent Technologies). The mRNA was isolated from total RNA using oligo dT magnetic beads, and the complementary DNA was synthesized by the M‐MuL V Reverse Transcriptase and DNA Polymerase I. With the adenylation at the 3′ ends of the DNA fragments, the adaptor with the hairpin loop structure was ligated for hybridization. Polymerase chain reaction (PCR) was performed on the 370–420 base pairs (bp) cDNA fragments purified by the AMPure XP kit (Beckman Coulter Life Sciences, Indianapolis, IN) with the Phusion High‐Fidelity DNA polymerase, universal PCR primers and index (X) primer. The PCR products were purified, and the library quality was assessed by the Agilent Bioanalyzer 2100. The library preparations were sequenced using the Illumina Novaseq 6000 platform to generate 150‐bp paired‐end reads. After removing the low‐quality reads and reads containing the adapter or poly‐N from raw data, the paired‐end clean reads were aligned to the reference genome using Hisat2 v2.0.5. FeatureCounts v1.5.0‐p3 was used to count the number of reads mapped to each gene, and the fragments per kilobase of transcript sequence per millions base pairs sequenced (FPKM) of each gene was calculated. Principle component analysis (PCA) based on the whole transcriptome profiles was adopted to confirm the distinct clustering of different groups. The volcano plot was used to present the gene expression changes in rat retina with ON injury compared to that of the normal rats without ON injury. Differential gene expression analysis was performed using the DESeq2 R package (1.20.0). *P* values were adjusted using the Benjamini & Hochberg method to control the false discovery rate. Differential gene expression was considered as log_2_ fold change ≥1 and corrected *P* (*P*_corr_) <0.05. Hierarchical clustering analysis of differentially expressed genes was also used to visualize the distinct gene expression patterns in different groups.

Gene ontology analysis of the differential expressed genes was evaluated by DAVID Bioinformatics Resources 6.8.^16^ Enrichment score >1.3 was considered as statistically significant. Based on the gene ontology analysis and the fold changes, 22 differentially expressed genes were selected for further SYBR green PCR validation in mice at Day 0–14 post‐ON injury. Total RNA of the mouse retina was converted to complementary DNA using the SuperScript III reverse transcriptase and amplified by SYBR Green I Master Mix (TaKaRa Bio Inc., Shiga, Japan) in the LightCycler 480 system (Roche, Basel, Switzerland) with respective specific primers (Supplementary Table 1). *Actb* was used as the housekeeping gene for normalization. Relative expression was calculated by the 2^−ΔΔCt^ method as compared to the normal mice without ON injury.

### 2.4 Immunoblotting analysis

The mice, at Day 0–14 post‐ON injury, were sacrificed, and the dissected retina was lysed in cold radio‐immunoprecipitation assay (RIPA) buffer (Sigma‐Aldrich) supplemented with protease and phosphatase inhibitors (Roche). Total protein concentrations of the cell lysates were measured by the Micro BCA Protein Assay Kit (Thermo Fisher Scientific). After 95°C denaturation for 5 min, equal amounts of total proteins (20 μg) were resolved in 10% SDS‐polyacrylamide gels and electro‐transferred to nitrocellulose membranes for sequential probing with primary antibody against p15^Ink4b^ (catalogue number: ab53034; Abcam, Cambridge, UK) or p21^Cip1^ (catalogue number: ab188224; Abcam), and respective secondary antibody conjugated with horseradish peroxidase (Santa Cruz Biotechnology, Dallas, TX). The signal intensities were detected by enhanced chemiluminescence (Thermo Fisher Scientific) with the ChemiDocTM XRS^+^ system (Bio‐Rad Laboratories, Hercules, CA). β‐actin was used as the housekeeping protein for normalization.

### 2.5 LY2109761 treatment

LY2109761 (MedChem Express, Monmouth Junction, NJ), an inhibitor of TGF‐β receptor, was first solved in dimethyl sulfoxide (DMSO) and diluted before administration with saline containing 10% sulfobutylether‐β‐cyclodextrin (MedChem Express) to a working concentration of 1 mg/mL with the concentration of DMSO below 5%. The mice, after ON injury, were fed intragastrically with LY2109761 (0.3 mg/25 g)^17^ every day for 14 days. The mice in the vehicle control group was fed with equal volume of the saline‐diluted solvents every day for 14 days. The treated mice were sacrificed at Day 5 and 14 post‐ON injury for further analyses.

### 2.6 Retinal ganglion cell survival analysis

The numbers of RGCs in the retina were evaluated by the immunofluorescence analysis according to our previous protocol.^18^ Briefly, the mice were sacrificed at Day 14 post‐ON injury and perfused with 4% paraformaldehyde (Sigma‐Aldrich). The eyeballs were enucleated, and the retinas were dissected and further fixed with 4% paraformaldehyde for 2 h. After fixation, the retinas were blocked and permeabilized with 5% normal goat serum (NGS) and 0.3% Triton X‐100 in phosphate buffered saline (PBS) at room temperature for 1 h, and probed with the anti‐neuron‐specific βIII‐tubulin antibody (catalogue number: 5568; Cell Signalling Technology, Danvers, MA) at 4°C overnight, followed by the incubation of Alexa Fluor‐555‐conjugated secondary antibody at room temperature for 2 h. The stained retinas were mounted with anti‐fading mounting medium and imaged in eight fields for each retina (each field: 0.775 × 0.775 mm^2^) under a confocal microscope (Leica TCS SP5 II, Leica Microsystems, Wetzlar, Germany). The numbers of the positively stained cells were counted manually using the Point Tool in the ImageJ software (version 1.47; National Institutes of Health, Bethesda, MD), and the average density of the positively stained cells was calculated.

### 2.7 Immunofluorescence analysis

The mice were sacrificed at Day 5 or 14 post‐ON injury and perfused with 4% paraformaldehyde. The eyeballs were enucleated for post‐fixation in 4% paraformaldehyde at 4°C for overnight. The fixed eyeballs were cryo‐protected with 10%–30% sucrose gradient and cryo‐embedded in optimal cutting temperature compound (Leica). The cryo‐sections (10 μm) with pupil‐ON position were blocked and permeabilized with 5% NGS and 0.3% Triton X‐100 in PBS at room temperature for 1 h, and probed with the primary antibodies against p15^Ink4b^, p21^Cip1^, cleaved caspase‐3 (catalogue number: 9661; Cell Signalling Technology), cleaved cathepsin B (catalogue number: ab214428; Abcam), cleaved caspase‐1 (catalogue number: ab179515; Abcam), and 4‐hydroxynonenal (4‐HNE; catalogue number: ab46545, Abcam) at 4°C for overnight, followed by the incubation with respective secondary antibodies conjugated with Alexa Fluor‐488 at room temperature for 2 h. The stained sections were mounted with the anti‐fading mounting medium, and the fluorescence signals were visualized by a confocal microscopy (Leica TCS SP5 II). For each group, 20 retinal images at 200 μm from the ON were analysed.

### 2.8 Senescence‐associated β‐galactosidase staining

The senescence‐associated β‐galactosidase (SA‐βgal) staining were performed using the Senescence β‐Galactosidase Staining Kit (Cell Signalling Technology). Briefly, the mice were sacrificed at Day 5 post‐ON injury and perfused with 4% paraformaldehyde. The eyeballs were enucleated for post‐fixation in 4% paraformaldehyde at 4°C for overnight. The fixed eyeballs were cryoprotected with 10%–30% sucrose gradient and cryo‐embedded in optimal cutting temperature compound. The cryo‐sections (10 μm) with pupil‐ON position were blocked with the Fixative Solution at room temperature for 20 min, and, after PBS washing, incubated with the β‐Galactosidase Staining Solution (pH 6.0) at 37°C for 48 h.^19^ The stained sections were mounted with 70% glycerol, and the blue signals were visualized under a light microscopy (Leica DM4B). For each group, 20 retinal images at 200 μm from the ON were analysed.

### 2.9 Treatment with senolytics

Dasatinib (Sigma‐Aldrich) and quercetin (Sigma‐Aldrich) were used as senolytics according to a previous study.^20^ Dasatinib was first solved in DMSO, whereas quercetin was dissolved in polyethylene glycol 300 (PEG300). Dasatinib and quercetin diluted before administration with saline (1% DMSO and 10% PEG300, respectively). The mice, after ON injury, were fed intragastrically with dasatinib (5 mg/kg) and/or quercetin (50 mg/kg) every day for 14 days. The mice in the vehicle control group was fed with equal volume of the saline with 1% DMSO and 10% PEG300 every day for 14 days. The treated mice were sacrificed at Day 5 and 14 post‐ON injury for further analyses.

### 2.10 Ganglion cell complex analysis

Cross‐sectional retina of the mice was imaged by the spectral domain‐optical coherence tomography (OCT) mode of the RETI*map*® machine (Roland, Germany) in vivo at baseline and at Day 4, 7 and 14 post‐ON injury. Briefly, the mice were anaesthetised with intramuscular injection of a mixture (0.1 mL/100 g) of 70 mg/mL Zoletil® (Virbac) and 20 mg/mL xylazine (Sigma‐Aldrich). Retinal images were taken with the mice given a 100‐diopter corneal contact lens. Two cross‐sectional images were taken across the ON head (as reference point) for each eye of the mouse. The thickness of the ganglion cell complex (GCC), which is composed of retinal nerve fibre layer, ganglion cell layer, and inner plexiform layer, was measured by ImageJ software at 300 μm from the optic papilla at the nasal, temporal, superior and inferior positions.

### 2.11 Pattern electroretinography analysis

The function of RGCs was evaluated by the pattern electroretinography (ERG) analysis using the RETI*map*® machine (Roland, Germany) in vivo at baseline and at Day 4, 7 and 14 post‐ON injury, adhered to the protocol of the International Society for Clinical Electrophysiology of Vision. Briefly, the mice were anaesthetised with intramuscular injection of a mixture (0.1 mL/100 g) of 70 mg/mL Zoletil® (Virbac) and 20 mg/mL xylazine (Sigma‐Aldrich). The mice were placed on a heating pad to maintain the body temperature at 37°C. Tropicamide eye drop was applied before recording to dilate the pupil. The reference electrode was placed under the skin between the eye and the ear of the same side, and the ground electrode was placed at the root of the tail. The corneal electrode was placed on the cornea. Before recording, the impedance between the reference and ground electrodes was maintained below 10 kΩ, with a preference of <5 kΩ to enhance the signal quality. With each trace recording up to 600 ms, two consecutive recordings of 200 traces were averaged. The visual stimuli were presented in a checkerboard pattern with an alternating black and white striped grating, covering 2° full field with a centre contrast of 99% and a reversal frequency of 1 Hz. The reversal rate of the stimuli was set at 4.0 ± 0.8 reversals per second to ensure adequate temporal resolution for capturing potential changes. For the recording, an amplifier was applied with a range of ±20 μV within the frequencies of 5–30 Hz. The first positive peak in the waveform was designated as P1 and the second negative peak as N2. P1 was typically around 45–60 ms, whereas N2 was generally around 90–100 ms. The amplitude was measured from P1 to N2.

### 2.12 Microglia analysis

The mice were sacrificed at Day 7 post‐ON injury and perfused with 4% paraformaldehyde. The eyeballs were enucleated, and the retinas were dissected for further 2‐h post‐fixation. The retinas were blocked and permeabilized with 5% NGS and 0.3% Triton X‐100 in PBS for 1 h, and probed with the anti‐Iba‐1 antibody (FUJIFILM Wako Pure Chemical Corporation, Tokyo, Japan) at 4°C overnight, followed by the incubation with the secondary antibody conjugated with Alexa Fluor‐555 at room temperature for 2 h. The stained retinas were mounted on slides with anti‐fading mounting medium and imaged in 8 fields for each retina (each field: 0.775 × 0.775 mm^2^) using a confocal microscope (Leica TCS SP5 II). The numbers of the resting and activated microglia were counted manually using the Point Tool in the ImageJ software (version 1.47). Resting microglia are ramified in shape, and the activated microglia acquire an amoeboid cell shape.

### 2.13 Statistical analysis

Data were presented as the mean of the results from 3 rats or 5 mice ± standard deviation (SD), and compared by the independent T‐test or one‐way analysis of variance (ANOVA) with post hoc Tukey test. All statistical analyses were conducted by IBM SPSS Statistics 23 (SPSS Inc., Chicago, IL). *P* < 0.05 was considered as statistically significance.

## 3 RESULTS

### 3.1 Post‐optic nerve injury retinal transcriptome analysis and validation

**FIGURE 1:**
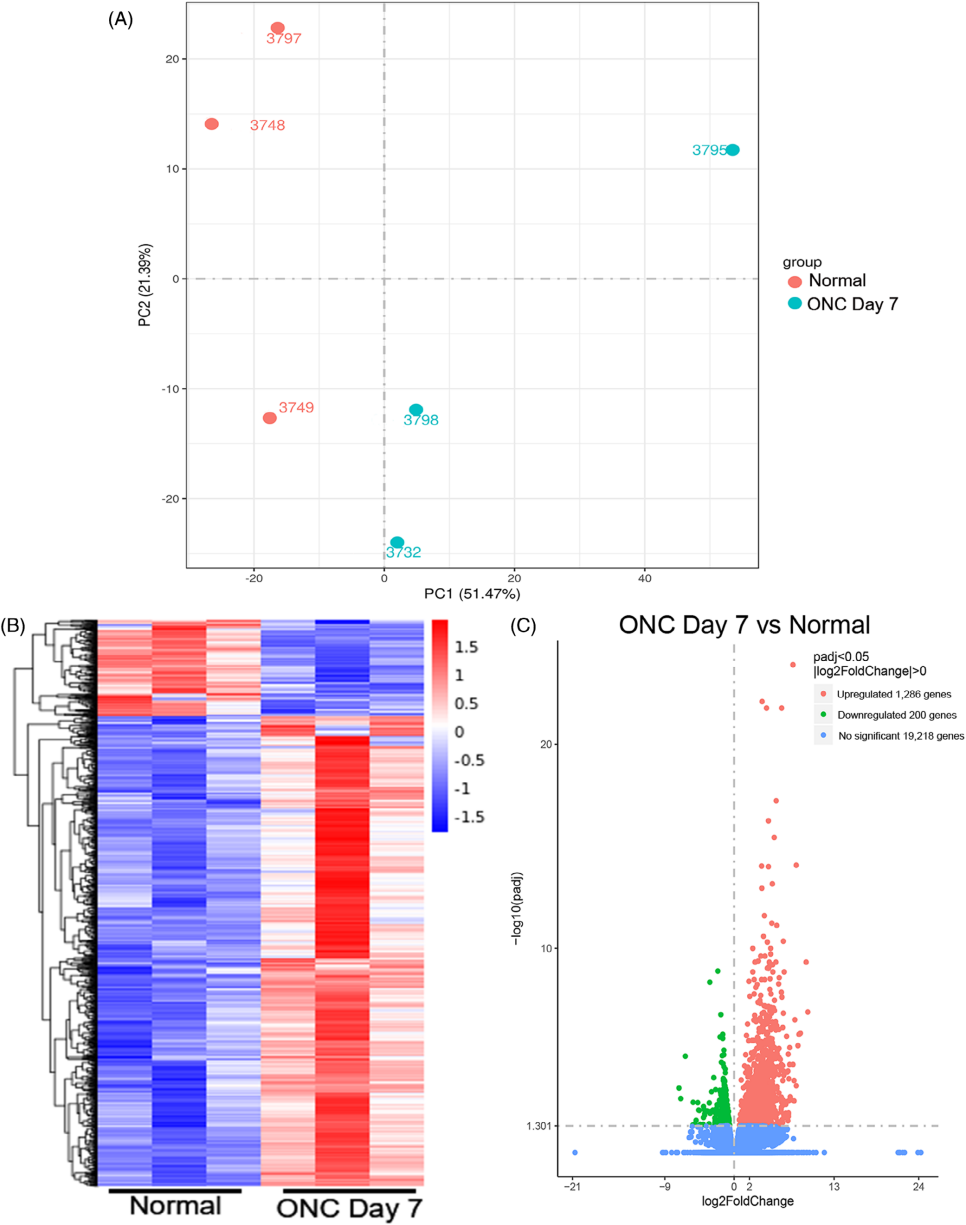
RNA sequencing analysis on rat retina after optic nerve injury. (A) Principle component analysis on the whole transcriptome profiles of the rat retinas at Day 7 post‐optic nerve (ON) injury and compared to that of normal rats without ON injury. (B) Hierarchical clustering analysis of differentially expressed genes in rat retina with and without ON injury. (C) Volcano plots of gene expression changes in rat retina with ON injury compared to that of the normal rats without ON injury by the independent *T*‐test. Red dots: Significantly upregulated genes; Green dots: Significantly downregulated genes; Blue dots: No significant changes. Significant differential expression was defined as log_2_ fold change ≥1 and corrected *P* < 0.05.

Our previous study showed around 50% of RGC loss at Day 7 post‐ON injury.^9^ Adhered to our previous study,^14^ RNA sequencing analysis on the rat retina at Day 7 post‐ON injury demonstrated that PCA (Figure 1A) and hierarchical clustering analysis (Figure 1B) showed differentially clustered expression profiles between the retinas of the rats with ON injury and the retinas of the normal rats without ON injury. The volcano plot analysis revealed 1486 differentially expressed genes among total 20,704 genes identified in the rat retina with ON injury as compared to that in the normal rats (Figure 1C), with 1286 significantly upregulated genes (Supplementary Table 2) and 200 significantly downregulated genes (Supplementary Table 3). Functional annotation clustering in gene ontology analysis showed that the differentially expressed genes were involved in the innate immune response (enrichment score = 20.43, *P* = 4.38 × 10^−12^), complement pathway (enrichment score = 4.69, *P* = 2.00 × 10^−6^), NAD^+^ nucleosidase activity (enrichment score = 4.05, *P* = 3.42 × 10^−8^), vision (enrichment score = 2.90, *P* = 2.78 × 10^−5^), NLRP3 inflammasome complex (enrichment score = 2.74, *P* = 1.65 × 10^−4^), cellular senescence (enrichment score = 1.78, *P* = 0.033), and execution phase of apoptosis (enrichment score = 1.54, *P* = 0.011) (Table 1). The RNA sequencing analysis confirmed the involvement of apoptosis and pyroptosis in ON injury. Critically, additional potential pathways are identified and could also be involved in the regulation of RGC survival post‐ON injury.

**TABLE 1:** Gene ontology analysis on differentially expressed genes in rat retina post‐optic nerve injury.

Functional annotation clusters	Enrichment score	Gene count	%	*P*
Innate immune response	20.43	73	4.95	4.38 × 10^−12^
Complement pathway	4.69	14	0.95	2.00 × 10^−6^
NAD^+^ nucleosidase activity	4.05	14	0.95	3.42 × 10^−8^
Growth factor binding	3.59	10	0.68	0.002
Th17 cell differentiation	2.93	19	1.29	0.002
Vision	2.90	12	0.81	2.78 × 10^−5^
NLRP3 inflammasome complex	2.74	6	0.41	1.65 × 10^−4^
Signal transduction inhibitor	2.62	10	0.68	9.45 × 10^−4^
Integrin complex	2.36	9	0.61	2.81 × 10^−4^
MyD88‐dependent toll‐like receptor signalling pathway	2.28	7	0.47	5.20 × 10^−4^
Cellular glucuronidation	2.27	7	0.47	2.20 × 10^−4^
Cell activation	2.25	6	0.41	0.003
EGFR tyrosine kinase inhibitor resistance	2.18	15	1.02	0.006
MHC class II protein complex binding	1.99	7	0.47	2.65 × 10^−4^
Protein tyrosine phosphatase activity	1.91	17	1.15	0.003
Actin filament depolymerization	1.90	5	0.34	0.022
Developmental protein	1.87	56	3.80	0.014
Protein phosphorylation	1.85	59	4.00	8.55 × 10^−4^
Heterotrimeric G‐protein complex	1.79	9	0.61	0.002
Cellular senescence	1.78	24	1.63	0.033
NADPH oxidase complex	1.67	4	0.27	0.047
Apelin signalling pathway	1.66	25	1.69	3.43 × 10^−4^
Lipoteichoic acid binding	1.63	4	0.27	0.007
Cell projection assembly	1.60	8	0.54	0.002
Phosphatidylinositol‐mediated signalling	1.60	11	0.75	0.002
Sphingosine‐1‐phosphate signalling pathway	1.57	5	0.34	0.014
Execution phase of apoptosis	1.54	6	0.41	0.011
Laminin complex	1.48	4	0.27	0.015
Phosphatidylinositol 3‐kinase complex	1.48	7	0.47	0.005
Leukotriene biosynthesis	1.43	4	0.27	0.011
Nucleoside transport	1.33	3	0.20	0.014

**FIGURE 2:**
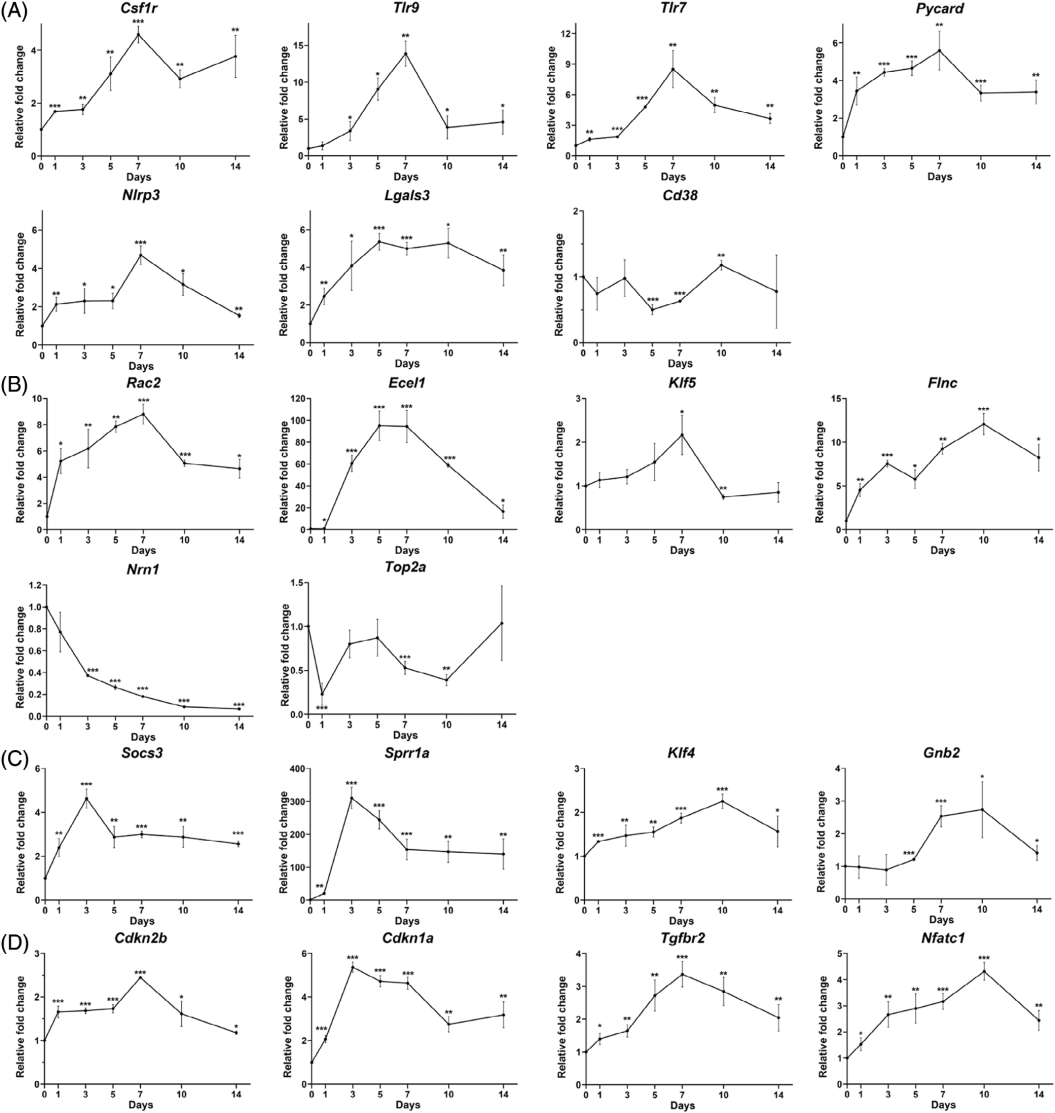
Gene expression analysis on mouse retina along the time course of optic nerve injury. SYBR green polymerase chain reaction on the expression of genes related to (A) inflammation (*Csf1r*, *Tlr9*, *Tlr7*, *Pycard*, *Lgals3*, *Nlrp3* and *Cd38*), (B) neuroprotection (*Rac2*, *Ecel1*, *Klf5*, *Flnc*, *Nrn1* and *Top2a*), (C) axonal regeneration (*Socs3*, *Sprr1a*, *Klf4* and *Gnb2*), and (D) cellular senescence (*Cdkn2b*, *Cdkn1a*, *Tgfbr2* and *Nfatc1*) in mouse retina at Day 1, 3, 5, 7, 10 and 14 post‐optic nerve (ON) injury, comparing to that before ON injury (Day 0). *Actb* was used as housekeeping gene for normalization. Data presented as mean of relative fold change (2^−ΔΔCt^) ± standard deviation and compared by the independent *T*‐test. *P < 0.05; **P < 0.01; ***P < 0.001.

To validate the results of RNA sequencing analysis (Table 2), 21 differentially expressed genes related to inflammation (*Csf1r*, *Tlr9*, *Tlr7*, *Pycard*, *Lgals3*, *Nlrp3* and *Cd38*), neuroprotection (*Rac2*, *Ecel1*, *Klf5*, *Flnc*, *Nrn1* and *Top2a*), axonal regeneration (*Socs3*, *Sprr1a*, *Klf4* and *Gnb2*), and cellular senescence (*Cdkn2b*, *Cdkn1a*, *Tgfbr2* and *Nfatc1*) were selected for SYBR Green PCR analysis based on their expression and biological functions in mouse retina according to different time course of ON injury in our previous study.^9^ For the inflammation‐related genes, the increased expression of *Csf1r*, *Tlr9*, *Tlr7*, *Pycard* and *Nlrp3* genes in mouse retina reached a peak at Day 7 post‐ON injury by 4.59 ± 0.32 folds (*P* < 0.001), 13.88 ± 1.69 folds (*P* = 0.006), 8.51 ± 1.83 folds (*P* = 0.002), 5.59 ± 1.03 folds (*P* = 0.001), and 4.69 ± 0.49 folds (*P* < 0.001) respectively as compared to the retina before ON injury (Day 0) (Figure 2A). *Lgals3* gene also showed highest increased expression from Day 5 by 5.36 ± 0.44 folds (*P* < 0.001) to Day 10 by 5.29 ± 0.79 folds (*P* = 0.011). Yet, *Cd38* gene did not follow the trend of expression as the RNA sequencing analysis.

**TABLE 2:** Fold change and significance of RNA sequencing analysis on retina with optic nerve injury.

Gene	Log_2_ fold change	*P*_corr_
*Csf1r*	+3.63	1.08 × 10^−6^
*Tlr9*	+6.08	8.94 × 10^−5^
*Tlr7*	+4.55	3.93 × 10^−7^
*Pycard*	+4.04	2.69 × 10^−4^
*Nlrp3*	+4.08	7.57 × 10^−5^
*Lgals3*	+6.42	1.49 × 10^−7^
*Cd38*	+8.31	5.78 × 10^−6^
*Rac2*	+5.45	4.70 × 10^−10^
*Ecel1*	+8.08	8.41 × 10^−15^
*Klf5*	+7.21	4.95 × 10^−5^
*Flnc*	+7.66	1.29 × 10^−24^
*Nrn1*	−2.75	0.008
*Top2a*	+7.04	1.50 × 10^−5^
*Socs3*	+5.11	7.97 × 10^−8^
*Sprr1a*	+9.36	4.70 × 10^−10^
*Klf4*	+2.00	0.049
*Gnb2*	+1.61	0.011
*Cdkn2b*	+5.03	0.001
*Cdkn1a*	+1.87	0.002
*Tgfbr2*	+3.33	0.004
*Nfatc1*	+3.21	0.007

*Note*: ‘*P*_corr_’ corrected *P*.

For the genes related to neuroprotection, the increased expression of *Rac2* and *Klf5* genes in mouse retina reached a peak at Day 7 post‐ON injury by 8.80 ± 0.76 folds (*P* < 0.001) and 2.17 ± 0.45 folds (*P* = 0.021) respectively as compared the retina before ON injury (Figure 2B). Moreover, *Ecel1* and *Flnc* genes were also significantly upregulated to a peak at Day 5 post‐ON injury by 94.99 ± 13.50 folds (*P* < 0.001) and at Day 10 by 12.06 ± 1.23 folds (*P* < 0.001) respectively as compared the retina before ON injury. In contrast, the expression of *Nrn1* gene was significantly downregulated from Day 3 post‐ON injury by 62.7% (*P* < 0.001) to Day 14 by 93.2% (*P* < 0.001) as compared the retina before ON injury, whereas *Top2a* gene did not follow the trend of expression as the RNA sequencing analysis.

For the genes related to axonal regeneration, the increased expression of *Socs3* and *Sprr1a* genes in mouse retina reached a peak at Day 3 post‐ON injury by 4.64 ± 0.44 folds (*P* < 0.001) and 310.32 ± 32.76 folds (*P* < 0.001) respectively as compared the retina before ON injury (Figure 2C). In addition, the increased expression of *Klf4* and *Gnb2* genes reached a peak at Day 10 post‐ON injury by 2.25 ± 0.16 folds (*P* < 0.001) and 2.73 ± 0.86 folds (*P* = 0.019) respectively as compared the retina before ON injury.

For the genes involved in cellular senescence, the increased expressions of *Cdkn2b* and *Tgfbr2* genes in mouse retina reached a peak at Day 7 post‐ON injury by 2.45 ± 0.01 folds (*P* < 0.001) and 3.37 ± 0.39 folds (*P* < 0.001) respectively as compared the retina before ON injury (Figure 2D). Furthermore, significantly high expression of *Cdkn1a* gene was maintained from Day 3 post‐ON injury by 5.37 ± 0.23 folds (*P* < 0.001) to Day 7 by 4.64 ± 0.28 folds (*P* < 0.001) as compared the retina before ON injury, whereas the increased expression of *Nfatc1* gene reached a peak at Day 10 by 4.31 ± 0.33 folds (*P* < 0.001).

### 3.2 Expression of Cdkn2b (p15^Ink4b^ ) and Cdkn1a (p21^Cip1^ ) protein and cellular senescence marker in mouse retina post‐optic nerve injury

**FIGURE 3:**
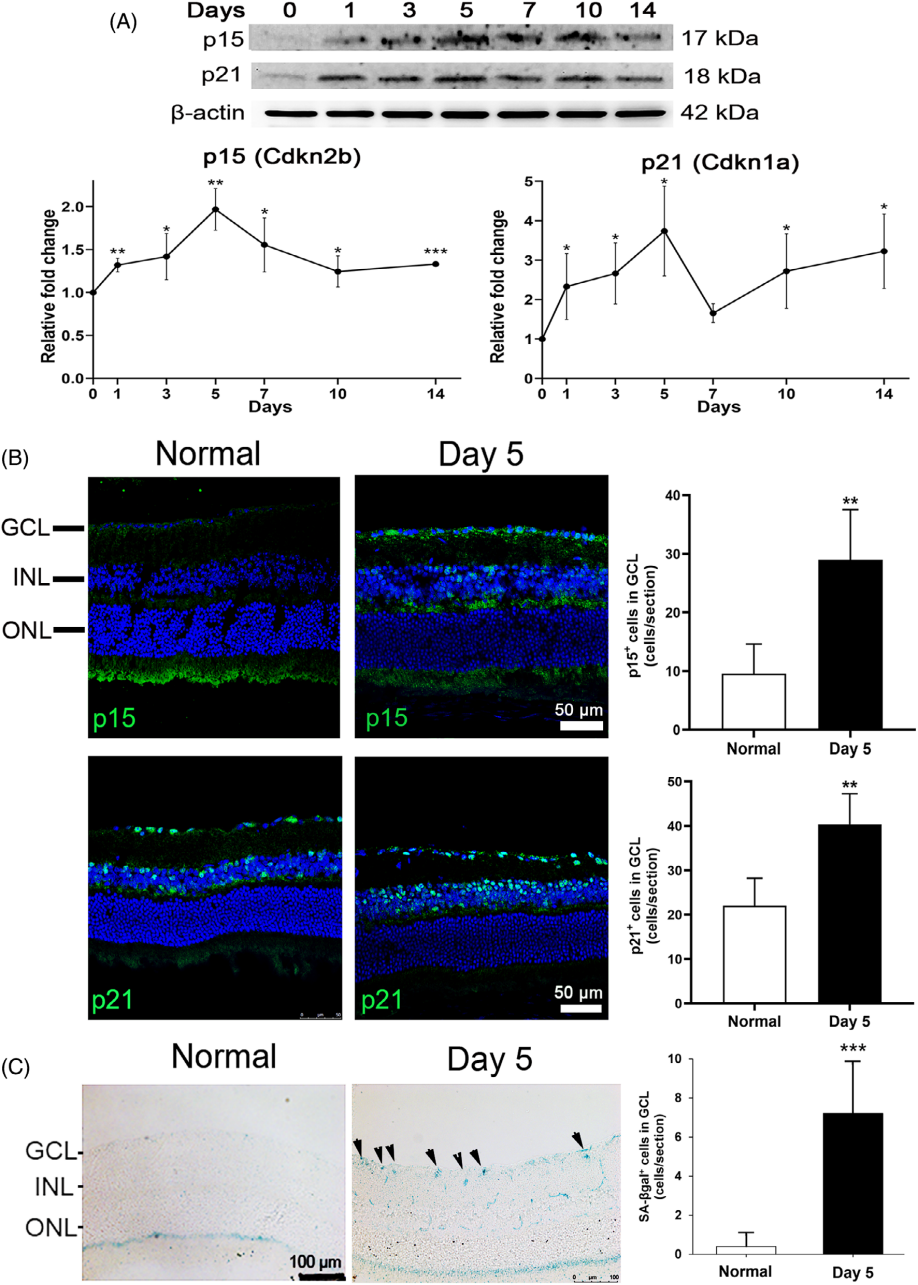
Expression of p15^Ink4b^ and p21^Cip1^ protein in mouse retina post‐optic nerve injury. (A) Immunoblotting analysis on the expression of p15^Ink4b^ and p21^Cip1^ protein in mouse retina at Day 1, 3, 5, 7, 10 and 14 post‐optic nerve (ON) injury, comparing to that before ON injury (Day 0). β‐actin was used as housekeeping protein for normalization. (B) Immunofluorescence analysis of p15^Ink4b^ and p21^Cip1^ protein in ganglion cell layer (GCL) at Day 5 post‐ON injury, comparing to that of normal mice without ON injury. Scale bar: 50 μm. Green: Target antibody signal; Blue: DAPI nuclei counter‐stain. (C) Staining on senescence‐associated β‐galactosidase (SA‐βgal) activity in GCL of mice at Day 5 post‐ON injury, comparing to that without ON injury. Arrows: SA‐βgal‐positive cells. Scale bar: 100 μm. INL, inner nuclear layer; ONL, outer nuclear layer. Data were presented as mean ± standard deviation and compared by the independent *T*‐test. *P < 0.05; **P < 0.01; ***P < 0.001.

Previous study reported that overexpression of *Cdkn2b* results in further reduction of RGC viability in N‐methyl‐d‐aspartate (NMDA)‐induced glutamate excitotoxicity in mice, while knockout of *Cdkn2b* gene attenuated the NMDA‐induced RGC death.^21^ To confirm the involvement of cellular senescence‐related genes, *Cdkn2b* and *Cdkn1a*, in ON injury, we further evaluated their protein expression (*Cdkn2b* codes for p15^Ink4b^ and *Cdkn1a* codes for p21^Cip1^) in mouse retina along different time course of ON injury. Consistent with the SYBR Green PCR analysis, immunoblotting analysis showed that the expressions of both p15^Ink4b^ and p21^Cip1^ protein in mouse retina were significantly increased since Day 1 post ON‐injury and reached a peak at Day 5 by 1.97 ± 0.24 folds (*P* = 0.007) and 3.74 ± 1.14 folds (*P* = 0.014) respectively as compared the retina before ON injury (Day 0) (Figure 3A).

To confirm the results of immunoblotting analysis, immunofluorescence analysis was conducted in mouse retinal sections at Day 5 post‐ON injury to determine their expressions in ganglion cell layer (GCL). The p15^Ink4b^‐positive cells were found to be significantly increased in GCL (29.0 ± 7.0 cells/section) at Day 5 post‐ON injury as compared to the retina of normal mice without ON injury (9.7 ± 4.1 cells/section, *P* = 0.003; Figure 3B). Similarly, the p21^Cip1^ positive cells were also significantly increased in GCL (40.3 ± 6.9 cells/section) at Day 5 post‐ON injury as compared to the retina of normal mice without ON injury (22.0 ± 5.1 cells/section, *P* = 0.005). Collectively, our results indicated that p15^Ink4b^ and p21^Cip1^ genes and protein are upregulated in mouse retina post‐ON injury.

To confirm the presence of cellular senescence in the retina after ON injury, we further examined the expression of cellular senescence marker (SA‐βgal) in the retina of mice with ON injury. Retinal section staining showed that the number of SA‐βgal‐positive cells in GCL was significantly increased in mice at Day 5 post‐ON injury (7.2 ± 2.6 cells/section) as compared to the normal mice without ON injury (0.4 ± 0.8 cells/section, *P* < 0.001; Figure 3C), indicating that premature cellular senescence occurs in the retina after ON injury.

### 3.3 LY2109761 treatment on retinal ganglion cell survival post‐optic nerve injury

**FIGURE 4:**
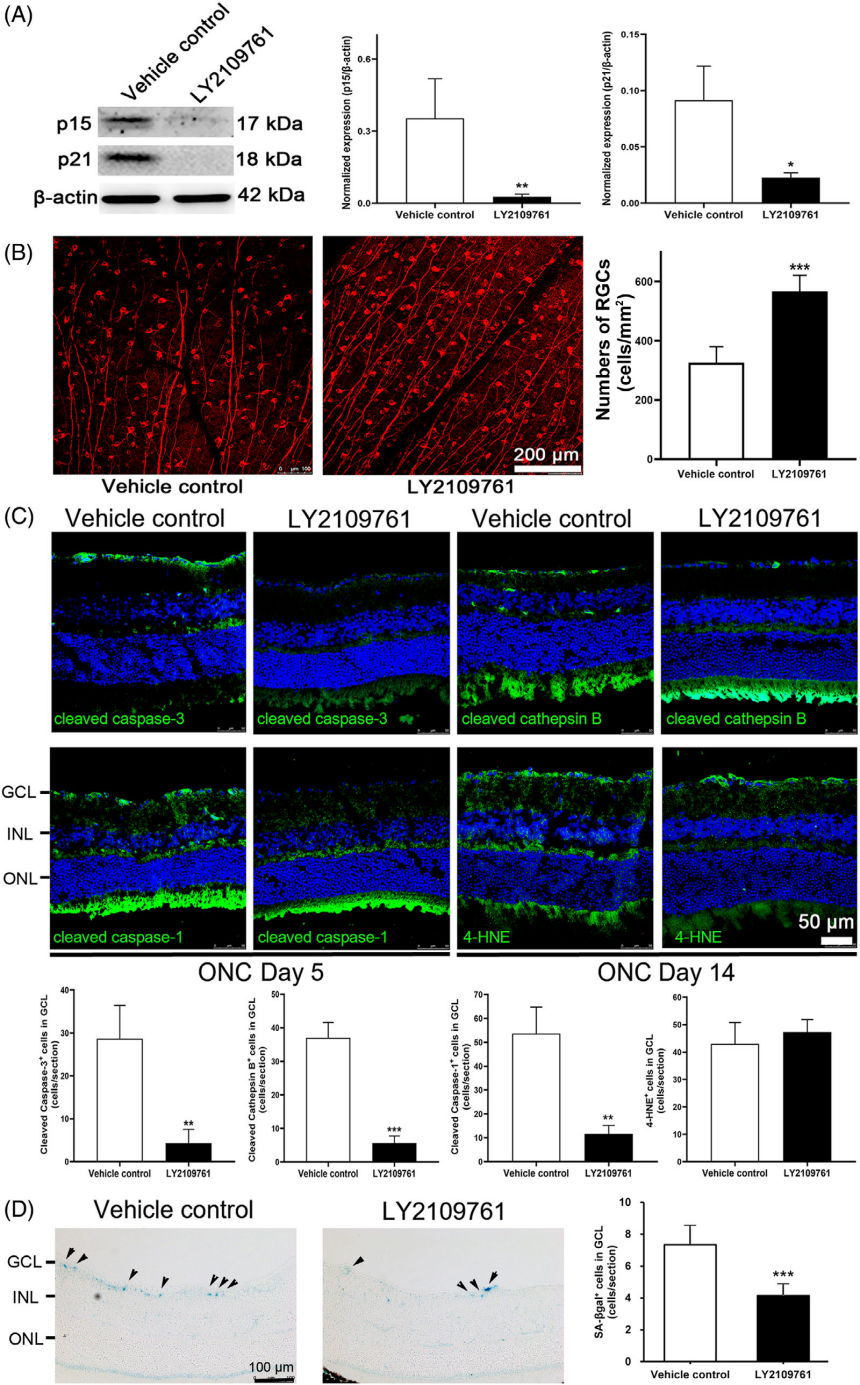
LY2109761 treatment on retinal ganglion cell survival post‐optic nerve injury. (A) Immunoblotting analysis on the expression of p15^Ink4b^ and p21^Cip1^ protein in mouse retina with LY2109761 treatment at Day 5 post‐optic nerve (ON) injury, comparing to the vehicle control treatment group. β‐actin was used as housekeeping protein for normalization. (B) Immunofluorescence analysis on the numbers of retinal ganglion cells (RGCs) in mouse retina with LY2109761 treatment at Day 14 post‐ON injury, comparing to the vehicle control treatment group. Scale bar: 200 μm. Red: β‐III tubulin signal. (C) Immunofluorescence analysis of cleaved caspase‐3 (apoptosis), cleaved cathepsin B (autolysis), cleaved caspase‐1 (pyroptosis), and 4‐hydroxynonenal (4‐HNE; ferroptosis) protein in mouse retina with LY2109761 treatment and quantification in ganglion cell layer (GCL) at Day 5 or 14 post‐ON injury, comparing to the vehicle control treatment group. Scale bar: 50 μm. Green: Target antibody signal; Blue: DAPI nuclei counter‐stain. (D) Senescence‐associated β‐galactosidase (SA‐βgal) activity in GCL of mice with LY2109761 treatment at Day 5 post‐ON injury, comparing to that of the vehicle control treatment. Arrows: SA‐βgal‐positive cells. Scale bar: 100 μm. INL, inner nuclear layer; ONL, outer nuclear layer. Data were presented as mean ± standard deviation and compared by the independent *T*‐test. *P < 0.05; **P < 0.01; ***P < 0.001.

To further confirm the involvement of p15^Ink4b^ and p21^Cip1^ in regulating RGC survival post‐ON injury, we aimed to apply the treatment targeting p15^Ink4b^ and p21^Cip1^ in mouse with ON injury; yet, there are still no specific inhibitors available targeting p15^Ink4b^ and p21^Cip1^. As previous studies reported that p15^Ink4b^ and p21^Cip1^ are commonly regulated by TGF‐β receptor,^22^, ^23^ and we found the upregulation of *Tgfbr2* gene along the time course of ON injury (Figure 2D), we selected the TGF‐β receptor inhibitor LY2109761 to target p15^Ink4b^ and p21^Cip1^ and to evaluate the effect on RGC survival. We confirmed that oral administration of LY2109761 could significantly and effectively reduce the expression of both p15^Ink4b^ and p21^Cip1^ protein in mouse retina at Day 5 post‐ON injury by 92.4% (*P* = 0.009) and 75.2% (*P* = 0.012) respectively as compared to the vehicle control treatment (Figure 4A). Immunofluorescence analysis demonstrated that the mice with LY2109761 treatment showed significantly higher number of RGCs (566.8 ± 54.1 cells/mm^2^) at Day 14 post ON‐injury than that with vehicle control treatment (326.0 ± 53.6 cells/mm^2^, *P* < 0.001, Figure 4B), indicating that downregulation of p15^Ink4b^ and p21^Cip1^ by inhibiting TGF‐β receptor can promote RGC survival post ON‐injury.

To further delineate the effect of LY2109761 treatment, the activation of the 4 major modes of cells death (apoptosis, autolysis, pyroptosis and ferroptosis) in RGCs post‐ON injury^9^ was examined. Immunofluorescence analysis demonstrated that the numbers of cleaved caspase‐3‐positive cells (apoptosis marker; 4.3 ± 3.2 cells/section, *P* = 0.007) and cleaved caspase‐1‐positive cells (pyroptosis marker; 11.7 ± 3.5 cells/section, *P* = 0.003) in GCL were both significantly decreased in mice treated with LY2109761 at Day 5 post‐ON injury as compared to that with vehicle control treatment (cleaved caspase‐3: 28.7 ± 7.8 cells/section, cleaved caspase‐1: 53.7 ± 11.1 cells/section; Figure 4C). Moreover, the number of cleaved cathepsin B‐positive cells (autolysis marker) in GCL were also significantly reduced in mice treated with LY2109761 at Day 14 post‐ON injury (5.7 ± 2.1 cells/section, *P* < 0.001) as compared to that with vehicle control treatment (37.0 ± 4.9 cells/section). Yet, there were no significant changes in the number of 4‐HNE‐positive cells (ferroptosis marker) in GCL of the mice with LY2109761 treatment (47.3 ± 4.5 cells/section) as compared to that with vehicle control treatment (43.0 ± 7.8 cells/section, *P* = 0.452).

In addition, we also evaluated the effect of LY2109761 treatment on cellular senescence. Retinal section staining showed that the number of SA‐βgal‐positive cells in GCL was significantly reduced in mice with LY2109761 treatment at Day 5 post‐ON injury (4.2 ± 0.7 cells/section) as compared to that with vehicle control treatment (7.3 ± 1.3 cells/section, *P* < 0.001; Figure 4D). Collectively, our results suggested that the RGC survival promotion post ON‐injury by inhibiting TGF‐β receptor with the downregulation of p15^Ink4b^ and p21^Cip1^ could be related to the attenuation of cellular senescence, apoptosis, autolysis and pyroptosis.

### 3.4 Senolytics treatment on retinal ganglion cell survival post‐optic nerve injury

**FIGURE 5:**
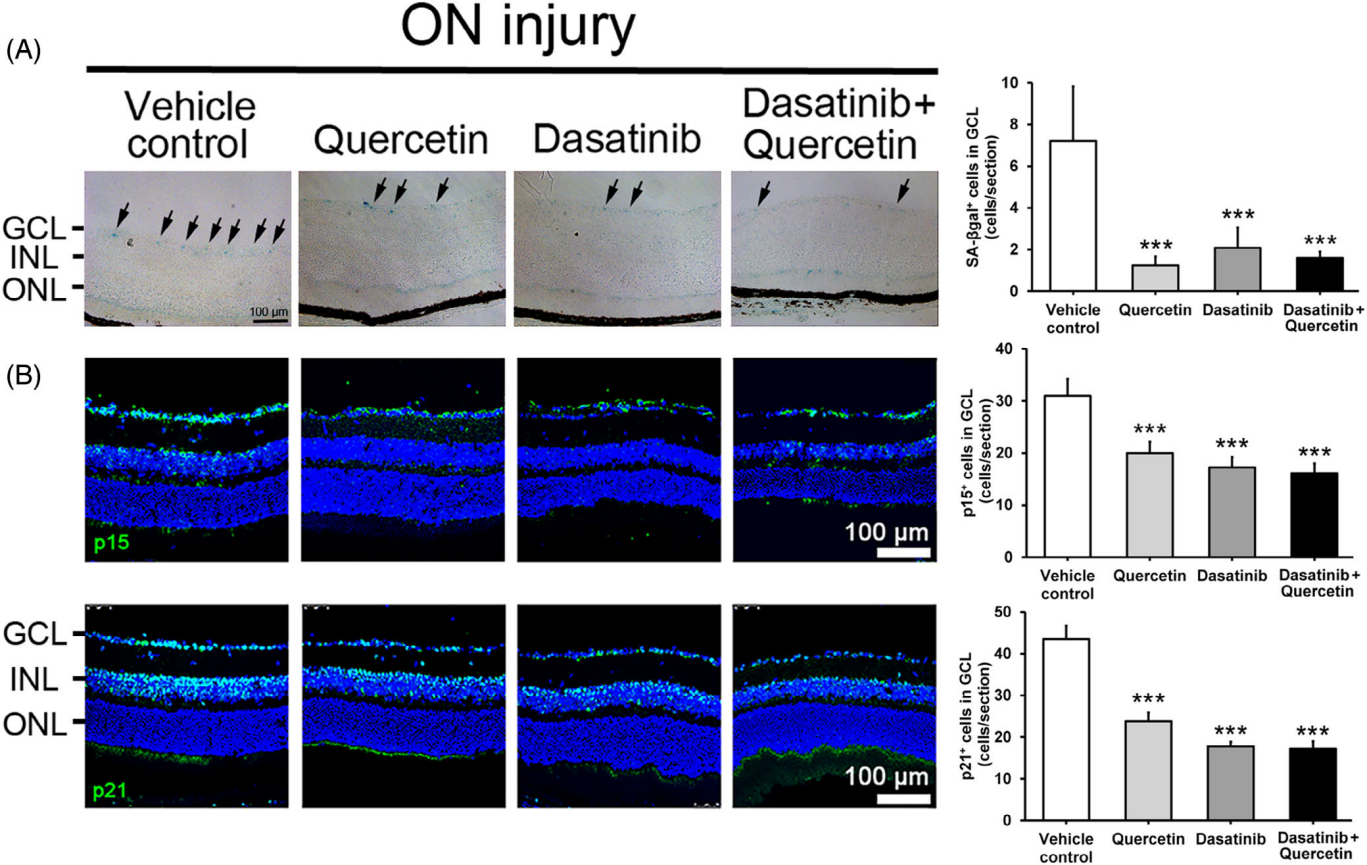
Senolytics treatment on cellular senescence marker and p15^Ink4b^ and p21^Cip1^ protein expression post‐optic nerve injury. (A) Senescence‐associated β‐galactosidase (SA‐βgal) activity in ganglion cell layer (GCL) of mice with quercetin, dasatinib and combined treatment at Day 5 post‐optic nerve (ON) injury, comparing to that of the vehicle control treatment. Arrows: SA‐βgal‐positive cells. (B) Immunofluorescence analysis on the numbers of p15^Ink4b^ and p21^Cip1^‐positive cells in GCL with quercetin, dasatinib, and combined treatment at Day 5 post‐ON injury, comparing to the vehicle control treatment group. Scale bar: 100 μm. Green: Target antibody signal; Blue: DAPI nuclei counter‐stain; INL, inner nuclear layer; ONL, outer nuclear layer. Data were presented as mean ± standard deviation and compared by one‐way analysis of variance with post hoc Tukey test. *P < 0.05; **P < 0.01; ***P < 0.001.

To confirm the involvement of cellular senescence in RGC survival regulation, we further applied the senolytics treatment to evaluate the effect of the removal of senescent cells on RGC survival. Oral administration of senolytics,^20^ quercetin alone (1.3 ± 0.4 cells/section, *P* < 0.001), dasatinib alone (2.1 ± 1.0 cells/section, *P* < 0.001), and the combined treatment (1.6 ± 0.3 cells/section, *P* < 0.001), significantly reduced the number of SA‐βgal‐positive cells in GCL at Day 5 post‐ON injury as compared to the ON‐injured mice with vehicle treatment (7.2 ± 2.6 cells/section; Figure 5A). Moreover, the numbers of p15^Ink4b^‐positive cells in GCL were significantly reduced at Day 5 post‐ON injury in mice treated with quercetin alone (20.0 ± 2.2 cells/section, *P* < 0.001), dasatinib alone (17.3 ± 2.0 cells/section, *P* < 0.001), and their combined treatment (16.1 ± 1.9 cells/section, *P* < 0.001) as compared to the vehicle control treatment (31.0 ± 3.2 cells/section; Figure 5B). Consistently, the numbers of p21^Cip1^‐positive cells in GCL were also significantly reduced at Day 5 post‐ON injury in mice treated with quercetin alone (23.8 ± 2.1 cells/section, *P* < 0.001), dasatinib alone (17.8 ± 1.1 cells/section, *P* < 0.001), and the combined treatment (17.2 ± 1.8 cells/section, *P* < 0.001) as compared to the vehicle control treatment (43.5 ± 3.3 cells/section). Collectively, our results confirmed the senolytic potentials of dasatinib and quercetin.

**FIGURE 6:**
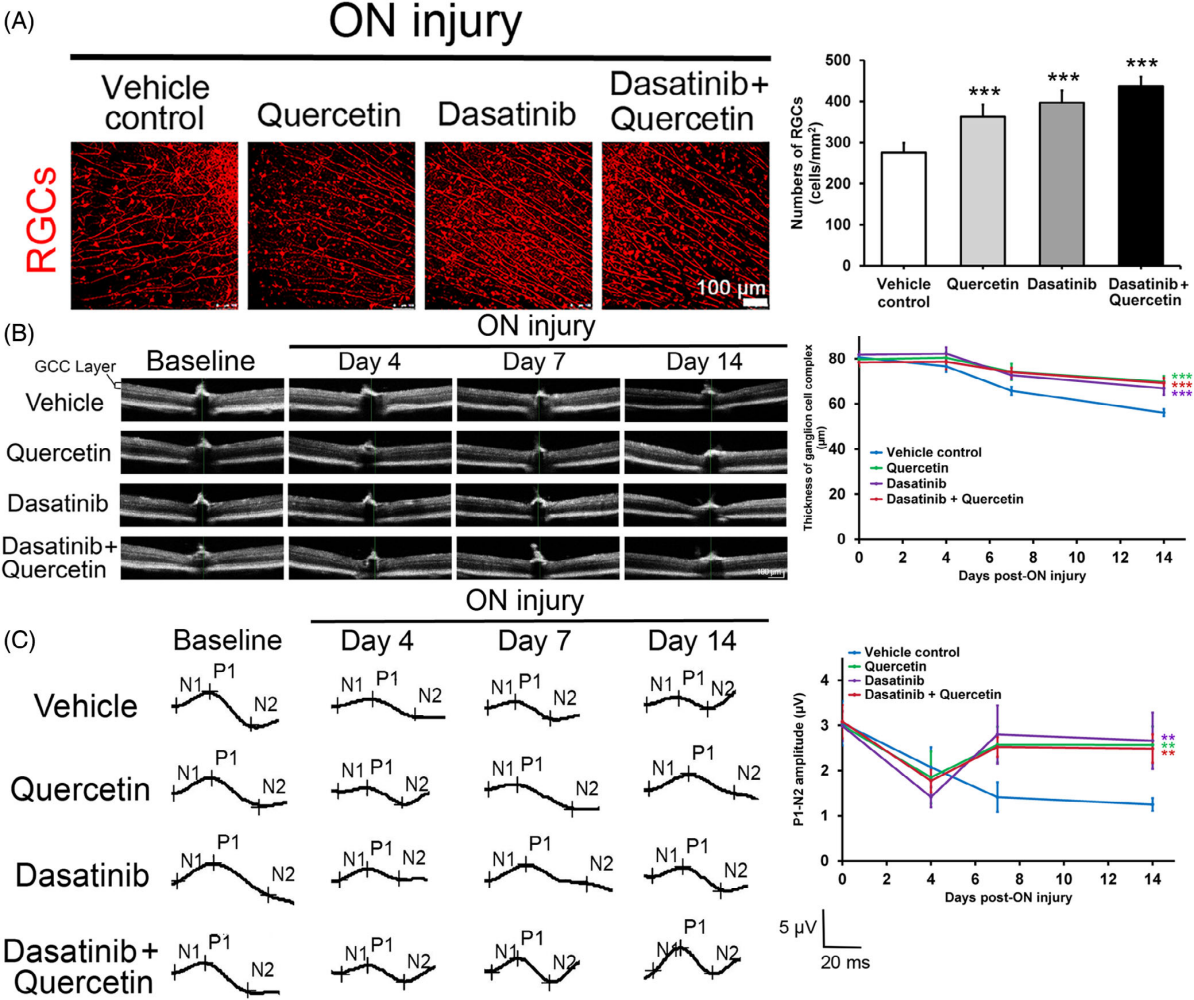
Senolytics treatment on retinal ganglion cell survival, ganglion cell complex (GCC) and pattern electroretinography post‐optic nerve injury. (A) Immunofluorescence analysis on the numbers of retinal ganglion cells (RGCs) in mouse retina with quercetin, dasatinib, and combined treatment at Day 14 post‐optic nerve (ON) injury, comparing to the vehicle control treatment group. Red: β‐III tubulin signal. Scale bar: 100 μm. (B) Spectral domain‐optical coherence tomography analysis on cross‐sectional retina of the mice with quercetin, dasatinib and combined treatment in vivo at baseline and at Day 4, 7 and 14 post‐ON injury, comparing to that of the vehicle control treatment. The thickness of GCC, composing of retinal nerve fibre layer, ganglion cell layer, and inner plexiform layer, was measured. Vertical and horizontal scale bar: 100 μm. (C) Pattern electroretinography analysis on mice with quercetin, dasatinib, and combined treatment in vivo at baseline and at Day 4, 7 and 14 post‐ON injury, comparing to that of the vehicle control treatment. The first positive peak in the waveform was designated as P1 and the second negative peak as N2. The amplitude was measured from P1 to N2. Vertical scale bar: 5 μV. Horizontal scale bar: 20 ms. Data were presented as mean ± standard deviation and compared by one‐way analysis of variance with post hoc Tukey test. **P < 0.01; ***P < 0.001.

Immunofluorescence analysis demonstrated that the numbers of RGCs were significantly increased in mice with treatment of quercetin alone (363.0 ± 29.0 cells/mm^2^, *P* < 0.001), dasatinib alone (396.8 ± 29.5 cells/mm^2^, *P* < 0.001), and combined treatment (437.3 ± 23.2 cells/mm^2^, *P* < 0.001) at Day 14 post‐ON injury as compared to the vehicle control group (275.8 ± 23.2 cells/mm^2^; Figure 6A). Longitudinal in vivo measurement of retinal thickness by OCT showed that the GCC thickness gradually decreased in the mouse retina along the ON injury from 80.61 ± 0.82 μm at baseline to 56.14 ± 1.59 μm at Day 14 (Figure 6B). Consistently, the GCC thickness was significantly higher in mice with treatment of quercetin alone (69.76 ± 2.55 μm, *P* < 0.001), dasatinib alone (66.92 ± 2.86 μm, *P* < 0.001), and combined treatment (69.27 ± 2.27 μm, *P* < 0.001) at Day 14 post‐ON injury as compared to the vehicle control group. For the pattern ERG analysis, the P1‐N2 amplitude gradually decreased along the ON injury from 3.05 ± 0.49 μV at baseline to 1.25 ± 0.14 μV at Day 14 (Figure 6C). The P1‐N2 amplitude was significantly higher in mice with treatment of quercetin alone (2.57 ± 0.41 μV, *P* = 0.002), dasatinib alone (2.66 ± 0.62 μV, *P* = 0.001), and combined treatment (2.49 ± 0.31 μV, *P* = 0.005) at Day 14 post‐ON injury as compared to the vehicle control group. Collectively, our results demonstrated that the senolytics treatment targeting cellular senescence can promote RGC survival and function in mice with ON injury.

**FIGURE 7:**
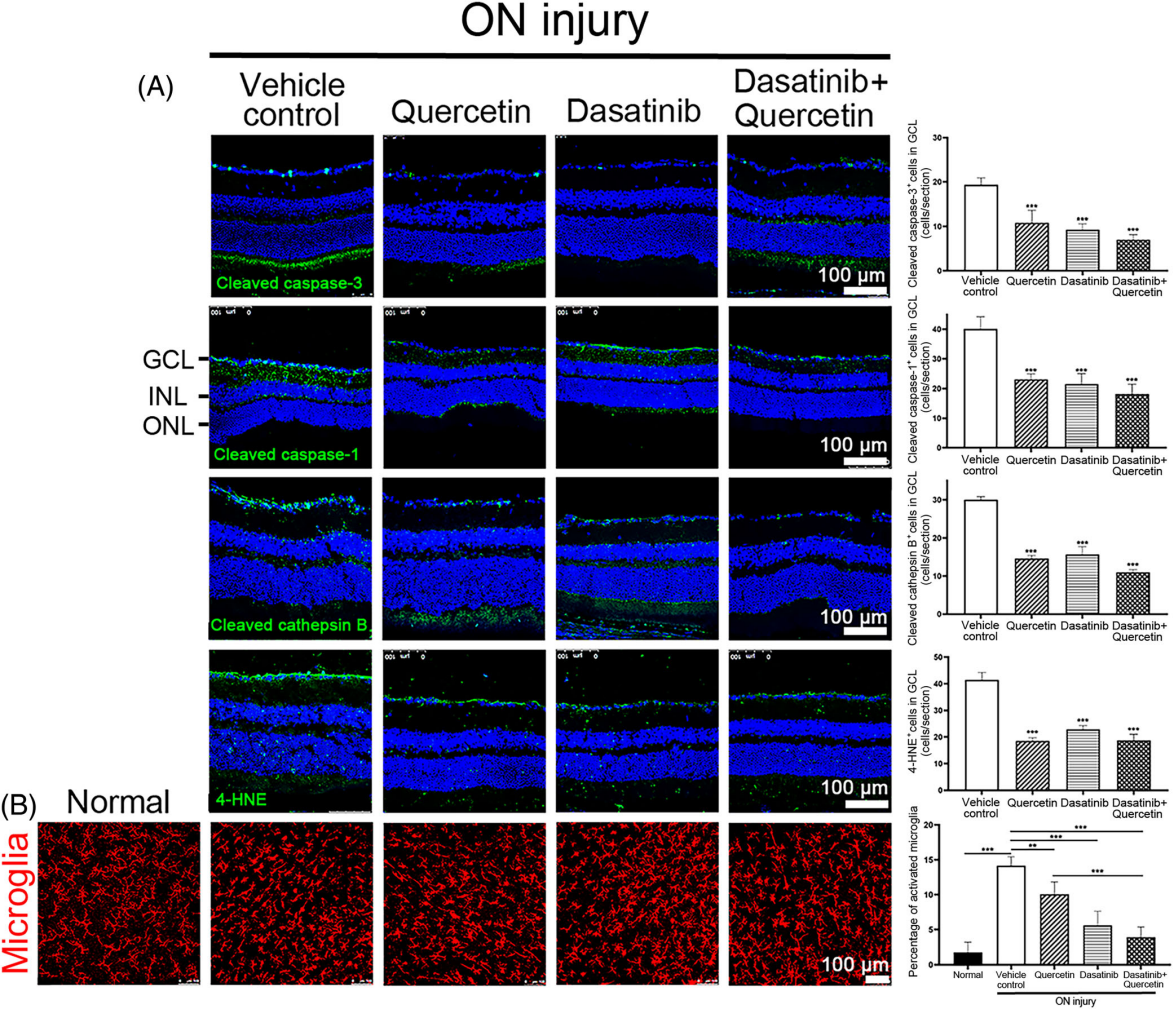
Senolytics treatment on the modes of cell death in retinal ganglion cells and microglia activation post‐optic nerve injury. (A) Immunofluorescence analysis of cleaved caspase‐3 (apoptosis), cleaved cathepsin B (autolysis), cleaved caspase‐1 (pyroptosis) and 4‐hydroxynonenal (4‐HNE; ferroptosis) protein in ganglion cell layer (GCL) of mice with quercetin, dasatinib, and combined treatment at Day 5 (cleaved caspase‐3 and cleaved caspase‐1) or 14 (cleaved cathepsin B and 4‐HNE) post‐ON injury, comparing to the vehicle control treatment group. (B) Immunofluorescence analysis on the percentage of activated microglia in mouse retina with quercetin, dasatinib, and combined treatment at Day 7 post‐ON injury, comparing to that of normal mice without ON injury and the vehicle control treatment. Resting microglia are ramified in shape, and the activated microglia acquire an amoeboid cell shape. Red: Iba signal. Scale bar: 100 μm. Green: Target antibody signal; Blue: DAPI nuclei counter‐stain; INL, inner nuclear layer; ONL, outer nuclear layer. Data were presented as mean ± standard deviation and compared by one‐way analysis of variance with post hoc Tukey test. **P < 0.01; ***P < 0.001.

To further delineate the effect of senolytics treatment in mice with ON injury, the activation of the 4 major modes of cells death in RGCs post‐ON injury was examined. Immunofluorescence analysis showed that the numbers of cleaved caspase‐3‐positive cells in GCL were significantly decreased at Day 5 post‐ON injury in mice treated with quercetin alone (10.8 ± 2.7 cells/section, *P* < 0.001), dasatinib alone (9.3 ± 1.3 cells/section, *P* < 0.001), and the combined treatment (7.0 ± 1.1 cells/section, *P* < 0.001) as compared to the vehicle control treatment (19.3 ± 1.5 cells/section; Figure 7A). The numbers of cleaved caspase‐1‐positive cells in GCL were also significantly decreased at Day 5 post‐ON injury in mice treated with quercetin alone (23.1 ± 1.9 cells/section, *P* < 0.001), dasatinib alone (21.5 ± 3.6 cells/section, *P* < 0.001), and the combined treatment (18.2 ± 3.3 cells/section, *P* < 0.001) as compared to the vehicle control treatment (40.1 ± 4.1 cells/section). Moreover, the number of cleaved cathepsin B‐positive cells in GCL were significantly decreased at Day 5 post‐ON injury in mice treated with quercetin alone (14.6 ± 0.8 cells/section, *P* < 0.001), dasatinib alone (15.7 ± 2.1 cells/section, *P* < 0.001), and the combined treatment (11.0 ± 0.7 cells/section, *P* < 0.001) as compared to the vehicle control treatment (30.0 ± 0.8 cells/section). The number of 4‐HNE‐positive cells in GCL were significantly decreased at Day 5 post‐ON injury in mice treated with quercetin alone (18.5 ± 1.2 cells/section, *P* < 0.001), dasatinib alone (22.9 ± 1.4 cells/section, *P* < 0.001), and the combined treatment (18.7 ± 2.3 cells/section, *P* < 0.001) as compared to the vehicle control treatment (41.4 ± 2.8 cells/section). In addition, we found that the percentage of activated microglia in mouse retina was significantly increased in mice at Day 7 post‐ON injury (14.2 ± 1.3%, *P* < 0.001) as compared to the normal mice without ON injury (1.8 ± 1.5%; Figure 7B). Treatment of quercetin alone (10.1 ± 1.8%, *P* = 0.004), dasatinib alone (5.6 ± 2.0%, *P* < 0.001), and the combined treatment (3.9 ± 1.5%, *P* < 0.001) significantly reduced the percentage of activated microglia in mouse retina at Day 7 post‐ON injury as compared to the ON‐injured mice with vehicle treatment.

## 4 DISCUSSION

Results from this study showed that: (1) The genes involved in the immune response, cellular senescence, pyroptosis, and apoptosis were differentially expressed in rodent retina with ON injury; (2) Cellular senescence markers were upregulated in mouse retina with ON injury; (3) LY2109761 treatment could promote RGC survival post‐ON injury by reducing the expressions of p15^Ink4b^, p21^Cip1^, cellular senescence, and cell death markers; (4) Dasatinib and quercetin treatments could promote RGC survival and alleviate the reduction of GCC thickness and pattern ERG activity post‐ON injury by reducing cellular senescence and cell death marker expression and the activation of microglia. Collectively, this study demonstrated the involvement of premature cellular senescence in RGC loss post‐ON injury.

Retinal transcriptome analysis by RNA sequencing on mouse retina at Day 2 post‐ON crush surgery identified that the differentially expressed genes are involved in endoplasmic reticulum stress, anti‐oxidative response and immune response.^24^ Another RNA sequencing analysis on mouse retina at Day 3 post‐ON crush surgery also revealed the differentially expressed genes involved in inflammation‐related pathways, cell cycle and apoptosis.^25^ Moreover, subtractive hybridization analysis on mouse retina at 8 h post‐ON crush surgery identified the differentially expressed genes involved in apoptosis, immune and inflammation, axon guidance, and energy and metabolism.^26^ Consistent with previous reports, in this study, our RNA sequencing analysis on rat retina at Day 7 post‐ON injury identified that the differentially expressed genes involved in the innate immune response, complement pathway, NAD^+^ nucleosidase activity, NLRP3 inflammasome complex, cellular senescence, and apoptosis (Table 1).We also validated the expression of the identified differentially expressed genes related to inflammation, neuroprotection, axonal regeneration, and cellular senescence in mouse retina along different time course of ON injury (Figure 2). Previous studies reported that around 50% RGC loss at 7 days after ON injury in both mouse and rat models,^27^ indicating similar course and regulatory signals of RGC death between the rats and mice after ON injury. The cross‐species validation can help to consolidate the gene expression pattern in different species.^28^ Our results indicated that rats and mice share common pathophysiological pathways regulating RGC death in ON injury.

From the gene ontology analysis of the differentially expressed genes identified by the RNA sequencing analysis, we notably observed a cluster of 24 genes related to cellular senescence (Table 1). Cellular senescence is a cellular stress response and characterized by a set of core features, including durable growth arrest, increased lysosomal b‐galactosidase activity, expression of anti‐proliferative molecules, activation of damage sensing signalling pathways, and the production of pro‐inflammatory cytokines (refers to the senescence‐associated secretory phenotype [SASP]).^29^ Apart from contributing to aging and age‐related diseases,^30^ senescent cells are also recently reported in trauma conditions, such as spinal cord injury,^31^ but not studied in ON injury. In this study, we observed that cellular senescence‐related anti‐proliferative genes, *Cdkn2b* and *Cdkn1a*, and their encoded proteins, p15^Ink4b^ and p21^Cip1^ respectively, were significantly upregulated in mouse retina post‐ON injury (Figure 2D and 3). *CDKN2B* is expressed in human iPSC‐derived RGCs,^32^ and the *CDKN2B* gene variants are associated with primary angle open glaucoma.^33^, ^34^ Notably, *Cdkn2b* overexpression reduces RGC survival in mouse model of NMDA‐induced glutamate excitotoxicity, while knockout of *Cdkn2b* gene attenuated the NMDA‐induced RGC death.^21^ Collectively, our results and other reports together suggest that *Cdkn2b* (p15^Ink4b^) should be involved in regulating RGC survival in ON injury. In contrast, *Cdkn1a* (p21^Cip1^) has still not been reported to be involved in ON injury. To confirm the involvement of p15^Ink4b^ and p21^Cip1^ in RGC survival regulation post‐ON injury, we aimed to inhibit p15^Ink4b^ and p21^Cip1^ in mouse with ON injury; yet, there are no specific inhibitors available targeting p15^Ink4b^ and p21^Cip1^. Coincidently, p15^Ink4b^ and p21^Cip1^ are commonly regulated by TGF‐β receptor^22^, ^23^ that p15^Ink4b^ and p21^Cip1^ are upregulated by TGF‐β via TGF‐β receptor‐I and TGF‐β receptor‐II,^35^ and we observed the upregulation of *Tgfbr2* gene post‐ON injury (Figure 2D); therefore, the TGF‐β receptor inhibitor LY2109761 was selected to evaluate the effect on RGC survival in mice with ON injury. We demonstrated that LY2109761 effectively reduced the expression of both p15^Ink4b^ and p21^Cip1^ protein in mouse retina and significantly promote RGC survival post‐ON injury (Figure 4). Collectively, our results indicated that the upregulation of p15^Ink4b^ and p21^Cip1^ is associated with RGC death post‐ON injury, while the downregulation of p15^Ink4b^ and p21^Cip1^ is associated with the promotion of RGC survival.

Apart from the upregulation of the anti‐proliferative genes, we also observed the increased expression of additional cellular senescence marker (SA‐βgal) in mouse retina post‐ON injury (Figure 3C), confirming the involvement of cellular senescence in ON injury. A transgenic mouse model with early removal of senescent cells as well as dasatinib have been shown to protect RGCs from senescence and apoptosis in mice upon intraocular pressure elevation.^36^ Moreover, quercetin has also been used to improve RGC survival in ischemia injury and glaucoma rat model.^37^, ^38^ Yet, the strategy of senescent cell removal has not been applied in ON injury. Senolytics are compounds that selectively eliminate senescent cells while not damaging normal cells.^39^ Dasatinib and quercetin have been applied as senolytics to selectively eliminate the senescent cells in old mice.^20^ We confirmed the senolytic property of dasatinib and quercetin that dasatinib and quercetin as well as their combined treatment can effectively reduce the numbers of SA‐βgal, p15^Ink4b^ and p21^Cip1^‐positive cells in GCL post‐ON injury (Figure 5A, B). Dasatinib and quercetin treatments can significantly promote the survival of RGCs and alleviate the reduction of GCC thickness and pattern ERG activity post‐ON injury (Figure 6). Collectively, our results indicated that cellular senescence is associated with RGC death post‐ON injury, and removal of senescent cells is associated with the promotion of RGC survival.

Inflammation is a core program of the SASP.^40^ In this study, we observed the clusters of the differentially expressed genes involved in immune response, complement pathway and NLRP3 inflammasome complex in rat retina with ON injury (Table 1). We also confirm the upregulation of inflammation‐related genes and the activation of microglia in mouse retina with ON injury (Figure 2A and 5E), indicating the increased inflammation in rodent retina post‐ON injury and reflecting the activation of the SASP and cellular senescence. Pyroptosis is a proinflammatory and lytic mode of cell death.^41^ Reducing the activation of pyroptosis has been shown to be associated with the alleviation of RGC damage after ON crush surgery.^42^ In this study, we demonstrated the reduction in the numbers of cleaved caspase‐1‐positive cells (pyroptosis marker) and activated microglia in mouse retina post‐ON injury with the treatments of dasatinib, quercetin, and LY2109761 (Figure 4C and 7), suggesting the reduction of the inflammatory SASP program by targeting cellular senescence and its association with the RGC survival promotion post‐ON injury.

There were several limitations in this study. First, the direct inhibition of p15^Ink4b^ and p21^Cip1^ was not examined as there are still no specific inhibitors available targeting p15^Ink4b^ and p21^Cip1^. Instead, we selected the TGF‐β receptor inhibitor LY2109761 to target p15^Ink4b^ and p21^Cip1^ as p15^Ink4b^ and p21^Cip1^ can be commonly regulated by TGF‐β receptor.^22^, ^23^ Our results demonstrated that LY2109761 can effectively reduce the expression of both p15^Ink4b^ and p21^Cip1^ protein in mouse retina post‐ON injury (Figure 4A). Second, whole retina was used in the RNA sequencing, gene expression and immunoblotting analyses. In this study, we confirmed the expression in GCL by immunofluorescence analysis in retinal sections. Nevertheless, co‐staining of cellular senescence and cell death markers with the RGC marker can help to confirm their expressions in RGCs.

In summary, results from this study revealed that premature cellular senescence mediates RGC survival regulation post‐ON injury with the modulation of the major modes of cell death and microglia activation. Senolytics and TGF‐β receptor inhibitor can be the potential treatment strategies promoting RGC survival in traumatic optic neuropathy.

## AUTHOR CONTRIBUTIONS

T.K.N. conception and design. T.K.N. financial support. T.K.N. provision of study materials. Y.Y., X.B., Y.X., Sh. C., Si C. and Y.C. collection and/or assembly of data. Y.Y., X.B. and Y.X. data analysis and interpretation. Y.Y., X.B. and T.K.N. manuscript writing.

## FUNDING INFORMATION

This study was supported by National Natural Science Foundation of China (project code: 82371049 to T.K.N.) and Natural Science Foundation of Guangdong Province (grant number: 2023A1515010195 to T.K.N.), China.

## CONFLICT OF INTEREST STATEMENT

The authors declare no conflicts of interest.

## Supporting information


**Data S1.** Supporting Information.

## Data Availability

The data that support the findings of this study are available from the corresponding author upon reasonable request.
