# Supplementary material for: Cellular senescence mediates retinal ganglion cell survival regulation post‐optic nerve crush injury
Source: Cell Prolif. 2024 Jul 18;57(12):e13719. doi: 10.1111/cpr.13719 (PMC11628747; doi:10.1111/cpr.13719)
Supplement: Supplementary file 1 — Data S1. Supporting Information. [file CPR-57-e13719-s001.pdf]

Supplementary table 1: Primers for gene expression analysis

Genes		Sequence (5'>3')	Annealing Temp (ºC)	Amplicon size (bp)	References
Csf1r	F:	GTCATGTCTCTGCTGGTGCT	60	139	NM_001037859.2
	R:	AGGGCAACTGAGTAGGGTCA			
Tlr9	F:	AGTGTCACTTCCTCAATTCTCTGA	60	117	NM_031178.2
	R:	TCGACGGAGAACCATGTTGG			
Tlr7	F:	AGAAAGATGTCCTTGGCTCCC	60	127	NM_133211.4
	R:	TCCGTGTCCACATCGAAAACA			
Socs3	F:	GTAGACTTCACGGCTGCCAA	60	129	NM_007707.3
	R:	GCATCCCGGGGAGCTAGT			
Cd38	F:	AAACTACAGGCCTGGGTGATG	60	184	NM_007646.5
	R:	GTCTACACGATGGGTGCTCA			
Klf4	F:	CCAAGCCAAAGAGGGGAAGA	60	159	NM_010637.3
	R:	CGTCCCAGTCACAGTGGTAA			
Tgfbr2	F:	GATGTGGAAATGGAAGCCCAGA	60	160	NM_009371.3
	R:	TGGACAGTCTCACATCGCAAA			
Nlrp3	F:	GCCTCACTGAACTGGACCTC	60	151	NM_145827.4
	R:	GAGGAGATGTCGAAGCAGCA			
Pycard	F:	TGAGCAGCTGCAAACGACTA	60	100	NM_023258.4
	R:	CTGGTCCACAAAGTGTCCTGT			
Cdkn2b	F:	GCAGATCCCAACGCCCTG	60	181	NM_007670.4
	R:	CGACAAGCGTGTCCAGGAA			
Nfatc1	F:	CCCGGAGTTCGACTTCGATT	60	193	NM_016791.4
	R:	GGCTGAAGGAACAGCTGAGA			
Cdkn1a	F:	CGGTGTCAGAGTCTAGGGGAA	60	106	NM_007669.5
	R:	AACAGGTCGGACATCACCAG			
Gnb2	F:	CTTGGCAGAGTCCCACCAC	60	134	NM_010312.5
	R:	CAGTTGCTCCAGCTCACTCA			
Sprr1a	F:	GAGAACCTGCTCTTCTCTGAGT	60	118	NM_009264.2
	R:	AGGCTGCTTCACCTGCTG			
Ecel1	F:	GAAGCTGGTGCATCACTCTCA	60	192	NM_021306.3
	R:	GGAATACGGGGCCTCCATTG			
Flnc	F:	TCCGCCAAATGAAGCTGGAA	60	200	NM_001081185.2
	R:	TTTGCGGGCATCCTCATCAT			
Klf5	F:	ATCTGAAACACGCGCACCA	60	182	NM_009769.4
	R:	GTCTACCACTGAGGCACTGTC			
Top2a	F:	AGCCCATTGGTCAGTTTGGA	60	104	NM_011623.2
	R:	AGGAAACAACAACCGAGCCA			
Lgals3	F:	GCGGGTGGAGCACTAATCA	60	145	NM_010705.3
	R:	GATAAGCAGCCCCTGGGTAG			
Rac2	F:	GCAATGCAGGCCATCAAGTG	60	159	NM_009008.3
	R:	GTTCACCGGCTTACTGTCCA			
Nrn1	F:	GGGACTTAAGTTGAACGGCAGA	60	199	NM_153529.2
	R:	TGTGCACACGGTCTTGATGT			
Actb	F:	GCTCCGGCATGTGCAAAG	60	100	NM_007393.5
	R:	CCCACCATCACACCCTGG			

F: forward primer; R: reverse primer; Temp: temperature; bp: base pairs

Supplementary table 2: Upregulated differentially expressed genes in rat retina with optic nerve injury.

Gene identity	Gene name	log_2 Fold change	P_{corr}
ENSRNOG00000037085	*Xirp1*	9.57	1.33×10^{-7}
ENSRNOG00000024028	*Sprr1a*	9.36	4.70×10^{-10}
ENSRNOG00000015024	*Mcoln3*	8.63	1.44×10^{-6}
ENSRNOG00000022640	*AABR07003235.1*	8.46	1.62×10^{-6}
ENSRNOG00000003069	*Cd38*	8.31	5.78×10^{-6}
ENSRNOG00000019447	*Ecel1*	8.08	8.41×10^{-15}
ENSRNOG00000022565	*Lrrc25*	7.90	3.17×10^{-7}
ENSRNOG00000037409	*Scimp*	7.81	2.94×10^{-3}
ENSRNOG00000006090	*Ucn*	7.80	3.03×10^{-4}
ENSRNOG00000007281	*Flnc*	7.66	1.29×10^{-24}
ENSRNOG00000022710	*Prrg4*	7.66	4.97×10^{-5}
ENSRNOG00000054286	*Rrm2*	7.40	2.64×10^{-3}
ENSRNOG00000016632	*Dsg3*	7.28	1.87×10^{-3}
ENSRNOG00000018681	*Nes*	7.24	7.18×10^{-6}
ENSRNOG00000008785	*Klf5*	7.21	4.95×10^{-5}
ENSRNOG00000036673	*Sectm1b*	7.14	2.44×10^{-4}
ENSRNOG00000030012	*Clec4a2*	7.08	1.28×10^{-2}
ENSRNOG00000004498	*Scin*	7.08	1.71×10^{-2}
ENSRNOG00000000640	*Egr2*	7.07	3.02×10^{-3}
ENSRNOG00000053047	*Top2a*	7.04	1.50×10^{-5}
ENSRNOG00000055185	*Dusp9*	7.01	2.76×10^{-2}
ENSRNOG00000060235	*AABR07044837.2*	7.00	1.02×10^{-4}
ENSRNOG00000009528	*Sdcbp2*	6.89	1.18×10^{-4}
ENSRNOG00000037931	*Plaur*	6.83	2.07×10^{-6}
ENSRNOG00000058862	*AABR07051308.1*	6.78	3.27×10^{-3}
ENSRNOG00000061316	*LOC102547056*	6.74	1.20×10^{-2}
ENSRNOG00000013598	*Melk*	6.71	1.77×10^{-2}
ENSRNOG00000007159	*Ccl2*	6.71	4.35×10^{-3}
ENSRNOG00000021266	*Prokr2*	6.51	1.98×10^{-2}
ENSRNOG00000059947	*Sdc1*	6.50	1.41×10^{-9}
ENSRNOG00000036703	*Itgax*	6.47	1.68×10^{-4}
ENSRNOG00000025460	*Tmem71*	6.47	1.44×10^{-2}
ENSRNOG00000025787	*Spag6l*	6.46	1.65×10^{-2}
ENSRNOG00000002309	*Hlx*	6.43	1.02×10^{-4}
ENSRNOG00000010645	*Lgals3*	6.42	1.49×10^{-7}
ENSRNOG00000000187	*Csf2rb*	6.40	4.48×10^{-11}
ENSRNOG00000062144	*AABR07035955.1*	6.39	4.66×10^{-3}
ENSRNOG00000021586	*Gpr39*	6.36	2.74×10^{-3}
ENSRNOG00000037853	*Rarres1*	6.33	7.49×10^{-7}
ENSRNOG00000047657	*C4a*	6.32	5.48×10^{-5}
ENSRNOG00000015773	*Il21r*	6.32	3.00×10^{-4}
ENSRNOG00000048881	*LOC680910*	6.23	2.48×10^{-2}
ENSRNOG00000050430	*Vav1*	6.22	2.41×10^{-8}
ENSRNOG00000042944	*Cenpw*	6.20	3.83×10^{-2}
ENSRNOG00000010478	*Serpina3n*	6.19	1.67×10^{-22}

ENSRNOG00000023303	*Dpep2*	6.18	3.54×10^{-3}
ENSRNOG00000056219	*Olr1*	6.15	1.40×10^{-8}
ENSRNOG00000026306	*Clec5a*	6.15	6.14×10^{-3}
ENSRNOG00000014464	*Tnfsf13b*	6.14	1.92×10^{-3}
ENSRNOG00000021424	*Cd300lf*	6.14	4.92×10^{-5}
ENSRNOG00000007002	*Lif*	6.12	1.01×10^{-2}
ENSRNOG00000012723	*Trim55*	6.10	7.85×10^{-3}
ENSRNOG00000048161	*Tlr9*	6.08	8.94×10^{-5}
ENSRNOG00000058337	*Plcb2*	6.07	5.69×10^{-5}
ENSRNOG00000025051	*Tal1*	6.00	2.75×10^{-5}
ENSRNOG00000003806	*Gpr65*	5.97	2.49×10^{-3}
ENSRNOG00000045558	*Cd34*	5.87	2.72×10^{-9}
ENSRNOG00000000195	*LOC108348048*	5.85	2.19×10^{-5}
ENSRNOG00000020991	*Ms4a6a*	5.83	7.97×10^{-6}
ENSRNOG00000032844	*RT1-Da*	5.83	9.47×10^{-7}
ENSRNOG00000014156	*Fut7*	5.83	1.19×10^{-3}
ENSRNOG00000022256	*Cxcl10*	5.82	1.51×10^{-4}
ENSRNOG00000000655	*Ptprc*	5.78	5.59×10^{-9}
ENSRNOG00000024390	*Osm*	5.75	3.18×10^{-2}
ENSRNOG00000054964	*Aoah*	5.72	2.27×10^{-3}
ENSRNOG00000016575	*Tnfrsf1b*	5.64	9.68×10^{-6}
ENSRNOG00000033348	*Duox1*	5.64	6.23×10^{-5}
ENSRNOG00000029662	*Wdfy4*	5.63	1.54×10^{-5}
ENSRNOG00000030688	*Lrrc2*	5.60	1.28×10^{-2}
ENSRNOG00000025948	*Inava*	5.60	3.57×10^{-2}
ENSRNOG00000039048	*Cd200r1*	5.59	5.89×10^{-4}
ENSRNOG00000020401	*Adcy4*	5.58	7.37×10^{-4}
ENSRNOG00000042139	*Clec4a1*	5.56	1.18×10^{-5}
ENSRNOG00000061005	*AABR07016572.1*	5.56	2.68×10^{-3}
ENSRNOG00000006778	*Mmp19*	5.55	7.47×10^{-12}
ENSRNOG00000021161	*Fermt3*	5.54	5.93×10^{-7}
ENSRNOG00000023428	*Pik3r5*	5.54	7.49×10^{-7}
ENSRNOG00000048914	*Traf1*	5.53	1.29×10^{-2}
ENSRNOG00000061595	*Tfec*	5.53	7.97×10^{-4}
ENSRNOG00000012216	*Tgfbi*	5.49	5.75×10^{-18}
ENSRNOG00000013240	*Ptger4*	5.47	2.05×10^{-2}
ENSRNOG00000036959	*RGD1561551*	5.47	2.08×10^{-3}
ENSRNOG00000053037	*AABR07024139.1*	5.46	4.76×10^{-2}
ENSRNOG00000007350	*Rac2*	5.45	4.70×10^{-10}
ENSRNOG00000014683	*Il1rl2*	5.44	4.08×10^{-2}
ENSRNOG00000011680	*Il16*	5.42	1.00×10^{-6}
ENSRNOG00000001476	*Cldn4*	5.40	4.00×10^{-7}
ENSRNOG00000058568	*Dhrs9*	5.39	2.50×10^{-5}
ENSRNOG00000015366	*Neurl3*	5.36	9.06×10^{-3}
ENSRNOG00000028768	*Gbp4*	5.36	1.09×10^{-2}
ENSRNOG00000005810	*Nlrc4*	5.35	1.08×10^{-4}
ENSRNOG00000005008	*Angpt4*	5.29	7.53×10^{-6}
ENSRNOG00000009211	*C3ar1*	5.27	1.57×10^{-5}
ENSRNOG00000018735	*Cd74*	5.26	1.11×10^{-7}

ENSRNOG00000025094	*Gpr183*	5.24	3.47×10^{-8}
ENSRNOG00000046663	*LOC498276*	5.22	1.70×10^{-4}
ENSRNOG00000006996	*Mms22l*	5.22	2.71×10^{-3}
ENSRNOG00000020465	*Ripk3*	5.22	9.27×10^{-5}
ENSRNOG00000033697	*Casp4*	5.21	3.69×10^{-16}
ENSRNOG00000061857	*Mgst2*	5.20	2.98×10^{-3}
ENSRNOG00000047363	*Kcnk13*	5.20	6.33×10^{-5}
ENSRNOG00000054954	*Lilrb2*	5.20	3.22×10^{-3}
ENSRNOG00000010972	*Neurog2*	5.16	1.92×10^{-3}
ENSRNOG00000003748	*RGD1565785*	5.15	1.32×10^{-3}
ENSRNOG00000026371	*Krt17*	5.14	4.22×10^{-2}
ENSRNOG00000026518	*Tmem156*	5.14	5.87×10^{-3}
ENSRNOG00000055716	*Oscar*	5.13	1.13×10^{-2}
ENSRNOG00000002946	*Socs3*	5.11	7.97×10^{-8}
ENSRNOG00000054860	*Clec12a*	5.11	1.89×10^{-4}
ENSRNOG00000004757	*Tmem158*	5.11	1.54×10^{-5}
ENSRNOG00000008759	*Csf3r*	5.11	6.66×10^{-6}
ENSRNOG00000001224	*Itgb2*	5.08	6.53×10^{-7}
ENSRNOG00000006932	*AABR07029272.1*	5.07	1.79×10^{-5}
ENSRNOG00000036960	*Abcc9*	5.05	1.04×10^{-4}
ENSRNOG00000003136	*Fcrla*	5.04	1.82×10^{-2}
ENSRNOG00000032708	*RT1-Bb*	5.04	5.53×10^{-4}
ENSRNOG00000010918	*Cebpa*	5.03	2.00×10^{-5}
ENSRNOG00000006735	*Cdkn2b*	5.03	1.08×10^{-3}
ENSRNOG00000010079	*Car3*	5.02	4.75×10^{-4}
ENSRNOG00000019728	*Itgad*	5.02	6.25×10^{-10}
ENSRNOG00000018163	*Ipcef1*	5.02	8.92×10^{-5}
ENSRNOG00000014653	*Arl11*	5.01	1.68×10^{-4}
ENSRNOG00000038539	*Lrrc15*	4.99	9.54×10^{-5}
ENSRNOG00000009912	*Fgr*	4.98	4.67×10^{-6}
ENSRNOG00000000451	*RT1-Ba*	4.98	2.93×10^{-5}
ENSRNOG00000038916	*Dram1*	4.97	2.76×10^{-3}
ENSRNOG00000013578	*Trem2*	4.97	3.34×10^{-4}
ENSRNOG00000024382	*Fcgr3a*	4.97	6.88×10^{-14}
ENSRNOG00000008904	*Fli1*	4.96	4.12×10^{-7}
ENSRNOG00000021412	*Slfn13*	4.96	4.01×10^{-6}
ENSRNOG00000058906	*Ptprh*	4.95	8.51×10^{-5}
ENSRNOG00000030698	*Cyp26c1*	4.93	7.04×10^{-4}
ENSRNOG00000008586	*Aldh1l2*	4.93	4.14×10^{-4}
ENSRNOG00000004540	*Clec3b*	4.93	2.67×10^{-6}
ENSRNOG00000022769	*Sp100*	4.92	2.94×10^{-3}
ENSRNOG00000051986	*Plcg2*	4.92	1.09×10^{-4}
ENSRNOG00000019440	*Kcnn4*	4.92	1.64×10^{-3}
ENSRNOG00000015859	*Chdh*	4.91	1.59×10^{-5}
ENSRNOG00000012749	*C1qb*	4.91	5.85×10^{-12}
ENSRNOG00000006583	*Hpgds*	4.89	4.80×10^{-2}
ENSRNOG00000008824	*Vax1*	4.89	9.77×10^{-4}
ENSRNOG00000026605	*Ifi27l2b*	4.89	4.94×10^{-5}
ENSRNOG00000052695	*LOC102549869*	4.89	6.06×10^{-6}

ENSRNOG00000022426	*LOC102546864*	4.88	1.50×10^{-3}
ENSRNOG00000006946	*Arhgap9*	4.88	4.93×10^{-5}
ENSRNOG00000020716	*Axl*	4.87	2.64×10^{-6}
ENSRNOG00000003709	*Kmo*	4.87	6.06×10^{-3}
ENSRNOG00000037331	*Cd33*	4.86	1.49×10^{-7}
ENSRNOG00000014532	*Lbp*	4.84	3.00×10^{-2}
ENSRNOG00000010045	*Clec4a*	4.83	4.37×10^{-3}
ENSRNOG00000038881	*Hcls1*	4.83	8.72×10^{-7}
ENSRNOG00000021084	*AABR07006310.1*	4.83	1.37×10^{-7}
ENSRNOG00000004409	*Sash3*	4.82	3.96×10^{-5}
ENSRNOG00000018267	*B3gnt7*	4.81	2.60×10^{-5}
ENSRNOG00000005378	*Gna15*	4.80	9.78×10^{-6}
ENSRNOG00000009385	*Pik3cg*	4.79	9.63×10^{-7}
ENSRNOG00000037845	*Cysltr1*	4.79	9.67×10^{-3}
ENSRNOG00000038722	*Tlr1*	4.78	1.55×10^{-4}
ENSRNOG00000045595	*Tnfsf9*	4.78	2.46×10^{-2}
ENSRNOG00000024159	*Fcer1g*	4.78	7.52×10^{-8}
ENSRNOG00000046254	*Adgre1*	4.78	1.71×10^{-8}
ENSRNOG00000014253	*Pax2*	4.78	3.97×10^{-4}
ENSRNOG00000000991	*Arpc1b*	4.77	7.54×10^{-7}
ENSRNOG00000016978	*LOC308990*	4.77	5.96×10^{-3}
ENSRNOG00000012804	*C1qc*	4.75	1.92×10^{-10}
ENSRNOG00000002217	*Plac8*	4.75	1.01×10^{-10}
ENSRNOG00000011659	*Alpk3*	4.74	1.65×10^{-3}
ENSRNOG00000018915	*Tagap*	4.74	1.54×10^{-5}
ENSRNOG00000047606	*Bcl2a1*	4.74	5.15×10^{-4}
ENSRNOG00000026989	*Gapt*	4.72	3.07×10^{-3}
ENSRNOG00000058446	*Il7r*	4.72	6.78×10^{-3}
ENSRNOG00000018740	*Ugt1a1*	4.72	1.51×10^{-4}
ENSRNOG00000001704	*Runx1*	4.71	6.16×10^{-6}
ENSRNOG00000004192	*Arhgap30*	4.71	6.09×10^{-5}
ENSRNOG00000003720	*Prrx1*	4.71	1.67×10^{-3}
ENSRNOG00000019542	*LOC100910979*	4.70	4.28×10^{-2}
ENSRNOG00000029179	*Cyp2d3*	4.70	4.44×10^{-6}
ENSRNOG00000052357	*Fosl2*	4.70	1.99×10^{-5}
ENSRNOG00000049028	*C5ar2*	4.70	5.33×10^{-4}
ENSRNOG00000015297	*RGD1561662*	4.70	5.77×10^{-4}
ENSRNOG00000030729	*C4b*	4.69	5.38×10^{-4}
ENSRNOG00000008134	*Mefv*	4.69	2.50×10^{-6}
ENSRNOG00000005809	*Arhgdib*	4.69	3.12×10^{-9}
ENSRNOG00000017606	*P2rx1*	4.68	1.77×10^{-2}
ENSRNOG00000018005	*Duoxa1*	4.68	1.51×10^{-2}
ENSRNOG00000019270	*P2ry6*	4.66	1.02×10^{-8}
ENSRNOG00000011054	*Laptm5*	4.64	1.11×10^{-7}
ENSRNOG00000003895	*Rgs1*	4.64	1.63×10^{-2}
ENSRNOG00000018282	*Gda*	4.63	1.42×10^{-4}
ENSRNOG00000015616	*Rgs14*	4.63	2.71×10^{-2}
ENSRNOG00000059851	*AABR07070117.1*	4.62	2.03×10^{-2}
ENSRNOG00000042353	*Mlkl*	4.62	1.47×10^{-2}

ENSRNOG00000039496	*Plp2*	4.61	1.44×10^{-3}
ENSRNOG00000009347	*Arhgap25*	4.60	2.25×10^{-7}
ENSRNOG00000011381	*Acsbg1*	4.60	1.13×10^{-4}
ENSRNOG00000015002	*Abhd15*	4.59	5.22×10^{-4}
ENSRNOG00000020845	*Tyrobp*	4.59	2.93×10^{-5}
ENSRNOG00000001959	*Mx1*	4.59	4.70×10^{-10}
ENSRNOG00000036711	*Spn*	4.58	2.95×10^{-2}
ENSRNOG00000055962	*Bgn*	4.57	2.95×10^{-5}
ENSRNOG00000023546	*Hspb1*	4.57	7.55×10^{-10}
ENSRNOG00000029191	*Gbp7*	4.57	7.09×10^{-3}
ENSRNOG00000002968	*Tnfsf4*	4.57	1.18×10^{-2}
ENSRNOG00000004249	*Tlr7*	4.55	3.93×10^{-7}
ENSRNOG00000012172	*Spi1*	4.55	1.49×10^{-4}
ENSRNOG00000013231	*Ptafr*	4.54	2.93×10^{-5}
ENSRNOG00000037148	*Adap2*	4.48	2.21×10^{-3}
ENSRNOG00000000528	*Fgd2*	4.48	4.67×10^{-6}
ENSRNOG00000061403	*AABR07039446.2*	4.48	2.68×10^{-6}
ENSRNOG00000028566	*Pld4*	4.47	9.85×10^{-15}
ENSRNOG00000024000	*Cd22*	4.47	5.51×10^{-17}
ENSRNOG00000012807	*C1qa*	4.46	9.53×10^{-10}
ENSRNOG00000031743	*Gbp2*	4.45	6.94×10^{-8}
ENSRNOG00000017020	*Inpp5d*	4.45	3.23×10^{-7}
ENSRNOG00000001296	*P2rx7*	4.44	3.27×10^{-5}
ENSRNOG00000038835	*Cd86*	4.43	1.80×10^{-4}
ENSRNOG00000004725	*Cela1*	4.43	4.43×10^{-3}
ENSRNOG00000013720	*Aebp1*	4.42	1.71×10^{-5}
ENSRNOG00000016750	*Cyp26a1*	4.41	3.65×10^{-3}
ENSRNOG00000006940	*Ncf4*	4.41	2.84×10^{-4}
ENSRNOG00000001752	*Nrros*	4.41	6.88×10^{-5}
ENSRNOG00000006963	*Ctse*	4.40	2.10×10^{-4}
ENSRNOG00000033434	*Casp12*	4.40	5.12×10^{-3}
ENSRNOG00000010018	*Clec4a3*	4.40	2.60×10^{-3}
ENSRNOG00000010890	*Bmp1*	4.37	1.30×10^{-5}
ENSRNOG00000054218	*Il18rap*	4.37	2.16×10^{-2}
ENSRNOG00000016308	*Il10ra*	4.37	1.35×10^{-3}
ENSRNOG00000012331	*Casp8*	4.36	6.36×10^{-3}
ENSRNOG00000022884	*Cd84*	4.36	1.44×10^{-6}
ENSRNOG00000017063	*Fcna*	4.36	3.28×10^{-3}
ENSRNOG00000022537	*E2f8*	4.36	2.72×10^{-2}
ENSRNOG00000007081	*Xdh*	4.36	6.23×10^{-5}
ENSRNOG00000050697	*Ctsz*	4.35	4.38×10^{-6}
ENSRNOG00000022101	*Crabp2*	4.33	9.34×10^{-10}
ENSRNOG00000048186	*F2rl3*	4.33	2.30×10^{-2}
ENSRNOG00000002919	*Gfap*	4.33	4.98×10^{-11}
ENSRNOG00000015156	*Gal*	4.32	1.04×10^{-3}
ENSRNOG00000021345	*Timd2*	4.32	9.41×10^{-3}
ENSRNOG00000001544	*Cyyr1*	4.32	4.69×10^{-2}
ENSRNOG00000033444	*F10*	4.32	1.13×10^{-5}
ENSRNOG00000025198	*Gas2l3*	4.31	1.22×10^{-3}

ENSRNOG00000019851	*Cox6a2*	4.31	2.75 x 10^{-2}
ENSRNOG00000014327	*Csrp3*	4.31	9.21 x 10^{-4}
ENSRNOG00000031058	*Was*	4.31	9.63 x 10^{-5}
ENSRNOG00000007679	*Cyth4*	4.30	1.26 x 10^{-6}
ENSRNOG00000013102	*Entpd2*	4.29	6.20 x 10^{-3}
ENSRNOG00000033693	*AC135826.1*	4.29	7.99 x 10^{-4}
ENSRNOG00000010208	*Timp1*	4.28	4.95 x 10^{-7}
ENSRNOG00000009431	*Tbc1d4*	4.27	9.21 x 10^{-4}
ENSRNOG00000018488	*Cd40*	4.27	2.47 x 10^{-3}
ENSRNOG00000003018	*Olfml2b*	4.26	2.92 x 10^{-3}
ENSRNOG00000028016	*AABR07021402.1*	4.25	3.26 x 10^{-3}
ENSRNOG00000014956	*Slc11a1*	4.24	1.62 x 10^{-6}
ENSRNOG00000009471	*Epsti1*	4.24	1.01 x 10^{-5}
ENSRNOG00000042137	*Tmem173*	4.23	4.74 x 10^{-5}
ENSRNOG00000049743	*Gstm3*	4.22	9.82 x 10^{-3}
ENSRNOG00000031515	*Klra2*	4.22	1.61 x 10^{-5}
ENSRNOG00000004964	*Erbb3*	4.22	6.64 x 10^{-3}
ENSRNOG00000028896	*A2m*	4.22	1.69 x 10^{-5}
ENSRNOG00000010266	*Cd180*	4.21	2.83 x 10^{-7}
ENSRNOG00000060898	*LOC100910418*	4.21	3.01 x 10^{-2}
ENSRNOG00000036669	*Uts2r*	4.21	2.82 x 10^{-2}
ENSRNOG00000046834	*C3*	4.21	1.67 x 10^{-22}
ENSRNOG00000046452	*Fcgr2b*	4.20	7.44 x 10^{-8}
ENSRNOG00000000168	*Gatm*	4.19	5.13 x 10^{-6}
ENSRNOG00000010691	*Cmtm3*	4.19	1.83 x 10^{-7}
ENSRNOG00000046171	*AABR07035839.1*	4.19	2.25 x 10^{-7}
ENSRNOG00000004743	*Cdcp1*	4.19	3.49 x 10^{-2}
ENSRNOG00000014835	*Il1rl1*	4.18	1.13 x 10^{-5}
ENSRNOG00000002470	*Ifi47*	4.18	1.50 x 10^{-4}
ENSRNOG00000019838	*Gmfg*	4.16	4.72 x 10^{-4}
ENSRNOG00000004698	*Cd244*	4.15	1.42 x 10^{-2}
ENSRNOG00000003283	*Rcsd1*	4.15	4.97 x 10^{-3}
ENSRNOG00000003388	*Cenpf*	4.15	9.17 x 10^{-3}
ENSRNOG00000023143	*Nlrp1a*	4.13	1.73 x 10^{-3}
ENSRNOG00000005727	*Galnt3*	4.11	6.44 x 10^{-4}
ENSRNOG00000021856	*Lat2*	4.11	3.23 x 10^{-6}
ENSRNOG00000007258	*Sbspon*	4.10	8.14 x 10^{-3}
ENSRNOG00000015906	*Tgif1*	4.09	8.12 x 10^{-5}
ENSRNOG00000019283	*P2ry2*	4.09	6.21 x 10^{-4}
ENSRNOG00000007437	*Irf5*	4.09	9.37 x 10^{-6}
ENSRNOG00000003170	*Nlrp3*	4.08	7.57 x 10^{-5}
ENSRNOG00000046683	*Lilrb3*	4.07	5.94 x 10^{-5}
ENSRNOG00000006167	*Rasal3*	4.07	1.19 x 10^{-4}
ENSRNOG00000011718	*C1rl*	4.06	1.17 x 10^{-3}
ENSRNOG00000052407	*Btk*	4.05	1.90 x 10^{-3}
ENSRNOG00000011130	*Calca*	4.05	1.93 x 10^{-3}
ENSRNOG00000017208	*Cspg4*	4.04	3.77 x 10^{-4}
ENSRNOG00000019675	*Pycard*	4.04	2.70 x 10^{-4}
ENSRNOG00000007993	*Sh3tc1*	4.03	1.32 x 10^{-3}

ENSRNOG00000051235	*C2*	4.02	4.40×10^{-6}
ENSRNOG00000022975	*Nfam1*	4.02	5.42×10^{-5}
ENSRNOG00000002950	*Lyl1*	4.02	8.44×10^{-4}
ENSRNOG00000036834	*Gpr84*	4.01	2.38×10^{-10}
ENSRNOG00000030034	*Sox11*	4.01	1.58×10^{-3}
ENSRNOG00000005935	*A3galt2*	4.00	5.44×10^{-7}
ENSRNOG00000009848	*Il18*	4.00	7.99×10^{-4}
ENSRNOG00000008180	*Lyn*	4.00	1.54×10^{-5}
ENSRNOG00000016294	*Cd4*	3.99	6.51×10^{-8}
ENSRNOG00000031930	*Bin2*	3.98	3.43×10^{-9}
ENSRNOG00000049782	*Csf2ra*	3.97	2.03×10^{-3}
ENSRNOG00000032396	*RGD1559575*	3.97	8.85×10^{-3}
ENSRNOG00000014776	*Adcy7*	3.97	8.58×10^{-5}
ENSRNOG00000011647	*S100a6*	3.97	5.33×10^{-3}
ENSRNOG00000024365	*Ect2*	3.97	6.44×10^{-4}
ENSRNOG00000005620	*Lcp2*	3.96	1.46×10^{-4}
ENSRNOG00000014258	*Rab32*	3.95	1.51×10^{-4}
ENSRNOG00000000443	*LOC103689965*	3.94	1.09×10^{-4}
ENSRNOG00000011154	*Adgrf5*	3.94	1.63×10^{-2}
ENSRNOG00000010240	*Tent5a*	3.93	2.51×10^{-12}
ENSRNOG00000024277	*AABR07035470.1*	3.93	6.74×10^{-8}
ENSRNOG00000027229	*Slc35d2*	3.92	2.68×10^{-2}
ENSRNOG00000021474	*Siglec5*	3.91	5.79×10^{-3}
ENSRNOG00000017869	*Irf8*	3.91	3.76×10^{-6}
ENSRNOG00000027380	*Upk1b*	3.90	2.01×10^{-2}
ENSRNOG00000050215	*AABR07044375.1*	3.90	1.55×10^{-4}
ENSRNOG00000033940	*Adgrl4*	3.89	1.57×10^{-2}
ENSRNOG00000020811	*Il6r*	3.89	1.49×10^{-5}
ENSRNOG00000000525	*Pi16*	3.88	9.05×10^{-4}
ENSRNOG00000057522	*LOC103689968*	3.88	6.45×10^{-8}
ENSRNOG00000017693	*Slc2a5*	3.88	5.12×10^{-6}
ENSRNOG00000003972	*Tshr*	3.88	4.01×10^{-4}
ENSRNOG00000021031	*Grn*	3.87	3.73×10^{-5}
ENSRNOG00000028638	*Il10rb*	3.86	2.88×10^{-4}
ENSRNOG00000009157	*Fut4*	3.84	1.83×10^{-3}
ENSRNOG00000020884	*Cd6*	3.84	3.25×10^{-4}
ENSRNOG00000055111	*AABR07000658.1*	3.84	4.39×10^{-2}
ENSRNOG00000005580	*Itgb4*	3.84	3.82×10^{-4}
ENSRNOG00000003992	*Pik3r6*	3.83	9.66×10^{-3}
ENSRNOG00000010319	*Lcp1*	3.82	5.33×10^{-5}
ENSRNOG00000032311	*AABR07058658.1*	3.82	4.20×10^{-2}
ENSRNOG00000001249	*Col6a1*	3.82	2.69×10^{-2}
ENSRNOG00000012008	*S100a3*	3.81	2.58×10^{-11}
ENSRNOG00000061442	*AABR07068214.1*	3.80	4.90×10^{-2}
ENSRNOG00000017803	*Apbb1ip*	3.80	1.13×10^{-5}
ENSRNOG00000022189	*Htatip2*	3.79	7.83×10^{-3}
ENSRNOG00000053544	*AABR07070581.1*	3.78	2.07×10^{-3}
ENSRNOG00000050869	*Cebpd*	3.78	1.43×10^{-4}
ENSRNOG00000021713	*Kif18b*	3.78	4.25×10^{-2}

ENSRNOG00000036829	*Nckap1l*	3.77	9.41×10^{-7}
ENSRNOG00000023708	*Tmem176a*	3.77	8.26×10^{-7}
ENSRNOG00000010522	*Tlr4*	3.77	6.24×10^{-4}
ENSRNOG00000008622	*Creb5*	3.77	3.51×10^{-4}
ENSRNOG00000003310	*Tmem63a*	3.76	2.60×10^{-3}
ENSRNOG00000011153	*Il17ra*	3.76	2.59×10^{-5}
ENSRNOG00000011913	*Cp*	3.76	4.00×10^{-7}
ENSRNOG00000001130	*Nos1*	3.75	1.13×10^{-2}
ENSRNOG00000019179	*Ggta1*	3.74	1.13×10^{-4}
ENSRNOG00000001518	*Itga6*	3.74	3.94×10^{-6}
ENSRNOG00000000081	*Antxr2*	3.74	1.66×10^{-3}
ENSRNOG00000001908	*Klhl6*	3.73	1.46×10^{-4}
ENSRNOG00000053400	*Cfi*	3.72	8.33×10^{-3}
ENSRNOG00000054086	*Sp5*	3.71	1.46×10^{-2}
ENSRNOG00000005930	*Nnmt*	3.71	2.35×10^{-4}
ENSRNOG00000002218	*Stbd1*	3.70	6.25×10^{-7}
ENSRNOG00000012508	*Slc39a8*	3.70	1.76×10^{-3}
ENSRNOG00000007372	*Casp1*	3.68	1.70×10^{-3}
ENSRNOG00000015036	*Ccn2*	3.68	1.20×10^{-6}
ENSRNOG00000000137	*Ly86*	3.68	6.82×10^{-5}
ENSRNOG00000019141	*Ch25h*	3.68	1.93×10^{-5}
ENSRNOG00000000853	*Aif1*	3.68	1.18×10^{-5}
ENSRNOG00000002800	*Gdpd2*	3.67	8.93×10^{-6}
ENSRNOG00000015794	*Fam83d*	3.67	3.35×10^{-4}
ENSRNOG00000011821	*S100a4*	3.66	6.92×10^{-3}
ENSRNOG00000008676	*Emp1*	3.66	2.64×10^{-2}
ENSRNOG00000009260	*Cnr2*	3.66	4.78×10^{-3}
ENSRNOG00000012756	*Slc25a43*	3.66	2.82×10^{-3}
ENSRNOG00000055564	*RGD1564664*	3.65	4.14×10^{-4}
ENSRNOG00000047367	*Card14*	3.65	1.51×10^{-2}
ENSRNOG00000002045	*Anxa3*	3.64	8.15×10^{-23}
ENSRNOG00000011971	*C1s*	3.64	1.57×10^{-7}
ENSRNOG00000000454	*RT1-DOb*	3.63	4.61×10^{-3}
ENSRNOG00000018414	*Csf1r*	3.63	1.08×10^{-6}
ENSRNOG00000009822	*Tlr2*	3.63	1.37×10^{-4}
ENSRNOG00000005781	*Wnt16*	3.63	1.47×10^{-2}
ENSRNOG00000012067	*Fam111a*	3.62	3.91×10^{-2}
ENSRNOG00000016627	*Eya4*	3.62	5.60×10^{-3}
ENSRNOG00000043416	*Bcl3*	3.61	6.00×10^{-4}
ENSRNOG00000032778	*Bub1*	3.61	2.76×10^{-2}
ENSRNOG00000053260	*Lilrb3a*	3.60	1.11×10^{-13}
ENSRNOG00000008602	*Steap4*	3.60	6.00×10^{-3}
ENSRNOG00000011820	*Acpp*	3.60	7.38×10^{-5}
ENSRNOG00000059538	*Clec2g*	3.60	5.56×10^{-4}
ENSRNOG00000007415	*Ptgs1*	3.60	3.23×10^{-2}
ENSRNOG00000000569	*Vsir*	3.59	3.61×10^{-5}
ENSRNOG00000012730	*Lrrk1*	3.59	2.28×10^{-3}
ENSRNOG00000053272	*Chi3l1*	3.58	9.39×10^{-15}
ENSRNOG00000039754	*Rab7b*	3.58	2.12×10^{-4}

ENSRNOG00000023628	*Tmem106a*	3.57	9.05×10^{-4}
ENSRNOG00000003546	*Tnfrsf12a*	3.57	3.43×10^{-9}
ENSRNOG00000016846	*Pik3cd*	3.56	6.94×10^{-4}
ENSRNOG00000004367	*Elk3*	3.56	2.14×10^{-3}
ENSRNOG00000012628	*Gpr18*	3.56	2.05×10^{-2}
ENSRNOG00000042628	*Tmem273*	3.56	1.17×10^{-2}
ENSRNOG00000005695	*Mgp*	3.56	4.03×10^{-2}
ENSRNOG00000007060	*Plin2*	3.55	1.04×10^{-3}
ENSRNOG00000014294	*Ptpn6*	3.55	4.07×10^{-5}
ENSRNOG00000011947	*Tifab*	3.52	3.58×10^{-4}
ENSRNOG00000026965	*Tmem140*	3.52	2.54×10^{-3}
ENSRNOG00000007906	*Bub1b*	3.52	2.75×10^{-2}
ENSRNOG00000027030	*Adm*	3.52	1.34×10^{-3}
ENSRNOG00000016595	*Hhex*	3.52	3.51×10^{-2}
ENSRNOG00000020699	*Cd37*	3.51	3.68×10^{-6}
ENSRNOG00000032240	*Gbp5*	3.51	4.19×10^{-4}
ENSRNOG00000052981	*AABR07058464.1*	3.51	4.24×10^{-2}
ENSRNOG00000010866	*Tmem154*	3.51	2.37×10^{-2}
ENSRNOG00000011411	*Adgrg6*	3.50	7.82×10^{-4}
ENSRNOG00000002461	*Nid1*	3.50	1.35×10^{-3}
ENSRNOG00000022839	*Ifit3*	3.50	3.65×10^{-7}
ENSRNOG00000017828	*Egr3*	3.50	6.05×10^{-4}
ENSRNOG00000018659	*Csf1*	3.48	5.89×10^{-3}
ENSRNOG00000007457	*Serping1*	3.48	2.38×10^{-10}
ENSRNOG00000014867	*Synpo2*	3.47	8.58×10^{-3}
ENSRNOG00000037198	*Usp18*	3.47	4.73×10^{-5}
ENSRNOG00000000394	*Srgn*	3.46	7.28×10^{-5}
ENSRNOG00000016225	*Fgd3*	3.46	2.40×10^{-3}
ENSRNOG00000023400	*Dtx3l*	3.46	2.80×10^{-3}
ENSRNOG00000018509	*Cx3cr1*	3.46	1.99×10^{-8}
ENSRNOG00000001270	*Hvcn1*	3.46	1.14×10^{-2}
ENSRNOG00000002312	*Atp10d*	3.45	6.74×10^{-3}
ENSRNOG00000012843	*Aspg*	3.45	1.44×10^{-2}
ENSRNOG00000021100	*Tnfaip8l2*	3.44	1.04×10^{-3}
ENSRNOG00000000307	*Mical1*	3.44	2.88×10^{-4}
ENSRNOG00000037113	*Slfn2*	3.43	2.92×10^{-3}
ENSRNOG00000019128	*St8sia4*	3.43	3.16×10^{-3}
ENSRNOG00000016753	*Slc14a1*	3.43	1.71×10^{-4}
ENSRNOG00000026653	*Hcar2*	3.42	5.31×10^{-3}
ENSRNOG00000005214	*Plek*	3.42	7.28×10^{-6}
ENSRNOG00000017403	*Apobr*	3.42	9.18×10^{-3}
ENSRNOG00000026044	*Prrg1*	3.42	6.93×10^{-3}
ENSRNOG00000004063	*Sh3pxd2b*	3.41	4.83×10^{-3}
ENSRNOG00000013907	*Sall1*	3.41	4.84×10^{-8}
ENSRNOG00000010997	*Ednrb*	3.40	2.76×10^{-7}
ENSRNOG00000019854	*Napsa*	3.39	1.39×10^{-2}
ENSRNOG00000019202	*PVR*	3.39	2.62×10^{-7}
ENSRNOG00000051977	*Mmrn2*	3.38	4.37×10^{-3}
ENSRNOG00000058646	*Zfp36l1*	3.38	1.43×10^{-3}

ENSRNOG00000023334	*Parp14*	3.38	7.99×10^{-4}
ENSRNOG00000002134	*Gbp6*	3.38	2.92×10^{-3}
ENSRNOG00000018454	*Apoe*	3.37	9.67×10^{-3}
ENSRNOG00000021199	*Fcgr1a*	3.37	2.40×10^{-4}
ENSRNOG00000025332	*Cd109*	3.37	8.95×10^{-4}
ENSRNOG00000001804	*Itpr2*	3.37	3.03×10^{-4}
ENSRNOG00000053469	*AABR07030861.1*	3.36	4.52×10^{-3}
ENSRNOG00000000568	*Slc29a3*	3.36	7.26×10^{-4}
ENSRNOG00000018689	*RGD1305464*	3.36	1.01×10^{-3}
ENSRNOG00000016413	*Pstpip1*	3.36	2.76×10^{-2}
ENSRNOG00000029756	*P2ry13*	3.36	2.44×10^{-3}
ENSRNOG00000036604	*Ifit2*	3.35	2.20×10^{-5}
ENSRNOG00000042189	*Rab31*	3.35	1.17×10^{-3}
ENSRNOG00000001963	*Mx2*	3.35	3.47×10^{-8}
ENSRNOG00000037620	*Mob3c*	3.35	3.68×10^{-2}
ENSRNOG00000014227	*Mrgprx3*	3.34	3.32×10^{-2}
ENSRNOG00000007062	*Rin3*	3.33	1.22×10^{-2}
ENSRNOG00000019779	*Disc1*	3.33	4.48×10^{-3}
ENSRNOG00000031599	*Sh2d1b*	3.33	2.46×10^{-2}
ENSRNOG00000012318	*Aspm*	3.33	1.09×10^{-2}
ENSRNOG00000013265	*Tgfbr2*	3.33	3.81×10^{-3}
ENSRNOG00000005619	*Misp3*	3.33	1.77×10^{-2}
ENSRNOG00000002610	*Carhsp1*	3.33	3.34×10^{-4}
ENSRNOG00000028814	*Oasl2*	3.32	1.13×10^{-4}
ENSRNOG00000001607	*Adamts1*	3.32	1.48×10^{-2}
ENSRNOG00000033220	*Oas1k*	3.32	7.02×10^{-3}
ENSRNOG00000002664	*Emp2*	3.32	7.10×10^{-3}
ENSRNOG00000020117	*Brms1*	3.31	1.12×10^{-2}
ENSRNOG00000010275	*Slc17a9*	3.31	4.60×10^{-2}
ENSRNOG00000048449	*Itgb3*	3.31	1.51×10^{-3}
ENSRNOG00000053550	*Itga1*	3.30	1.49×10^{-4}
ENSRNOG00000016695	*Mmp2*	3.30	4.75×10^{-4}
ENSRNOG00000008736	*Slamf8*	3.30	5.89×10^{-3}
ENSRNOG00000017197	*Pdgfb*	3.29	2.27×10^{-3}
ENSRNOG00000021401	*Lpar5*	3.29	1.27×10^{-2}
ENSRNOG00000011796	*C1r*	3.27	7.97×10^{-4}
ENSRNOG00000020729	*Stc2*	3.27	3.21×10^{-2}
ENSRNOG00000014504	*Il1r1*	3.27	1.26×10^{-6}
ENSRNOG00000013014	*Cyba*	3.27	1.20×10^{-3}
ENSRNOG00000010263	*Cldn11*	3.27	7.96×10^{-5}
ENSRNOG00000010549	*Tspo*	3.27	5.86×10^{-6}
ENSRNOG00000017307	*Prss23*	3.26	9.77×10^{-4}
ENSRNOG00000010296	*Slc7a7*	3.25	1.58×10^{-3}
ENSRNOG00000030118	*Msn*	3.24	8.67×10^{-7}
ENSRNOG00000009222	*Epha2*	3.24	1.41×10^{-2}
ENSRNOG00000014870	*Slc13a5*	3.24	9.93×10^{-4}
ENSRNOG00000005575	*Abi3*	3.23	6.96×10^{-5}
ENSRNOG00000013572	*Lxn*	3.23	3.77×10^{-4}
ENSRNOG00000042771	*Apol3*	3.23	1.83×10^{-2}

ENSRNOG00000024818	*Eva1b*	3.23	1.21×10^{-3}
ENSRNOG00000048222	*Nlrc5*	3.22	9.59×10^{-3}
ENSRNOG00000002540	*Rhoh*	3.22	3.02×10^{-2}
ENSRNOG00000033747	*Sp110*	3.22	2.28×10^{-3}
ENSRNOG00000014125	*Evi2b*	3.22	5.02×10^{-4}
ENSRNOG00000003120	*Prelp*	3.22	2.81×10^{-3}
ENSRNOG00000046050	*Dennd1c*	3.21	3.58×10^{-2}
ENSRNOG00000017146	*Nfatc1*	3.21	7.12×10^{-3}
ENSRNOG00000010630	*Prcp*	3.21	7.20×10^{-4}
ENSRNOG00000045772	*LOC100911545*	3.21	2.83×10^{-4}
ENSRNOG00000025476	*Tmem252*	3.21	1.75×10^{-2}
ENSRNOG00000053200	*AABR07068161.1*	3.21	2.92×10^{-4}
ENSRNOG00000020349	*Rab3il1*	3.21	1.75×10^{-3}
ENSRNOG00000001425	*Sh2b2*	3.21	3.58×10^{-3}
ENSRNOG00000000824	*Dse*	3.20	5.76×10^{-3}
ENSRNOG00000008115	*Arhgap11a*	3.20	3.02×10^{-3}
ENSRNOG00000039255	*Gpr31*	3.19	8.04×10^{-4}
ENSRNOG00000004500	*Myc*	3.19	9.99×10^{-4}
ENSRNOG00000049598	*LOC100912571*	3.19	3.64×10^{-2}
ENSRNOG00000021261	*Rassf2*	3.18	9.00×10^{-4}
ENSRNOG00000049552	*AABR07030903.1*	3.18	3.76×10^{-2}
ENSRNOG00000004444	*Ikzf1*	3.18	9.63×10^{-5}
ENSRNOG00000010626	*Sphk1*	3.17	1.13×10^{-3}
ENSRNOG00000013674	*Megf10*	3.17	2.40×10^{-3}
ENSRNOG00000060829	*AC125248.1*	3.16	1.79×10^{-5}
ENSRNOG00000001369	*Oas1a*	3.16	2.59×10^{-4}
ENSRNOG00000013564	*Dok3*	3.16	8.47×10^{-3}
ENSRNOG00000009433	*Mcub*	3.16	1.35×10^{-3}
ENSRNOG00000045771	*Chl1*	3.16	3.79×10^{-3}
ENSRNOG00000023463	*Parp9*	3.15	6.68×10^{-4}
ENSRNOG00000007477	*Edn3*	3.15	2.32×10^{-2}
ENSRNOG00000004226	*Irak3*	3.14	4.11×10^{-10}
ENSRNOG00000056714	*Sla*	3.14	1.54×10^{-3}
ENSRNOG00000039336	*Hrct1*	3.14	1.82×10^{-2}
ENSRNOG00000013387	*Tpcn2*	3.14	3.30×10^{-2}
ENSRNOG00000018406	*Wipf1*	3.14	2.36×10^{-3}
ENSRNOG00000017556	*Chrm4*	3.13	1.13×10^{-2}
ENSRNOG00000033984	*Ifnlr1*	3.12	3.77×10^{-6}
ENSRNOG00000016617	*Wwtr1*	3.12	1.03×10^{-2}
ENSRNOG00000015894	*Dock8*	3.12	2.38×10^{-3}
ENSRNOG00000042741	*Adgb*	3.11	1.39×10^{-2}
ENSRNOG00000002161	*Tlr6*	3.11	5.78×10^{-3}
ENSRNOG00000051952	*Tes*	3.10	2.44×10^{-6}
ENSRNOG00000031004	*Cyp2j4*	3.10	1.31×10^{-2}
ENSRNOG00000024230	*Tnfaip8l3*	3.10	1.72×10^{-3}
ENSRNOG00000014797	*Tmbim1*	3.10	3.05×10^{-4}
ENSRNOG00000029682	*Clic1*	3.10	4.35×10^{-8}
ENSRNOG00000011407	*Prag1*	3.10	2.05×10^{-3}
ENSRNOG00000019430	*Coro1a*	3.09	7.97×10^{-6}

ENSRNOG00000047714	*Tmem37*	3.09	4.87×10^{-2}
ENSRNOG00000031312	*Tnfrsf1a*	3.09	9.41×10^{-3}
ENSRNOG00000019659	*Aspa*	3.08	3.00×10^{-4}
ENSRNOG00000016242	*Fzd1*	3.08	4.10×10^{-3}
ENSRNOG00000048462	*AABR07066529.1*	3.07	4.73×10^{-2}
ENSRNOG00000011781	*Oplah*	3.07	2.66×10^{-3}
ENSRNOG00000002159	*Gpat3*	3.07	9.31×10^{-4}
ENSRNOG00000024346	*Plet1*	3.06	1.27×10^{-2}
ENSRNOG00000017976	*Slco2b1*	3.06	2.75×10^{-5}
ENSRNOG00000033433	*Csrnp1*	3.06	3.17×10^{-7}
ENSRNOG00000061630	*AABR07009834.1*	3.06	9.04×10^{-3}
ENSRNOG00000001250	*Lfng*	3.06	1.46×10^{-2}
ENSRNOG00000027309	*AABR07026805.1*	3.06	3.14×10^{-2}
ENSRNOG00000028266	*Lrrc55*	3.06	4.63×10^{-3}
ENSRNOG00000019403	*Afap1l1*	3.05	4.40×10^{-2}
ENSRNOG00000014259	*Mycl*	3.05	4.05×10^{-3}
ENSRNOG00000001807	*Sspn*	3.05	1.29×10^{-2}
ENSRNOG00000014398	*Scara5*	3.04	9.99×10^{-4}
ENSRNOG00000006079	*Psd4*	3.04	5.30×10^{-3}
ENSRNOG00000008465	*Tmem176b*	3.04	8.58×10^{-5}
ENSRNOG00000024294	*AABR07019083.1*	3.03	8.74×10^{-4}
ENSRNOG00000021157	*Ctss*	3.03	2.25×10^{-6}
ENSRNOG00000007726	*Mcam*	3.03	9.99×10^{-4}
ENSRNOG00000008026	*C2cd4c*	3.03	9.86×10^{-3}
ENSRNOG00000032871	*Mlc1*	3.03	3.21×10^{-7}
ENSRNOG00000005302	*Slc2a9*	3.03	4.37×10^{-2}
ENSRNOG00000022721	*Clec18a*	3.02	1.11×10^{-2}
ENSRNOG00000025619	*Ap1g2*	3.02	2.57×10^{-3}
ENSRNOG00000050792	*Tnfaip6*	3.02	2.64×10^{-3}
ENSRNOG00000020684	*Vat1*	3.01	9.83×10^{-4}
ENSRNOG00000004332	*Egfr*	3.01	1.41×10^{-2}
ENSRNOG00000020942	*Plekha4*	3.01	5.84×10^{-9}
ENSRNOG00000020281	*Kif22*	3.00	9.38×10^{-5}
ENSRNOG00000008587	*Tek*	3.00	4.12×10^{-2}
ENSRNOG00000018400	*Golm1*	2.99	6.53×10^{-4}
ENSRNOG00000000459	*Psmb9*	2.99	1.57×10^{-2}
ENSRNOG00000018384	*Adam12*	2.99	3.51×10^{-2}
ENSRNOG00000028569	*Arhgap27*	2.99	1.16×10^{-2}
ENSRNOG00000027990	*Crip1*	2.99	3.65×10^{-2}
ENSRNOG00000024207	*Fgfrl1*	2.98	9.38×10^{-4}
ENSRNOG00000020505	*Map4k1*	2.98	1.70×10^{-2}
ENSRNOG00000006952	*Prex1*	2.98	3.02×10^{-4}
ENSRNOG00000009369	*Tor4a*	2.98	1.64×10^{-2}
ENSRNOG00000014055	*Nat1*	2.97	6.28×10^{-3}
ENSRNOG00000014205	*Klf2*	2.96	4.80×10^{-3}
ENSRNOG00000020583	*Fcgrt*	2.96	6.51×10^{-3}
ENSRNOG00000016037	*Mafb*	2.95	1.36×10^{-2}
ENSRNOG00000014336	*Mcm5*	2.95	1.39×10^{-2}
ENSRNOG00000006030	*Ptprz1*	2.95	6.70×10^{-4}

ENSRNOG00000024728	*Arhgap22*	2.94	2.88×10^{-4}
ENSRNOG00000012228	*Skap2*	2.94	1.15×10^{-3}
ENSRNOG00000022764	*Evi2a*	2.94	7.97×10^{-6}
ENSRNOG00000016581	*Serpinb1a*	2.94	1.13×10^{-3}
ENSRNOG00000034134	*Cpm*	2.93	4.31×10^{-3}
ENSRNOG00000014064	*Ctsh*	2.93	1.97×10^{-4}
ENSRNOG00000001375	*Gal3st4*	2.93	2.88×10^{-4}
ENSRNOG00000007743	*Mgst1*	2.92	6.22×10^{-3}
ENSRNOG00000009785	*Cdkn3*	2.91	1.69×10^{-2}
ENSRNOG00000008012	*Abcb4*	2.91	5.08×10^{-3}
ENSRNOG00000002607	*Sox9*	2.91	6.06×10^{-3}
ENSRNOG00000031443	*Havcr2*	2.90	1.01×10^{-4}
ENSRNOG00000057125	*Ddr1*	2.90	3.29×10^{-3}
ENSRNOG00000002413	*Gpc4*	2.89	8.50×10^{-3}
ENSRNOG00000011517	*Tnfrsf21*	2.89	7.37×10^{-3}
ENSRNOG00000021663	*Vxn*	2.89	2.07×10^{-2}
ENSRNOG00000003104	*Trpv2*	2.89	1.72×10^{-3}
ENSRNOG00000010362	*Anxa2*	2.88	5.71×10^{-5}
ENSRNOG00000022094	*Lacc1*	2.88	4.97×10^{-2}
ENSRNOG00000017093	*Pxdc1*	2.88	7.03×10^{-3}
ENSRNOG00000031540	*LOC100362384*	2.88	3.51×10^{-2}
ENSRNOG00000018669	*Jak3*	2.87	9.38×10^{-5}
ENSRNOG00000018911	*Pfkfb3*	2.87	6.41×10^{-4}
ENSRNOG00000010966	*Itgb1*	2.87	2.39×10^{-3}
ENSRNOG00000017277	*Igsf6*	2.87	4.66×10^{-2}
ENSRNOG00000016460	*Clu*	2.87	1.08×10^{-4}
ENSRNOG00000024846	*Ier5l*	2.87	1.50×10^{-2}
ENSRNOG00000014838	*Glipr2*	2.86	1.44×10^{-4}
ENSRNOG00000019556	*Cd9*	2.86	2.26×10^{-3}
ENSRNOG00000043044	*Cnn2*	2.86	3.87×10^{-3}
ENSRNOG00000008182	*Htra3*	2.86	4.99×10^{-2}
ENSRNOG00000016346	*Prkcd*	2.86	6.27×10^{-3}
ENSRNOG00000013902	*P2ry12*	2.86	3.83×10^{-7}
ENSRNOG00000022800	*Sp140*	2.86	2.69×10^{-3}
ENSRNOG00000046848	*PCOLCE2*	2.85	9.55×10^{-3}
ENSRNOG00000019651	*Slc12a4*	2.85	8.45×10^{-3}
ENSRNOG00000017369	*Mustn1*	2.85	2.07×10^{-2}
ENSRNOG00000024899	*Cxcl13*	2.85	3.62×10^{-4}
ENSRNOG00000053339	*AABR07062512.1*	2.85	3.22×10^{-3}
ENSRNOG00000029586	*AC128059.1*	2.84	4.64×10^{-2}
ENSRNOG00000020486	*Bcl2l12*	2.84	2.78×10^{-2}
ENSRNOG00000012062	*Npc2*	2.84	1.54×10^{-3}
ENSRNOG00000016687	*Ssc5d*	2.84	6.40×10^{-3}
ENSRNOG00000022760	*Rubcnl*	2.84	2.00×10^{-2}
ENSRNOG00000012811	*Spint1*	2.83	1.79×10^{-4}
ENSRNOG00000007041	*Abcg2*	2.83	2.08×10^{-3}
ENSRNOG00000029841	*Cdh19*	2.83	8.07×10^{-9}
ENSRNOG00000008941	*Ets1*	2.82	1.76×10^{-3}
ENSRNOG00000009258	*Cdk6*	2.82	6.93×10^{-3}

ENSRNOG00000010775	*Arrdc4*	2.82	1.13×10^{-3}
ENSRNOG00000012681	*Lgals9*	2.81	1.77×10^{-2}
ENSRNOG00000001314	*Fam20c*	2.81	2.58×10^{-3}
ENSRNOG00000036918	*Etfbkmt*	2.81	5.87×10^{-3}
ENSRNOG00000005275	*Shmt1*	2.80	2.04×10^{-2}
ENSRNOG00000028895	*Rtp4*	2.80	1.11×10^{-2}
ENSRNOG00000004489	*Adgre5*	2.80	4.95×10^{-3}
ENSRNOG00000013526	*Rassf4*	2.80	3.22×10^{-3}
ENSRNOG00000018159	*Anxa4*	2.79	1.68×10^{-2}
ENSRNOG00000009144	*Lad1*	2.79	1.66×10^{-4}
ENSRNOG00000014963	*Adgrg1*	2.79	1.28×10^{-2}
ENSRNOG00000057569	*Ahnak*	2.78	2.00×10^{-2}
ENSRNOG00000017212	*Spsb1*	2.78	4.29×10^{-3}
ENSRNOG00000005965	*Irak4*	2.77	2.67×10^{-3}
ENSRNOG00000059900	*Bst2*	2.77	1.16×10^{-2}
ENSRNOG00000029658	*Rnf213*	2.77	1.71×10^{-3}
ENSRNOG00000020726	*Sipa1*	2.77	4.00×10^{-3}
ENSRNOG00000002122	*Lrrc8c*	2.77	1.62×10^{-2}
ENSRNOG00000043286	*Cpne2*	2.75	5.90×10^{-4}
ENSRNOG00000012906	*Bcas1*	2.75	2.70×10^{-2}
ENSRNOG00000057556	*Pdzrn3*	2.74	1.68×10^{-3}
ENSRNOG00000004133	*Slc7a3*	2.74	4.70×10^{-10}
ENSRNOG00000061768	*Slc43a3*	2.74	4.70×10^{-10}
ENSRNOG00000012428	*Maf*	2.74	5.75×10^{-3}
ENSRNOG00000003088	*Arhgap31*	2.73	4.35×10^{-3}
ENSRNOG00000003430	*F9*	2.73	1.80×10^{-2}
ENSRNOG00000004874	*Flrt3*	2.72	6.57×10^{-3}
ENSRNOG00000000700	*Tmem119*	2.72	9.05×10^{-4}
ENSRNOG00000012946	*Mov10*	2.72	2.33×10^{-2}
ENSRNOG00000013967	*Blnk*	2.72	9.06×10^{-3}
ENSRNOG00000053026	*Shcbp1*	2.72	1.14×10^{-2}
ENSRNOG00000020679	*Icam1*	2.71	1.92×10^{-2}
ENSRNOG00000042224	*Cyp2j10*	2.71	9.21×10^{-4}
ENSRNOG00000000457	*Tap1*	2.71	6.68×10^{-4}
ENSRNOG00000004217	*Stk10*	2.71	1.21×10^{-2}
ENSRNOG00000021020	*Gpha2*	2.71	1.55×10^{-2}
ENSRNOG00000017164	*Afap1l2*	2.71	4.51×10^{-2}
ENSRNOG00000018770	*Pmaip1*	2.70	4.72×10^{-2}
ENSRNOG00000000869	*Arhgef6*	2.69	1.51×10^{-2}
ENSRNOG00000021104	*Emp3*	2.69	1.69×10^{-2}
ENSRNOG00000058388	*Zfp36*	2.68	2.38×10^{-3}
ENSRNOG00000023778	*Gcnt2*	2.68	1.18×10^{-2}
ENSRNOG00000006548	*Mrc2*	2.68	1.79×10^{-2}
ENSRNOG00000055650	*Pou2f2*	2.68	6.42×10^{-3}
ENSRNOG00000014961	*Pdpn*	2.68	6.00×10^{-4}
ENSRNOG00000017123	*B2m*	2.67	1.91×10^{-7}
ENSRNOG00000019892	*Lrrfip1*	2.67	8.45×10^{-3}
ENSRNOG00000019357	*Tax1bp3*	2.67	6.68×10^{-4}
ENSRNOG00000020843	*Ftl1*	2.67	1.16×10^{-2}

ENSRNOG00000013215	*Dctd*	2.67	7.82×10^{-4}
ENSRNOG00000000561	*Pald1*	2.67	1.05×10^{-2}
ENSRNOG00000031475	*Col16a1*	2.67	8.02×10^{-3}
ENSRNOG00000002680	*Lamc1*	2.67	1.51×10^{-2}
ENSRNOG00000028801	*Gsap*	2.67	2.97×10^{-2}
ENSRNOG00000005807	*Ptpn7*	2.67	9.11×10^{-4}
ENSRNOG00000016378	*Map3k8*	2.66	3.74×10^{-2}
ENSRNOG00000047447	*AC115420.2*	2.66	1.44×10^{-2}
ENSRNOG00000040205	*Zcchc24*	2.66	7.84×10^{-4}
ENSRNOG00000039759	*Gpr34*	2.66	1.13×10^{-5}
ENSRNOG00000019018	*Plat*	2.66	8.70×10^{-3}
ENSRNOG00000007483	*Ccnf*	2.65	1.99×10^{-4}
ENSRNOG00000017319	*Mertk*	2.65	1.26×10^{-2}
ENSRNOG00000052219	*Gm2a*	2.65	4.15×10^{-3}
ENSRNOG00000020653	*S1pr2*	2.65	1.05×10^{-2}
ENSRNOG00000002050	*Igfbp7*	2.65	8.49×10^{-3}
ENSRNOG00000050877	*LOC100912538*	2.64	8.33×10^{-3}
ENSRNOG00000010643	*Kank2*	2.64	1.16×10^{-2}
ENSRNOG00000007728	*Gsdmd*	2.64	4.87×10^{-3}
ENSRNOG00000004330	*Chrdl1*	2.63	1.57×10^{-2}
ENSRNOG00000049614	*AABR07028488.1*	2.63	6.79×10^{-3}
ENSRNOG00000031643	*Dchs1*	2.63	2.52×10^{-2}
ENSRNOG00000000699	*Selplg*	2.63	4.42×10^{-5}
ENSRNOG00000003338	*Pmp22*	2.62	1.21×10^{-2}
ENSRNOG00000015382	*Arid5a*	2.62	1.71×10^{-8}
ENSRNOG00000000321	*Cd24*	2.62	3.43×10^{-9}
ENSRNOG00000043366	*AABR07063829.2*	2.62	1.05×10^{-3}
ENSRNOG00000021787	*Zfp217*	2.62	1.85×10^{-2}
ENSRNOG00000005650	*Pgf*	2.61	1.15×10^{-2}
ENSRNOG00000023410	*Apol9a*	2.61	2.73×10^{-2}
ENSRNOG00000016547	*Rgs19*	2.61	4.83×10^{-3}
ENSRNOG00000025371	*Spry1*	2.61	6.51×10^{-3}
ENSRNOG00000024566	*Sh3d21*	2.61	3.87×10^{-2}
ENSRNOG00000050231	*Gng12*	2.60	1.00×10^{-2}
ENSRNOG00000060665	*Afap1*	2.60	2.07×10^{-3}
ENSRNOG00000060329	*Emb*	2.59	3.11×10^{-2}
ENSRNOG00000013663	*Tmem86a*	2.59	1.21×10^{-2}
ENSRNOG00000021170	*Plekho1*	2.59	3.23×10^{-7}
ENSRNOG00000020657	*Shc1*	2.59	5.76×10^{-3}
ENSRNOG00000020393	*Rhog*	2.58	6.90×10^{-3}
ENSRNOG00000014524	*S1pr3*	2.58	5.47×10^{-3}
ENSRNOG00000010747	*Dap*	2.58	1.79×10^{-2}
ENSRNOG00000020813	*Ltbp3*	2.58	1.34×10^{-2}
ENSRNOG00000019129	*Fcgbp*	2.57	3.27×10^{-2}
ENSRNOG00000009113	*Marcksl1*	2.57	5.42×10^{-5}
ENSRNOG00000043098	*Mt2A*	2.57	3.42×10^{-3}
ENSRNOG00000009219	*Hepacam*	2.57	2.42×10^{-2}
ENSRNOG00000008517	*Cdc42ep1*	2.57	1.49×10^{-2}
ENSRNOG00000001300	*P2rx4*	2.57	1.19×10^{-2}

ENSRNOG00000050547	*Syngr2*	2.56	1.90×10^{-2}
ENSRNOG00000052444	*Samd9*	2.56	3.28×10^{-4}
ENSRNOG00000023830	*Dnase2*	2.56	1.28×10^{-2}
ENSRNOG00000053766	*Ramp3*	2.56	3.39×10^{-2}
ENSRNOG00000016163	*Slc1a3*	2.56	8.89×10^{-4}
ENSRNOG00000010575	*Dapp1*	2.56	1.35×10^{-3}
ENSRNOG00000020182	*Mvp*	2.55	1.05×10^{-2}
ENSRNOG00000051706	*Tep1*	2.55	3.80×10^{-3}
ENSRNOG00000015812	*Tm4sf1*	2.55	2.12×10^{-2}
ENSRNOG00000013321	*Dock11*	2.55	2.11×10^{-3}
ENSRNOG00000017087	*Man1c1*	2.55	1.41×10^{-2}
ENSRNOG00000019264	*Ltbr*	2.55	6.36×10^{-3}
ENSRNOG00000006472	*Hspa2*	2.54	9.66×10^{-3}
ENSRNOG00000019203	*Eya2*	2.54	8.33×10^{-3}
ENSRNOG00000020667	*Shisa5*	2.54	5.30×10^{-3}
ENSRNOG00000014350	*Ccn1*	2.54	1.22×10^{-2}
ENSRNOG00000002657	*Pla2g4a*	2.54	1.12×10^{-2}
ENSRNOG00000037371	*Xaf1*	2.53	2.25×10^{-3}
ENSRNOG00000007539	*Rsad2*	2.53	3.23×10^{-6}
ENSRNOG00000000024	*Hebp1*	2.53	2.05×10^{-2}
ENSRNOG00000009951	*Aif1l*	2.53	6.98×10^{-3}
ENSRNOG00000013309	*Pik3ap1*	2.53	4.72×10^{-3}
ENSRNOG00000017191	*Trim5*	2.53	2.10×10^{-2}
ENSRNOG00000006324	*Trpc6*	2.53	2.28×10^{-3}
ENSRNOG00000019080	*Hsd3b7*	2.53	1.78×10^{-5}
ENSRNOG00000012422	*Tnik*	2.53	9.64×10^{-3}
ENSRNOG00000005956	*Erich5*	2.52	2.08×10^{-3}
ENSRNOG00000042686	*Trim34*	2.52	2.25×10^{-2}
ENSRNOG00000025676	*Gask1a*	2.52	4.23×10^{-2}
ENSRNOG00000015717	*Ptpre*	2.52	1.77×10^{-2}
ENSRNOG00000017414	*Irf7*	2.51	4.63×10^{-4}
ENSRNOG00000016391	*Arid4b*	2.51	1.62×10^{-2}
ENSRNOG00000026748	*Dennd2a*	2.50	6.79×10^{-3}
ENSRNOG00000053691	*Lama5*	2.50	4.14×10^{-2}
ENSRNOG00000000907	*Alox5ap*	2.50	7.91×10^{-3}
ENSRNOG00000004448	*Acss3*	2.50	1.11×10^{-2}
ENSRNOG00000019638	*Lmna*	2.50	7.38×10^{-4}
ENSRNOG00000059463	*Slc39a1*	2.49	2.71×10^{-3}
ENSRNOG00000031138	*Irgm*	2.48	9.66×10^{-3}
ENSRNOG00000012199	*Sox2*	2.48	5.89×10^{-3}
ENSRNOG00000000485	*Bak1*	2.48	2.07×10^{-2}
ENSRNOG00000001192	*Gltp*	2.48	4.58×10^{-3}
ENSRNOG00000012865	*Parp3*	2.48	1.26×10^{-3}
ENSRNOG00000025811	*Cfp*	2.48	2.34×10^{-2}
ENSRNOG00000023781	*Plec*	2.47	1.06×10^{-2}
ENSRNOG00000021802	*Isg15*	2.47	1.80×10^{-3}
ENSRNOG00000000632	*Cdk1*	2.46	3.52×10^{-2}
ENSRNOG00000038891	*Grxcr1*	2.46	1.97×10^{-2}
ENSRNOG00000012966	*Acadl*	2.46	5.97×10^{-3}

ENSRNOG00000020594	*Rhbdf1*	2.46	2.59×10^{-2}
ENSRNOG00000061524	*AABR07068285.2*	2.46	9.61×10^{-3}
ENSRNOG00000019077	*Lipa*	2.46	2.16×10^{-2}
ENSRNOG00000016957	*Igfbp2*	2.46	7.97×10^{-4}
ENSRNOG00000002227	*Kit*	2.46	4.35×10^{-3}
ENSRNOG00000027008	*Igtp*	2.46	4.80×10^{-3}
ENSRNOG00000018369	*Prx*	2.45	4.66×10^{-2}
ENSRNOG00000030183	*Plod2*	2.45	2.81×10^{-2}
ENSRNOG00000021151	*Ppp1r14b*	2.45	5.33×10^{-4}
ENSRNOG00000056248	*Marf1*	2.44	7.37×10^{-4}
ENSRNOG00000047768	*Lamb2*	2.44	2.51×10^{-2}
ENSRNOG00000013683	*S1pr1*	2.44	3.79×10^{-2}
ENSRNOG00000008937	*Csrp1*	2.43	1.56×10^{-2}
ENSRNOG00000013090	*Gadd45g*	2.43	1.42×10^{-9}
ENSRNOG00000001271	*Card6*	2.43	1.42×10^{-2}
ENSRNOG00000028198	*Sh2b3*	2.43	9.78×10^{-3}
ENSRNOG00000000529	*Pim1*	2.42	1.01×10^{-10}
ENSRNOG00000032922	*Dclk1*	2.42	5.03×10^{-3}
ENSRNOG00000005041	*Crip2*	2.42	1.85×10^{-2}
ENSRNOG00000014832	*Mapkapk3*	2.41	4.40×10^{-3}
ENSRNOG00000020298	*Bag3*	2.41	3.69×10^{-2}
ENSRNOG00000012439	*Bid*	2.41	3.67×10^{-3}
ENSRNOG00000020205	*Agrn*	2.41	2.66×10^{-2}
ENSRNOG00000013092	*Lonrf3*	2.40	2.24×10^{-5}
ENSRNOG00000003172	*Serpinf1*	2.40	6.92×10^{-3}
ENSRNOG00000008015	*Fos*	2.39	1.06×10^{-2}
ENSRNOG00000017259	*Tacc3*	2.38	9.72×10^{-3}
ENSRNOG00000009530	*Uchl3*	2.38	2.63×10^{-2}
ENSRNOG00000026504	*Fam114a1*	2.38	1.74×10^{-2}
ENSRNOG00000057347	*Cebpb*	2.38	1.60×10^{-2}
ENSRNOG00000030715	*Cfh*	2.38	1.55×10^{-2}
ENSRNOG00000004111	*Soat1*	2.37	3.06×10^{-2}
ENSRNOG00000061237	*Dio1*	2.37	4.87×10^{-2}
ENSRNOG00000027502	*Nmi*	2.37	1.14×10^{-2}
ENSRNOG00000017595	*Rsu1*	2.37	1.61×10^{-2}
ENSRNOG00000062252	*AABR07072853.4*	2.36	7.52×10^{-3}
ENSRNOG00000010663	*Col6a5*	2.36	3.97×10^{-4}
ENSRNOG00000006384	*Ddx58*	2.36	2.16×10^{-2}
ENSRNOG00000046829	*Kdr*	2.36	3.28×10^{-4}
ENSRNOG00000006995	*Ano6*	2.35	3.07×10^{-2}
ENSRNOG00000047734	*Chst2*	2.35	6.24×10^{-3}
ENSRNOG00000007541	*Fhl3*	2.35	1.94×10^{-2}
ENSRNOG00000049115	*Ccr5*	2.35	3.22×10^{-6}
ENSRNOG00000047706	*LOC103690108*	2.34	1.42×10^{-2}
ENSRNOG00000012460	*Cntf*	2.34	6.37×10^{-3}
ENSRNOG00000007939	*Naprt*	2.34	1.31×10^{-2}
ENSRNOG00000003244	*Ltc4s*	2.34	5.49×10^{-3}
ENSRNOG00000026647	*Cxcl16*	2.34	2.86×10^{-2}
ENSRNOG00000006738	*Fbxo32*	2.34	2.35×10^{-4}

ENSRNOG00000010107	*AABR07025295.1*	2.34	2.81×10^{-2}
ENSRNOG00000009360	*Sh3bp1*	2.33	6.36×10^{-3}
ENSRNOG00000011417	*Pde3b*	2.33	6.37×10^{-3}
ENSRNOG00000019244	*Mxra8*	2.33	2.19×10^{-2}
ENSRNOG00000016852	*Apobec3*	2.33	4.53×10^{-2}
ENSRNOG00000014783	*Pgghg*	2.33	2.95×10^{-2}
ENSRNOG00000016071	*P3h3*	2.33	1.11×10^{-2}
ENSRNOG00000013481	*Cdh11*	2.32	3.99×10^{-2}
ENSRNOG00000039890	*Abcg3l3*	2.32	4.24×10^{-2}
ENSRNOG00000011373	*Reep4*	2.32	6.05×10^{-3}
ENSRNOG00000003645	*Hnrnpab*	2.32	2.63×10^{-2}
ENSRNOG00000008016	*Ckap4*	2.32	4.43×10^{-3}
ENSRNOG00000028368	*Etnk2*	2.31	1.48×10^{-2}
ENSRNOG00000054625	*Fhod1*	2.31	4.32×10^{-2}
ENSRNOG00000032327	*Pdia5*	2.31	4.58×10^{-2}
ENSRNOG00000029865	*Prss56*	2.31	2.46×10^{-2}
ENSRNOG00000008965	*Socs2*	2.31	2.82×10^{-2}
ENSRNOG00000005903	*St14*	2.30	1.35×10^{-2}
ENSRNOG00000016289	*Bmpr1b*	2.30	2.57×10^{-2}
ENSRNOG00000018247	*Dhx58*	2.29	6.36×10^{-3}
ENSRNOG00000002520	*Litaf*	2.29	1.02×10^{-2}
ENSRNOG00000010196	*Glb1*	2.29	1.51×10^{-2}
ENSRNOG00000037247	*Rras*	2.28	1.39×10^{-2}
ENSRNOG00000004737	*Cd48*	2.28	5.56×10^{-3}
ENSRNOG00000012886	*Maff*	2.28	1.43×10^{-3}
ENSRNOG00000020470	*Slc25a24*	2.28	1.99×10^{-2}
ENSRNOG00000020325	*Calhm2*	2.28	3.23×10^{-2}
ENSRNOG00000011599	*Gldc*	2.27	1.20×10^{-2}
ENSRNOG00000017445	*Tubb2b*	2.27	2.02×10^{-2}
ENSRNOG00000010951	*Cmtm6*	2.27	7.74×10^{-3}
ENSRNOG00000012494	*Kctd14*	2.26	2.02×10^{-2}
ENSRNOG00000012840	*Sparc*	2.26	1.35×10^{-5}
ENSRNOG00000027839	*Ptk2b*	2.26	8.96×10^{-3}
ENSRNOG00000002244	*Pdgfra*	2.25	1.07×10^{-3}
ENSRNOG00000010794	*Dennd3*	2.25	1.11×10^{-2}
ENSRNOG00000023126	*Rxfp3*	2.25	4.14×10^{-2}
ENSRNOG00000042070	*Ticam2*	2.25	3.05×10^{-2}
ENSRNOG00000008196	*Parp12*	2.24	1.29×10^{-2}
ENSRNOG00000019176	*Kcne5*	2.24	3.99×10^{-2}
ENSRNOG00000023109	*Icoslg*	2.24	1.49×10^{-3}
ENSRNOG00000037562	*Zbtb42*	2.23	8.13×10^{-4}
ENSRNOG00000008190	*Pnpla7*	2.23	4.22×10^{-2}
ENSRNOG00000009243	*Oaf*	2.23	4.16×10^{-3}
ENSRNOG00000011150	*Arsb*	2.23	2.03×10^{-2}
ENSRNOG00000020882	*Shkbp1*	2.23	1.33×10^{-2}
ENSRNOG00000004673	*Rbm43*	2.23	4.92×10^{-2}
ENSRNOG00000056585	*Fscn1*	2.23	1.35×10^{-3}
ENSRNOG00000057826	*AABR07040412.1*	2.22	4.80×10^{-2}
ENSRNOG00000029195	*Uba7*	2.21	2.90×10^{-2}

ENSRNOG00000030467	*Ube2l6*	2.21	4.15×10^{-3}
ENSRNOG00000002480	*Gpr137b*	2.21	1.72×10^{-2}
ENSRNOG00000006956	*AABR07049085.1*	2.21	2.46×10^{-2}
ENSRNOG00000012074	*Ifngr1*	2.21	1.08×10^{-2}
ENSRNOG00000012660	*Postn*	2.20	1.17×10^{-2}
ENSRNOG00000013707	*Spata13*	2.20	8.56×10^{-3}
ENSRNOG00000013452	*Rcn1*	2.19	1.67×10^{-2}
ENSRNOG00000009980	*Plpp1*	2.19	1.49×10^{-2}
ENSRNOG00000014202	*Snx20*	2.19	3.97×10^{-2}
ENSRNOG00000019778	*Cavin1*	2.19	4.48×10^{-2}
ENSRNOG00000061928	*Myo1e*	2.18	1.67×10^{-2}
ENSRNOG00000059406	*Ier5*	2.18	2.06×10^{-4}
ENSRNOG00000051922	*AABR07059663.1*	2.18	2.74×10^{-2}
ENSRNOG00000031041	*Rps4y2*	2.18	1.68×10^{-2}
ENSRNOG00000011882	*Gab2*	2.18	1.95×10^{-3}
ENSRNOG00000002191	*LOC498368*	2.17	2.37×10^{-2}
ENSRNOG00000001235	*Gna12*	2.17	3.05×10^{-3}
ENSRNOG00000000940	*Flt1*	2.17	2.68×10^{-3}
ENSRNOG00000013190	*Rnaset2*	2.17	1.17×10^{-2}
ENSRNOG00000057927	*AABR07035796.1*	2.17	1.64×10^{-2}
ENSRNOG00000020573	*Efna1*	2.17	2.36×10^{-3}
ENSRNOG00000020918	*Ccnd1*	2.17	6.59×10^{-3}
ENSRNOG00000047450	*Lmo3*	2.17	3.27×10^{-2}
ENSRNOG00000042592	*Rgs10*	2.17	9.79×10^{-3}
ENSRNOG00000021220	*Cpxm1*	2.16	2.94×10^{-2}
ENSRNOG00000000579	*Marcks*	2.15	1.69×10^{-2}
ENSRNOG00000006623	*Cd302*	2.15	7.04×10^{-3}
ENSRNOG00000014278	*Zfp710*	2.14	8.67×10^{-3}
ENSRNOG00000025151	*Ildr2*	2.14	4.50×10^{-4}
ENSRNOG00000056524	*Abcd1*	2.14	3.63×10^{-2}
ENSRNOG00000048875	*Znrf4*	2.13	3.63×10^{-2}
ENSRNOG00000029614	*Robo1*	2.13	2.02×10^{-2}
ENSRNOG00000016516	*Mbp*	2.13	4.09×10^{-2}
ENSRNOG00000049052	*Sgk3*	2.13	2.02×10^{-2}
ENSRNOG00000008421	*Klhl5*	2.13	1.15×10^{-3}
ENSRNOG00000003330	*Acsf2*	2.12	8.90×10^{-3}
ENSRNOG00000027552	*Tapbpl*	2.12	6.57×10^{-3}
ENSRNOG00000002358	*Scpep1*	2.12	4.22×10^{-2}
ENSRNOG00000004160	*Prps2*	2.12	4.37×10^{-2}
ENSRNOG00000031168	*Arhgap15*	2.12	7.09×10^{-3}
ENSRNOG00000010897	*Nek6*	2.12	9.79×10^{-3}
ENSRNOG00000014893	*Wdr63*	2.11	3.83×10^{-2}
ENSRNOG00000005573	*Ntn4*	2.11	7.82×10^{-4}
ENSRNOG00000013069	*Sapcd2*	2.11	1.96×10^{-2}
ENSRNOG00000025115	*Exph5*	2.10	2.74×10^{-2}
ENSRNOG00000042411	*Rps6ka1*	2.10	2.13×10^{-3}
ENSRNOG00000003349	*LOC103693323*	2.10	4.99×10^{-2}
ENSRNOG00000020300	*Lsp1*	2.10	9.72×10^{-3}
ENSRNOG00000011562	*Slitrk2*	2.09	3.46×10^{-2}

ENSRNOG00000015746	*Kcng4*	2.09	1.69×10^{-2}
ENSRNOG00000011559	*Cnn3*	2.09	6.37×10^{-3}
ENSRNOG00000012611	*Lpxn*	2.09	1.77×10^{-2}
ENSRNOG00000008554	*Slc9a9*	2.09	2.09×10^{-2}
ENSRNOG00000047884	*AABR07001923.1*	2.09	2.16×10^{-2}
ENSRNOG00000007025	*Evc2*	2.09	2.64×10^{-2}
ENSRNOG00000017077	*Snx7*	2.08	1.22×10^{-4}
ENSRNOG00000005413	*Creb3l1*	2.08	3.51×10^{-2}
ENSRNOG00000018991	*Gsn*	2.08	3.30×10^{-2}
ENSRNOG00000015822	*Klf13*	2.08	4.22×10^{-2}
ENSRNOG00000048315	*Eif2ak2*	2.08	8.32×10^{-5}
ENSRNOG00000023352	*Fam78a*	2.08	4.67×10^{-2}
ENSRNOG00000019147	*Stom*	2.07	2.32×10^{-2}
ENSRNOG00000015496	*Tpm4*	2.06	2.43×10^{-3}
ENSRNOG00000021150	*Plcb3*	2.06	4.07×10^{-2}
ENSRNOG00000016592	*Gnai2*	2.06	8.50×10^{-3}
ENSRNOG00000015416	*Nabp1*	2.06	2.95×10^{-5}
ENSRNOG00000033608	*Cd276*	2.05	1.49×10^{-2}
ENSRNOG00000004307	*Tor3a*	2.05	2.68×10^{-2}
ENSRNOG00000018903	*Pik3r1*	2.05	2.32×10^{-3}
ENSRNOG00000048915	*Twf2*	2.05	2.73×10^{-2}
ENSRNOG00000016573	*Dgat2*	2.05	2.17×10^{-2}
ENSRNOG00000003235	*Mgat4b*	2.05	2.53×10^{-2}
ENSRNOG00000009323	*Fam214b*	2.05	2.22×10^{-8}
ENSRNOG00000062002	*Kcna3*	2.04	2.52×10^{-2}
ENSRNOG00000008706	*Tbx3*	2.04	2.69×10^{-2}
ENSRNOG00000016866	*Fhl2*	2.04	4.32×10^{-2}
ENSRNOG00000021087	*Lgi4*	2.04	9.07×10^{-5}
ENSRNOG00000027540	*Fam102b*	2.03	5.01×10^{-3}
ENSRNOG00000058057	*AABR07019403.1*	2.03	3.51×10^{-2}
ENSRNOG00000009884	*Lgals1*	2.03	4.09×10^{-2}
ENSRNOG00000026857	*Kif7*	2.03	3.34×10^{-2}
ENSRNOG00000024874	*Prtfdc1*	2.02	2.37×10^{-3}
ENSRNOG00000013017	*Arnt2*	2.02	1.88×10^{-4}
ENSRNOG00000059750	*AC141102.1*	2.02	3.07×10^{-2}
ENSRNOG00000002524	*Gpr37*	2.02	3.92×10^{-2}
ENSRNOG00000030983	*B3galt5*	2.02	3.94×10^{-2}
ENSRNOG00000058545	*Arhgap4*	2.01	3.24×10^{-2}
ENSRNOG00000013013	*Tle3*	2.01	1.28×10^{-2}
ENSRNOG00000005920	*AABR07054266.1*	2.01	3.50×10^{-2}
ENSRNOG00000052424	*RGD1306556*	2.01	2.89×10^{-3}
ENSRNOG00000004624	*Rnd3*	2.01	1.71×10^{-3}
ENSRNOG00000013045	*Setd7*	2.00	6.16×10^{-3}
ENSRNOG00000010813	*Tspan14*	2.00	6.79×10^{-3}
ENSRNOG00000015321	*Moxd1*	2.00	1.18×10^{-4}
ENSRNOG00000012630	*Rhoc*	2.00	2.03×10^{-3}
ENSRNOG00000016299	*Klf4*	2.00	4.97×10^{-2}
ENSRNOG00000034015	*Capn2*	2.00	5.46×10^{-3}
ENSRNOG00000048812	*Gpx1*	1.99	4.12×10^{-2}

ENSRNOG00000048870	*St6galnac4*	1.99	2.41×10^{-2}
ENSRNOG00000020871	*Ltbp4*	1.99	4.37×10^{-2}
ENSRNOG00000009263	*Ifi27*	1.98	7.45×10^{-4}
ENSRNOG00000012582	*Eif4ebp1*	1.98	1.07×10^{-3}
ENSRNOG00000010332	*Nipsnap3b*	1.98	4.37×10^{-2}
ENSRNOG00000000875	*Fhl1*	1.98	1.82×10^{-2}
ENSRNOG00000000777	*RT1-S3*	1.97	3.64×10^{-3}
ENSRNOG00000037814	*Cxcl17*	1.97	2.84×10^{-2}
ENSRNOG00000031163	*Nfkbiz*	1.97	2.07×10^{-2}
ENSRNOG00000000827	*Ier3*	1.97	2.77×10^{-2}
ENSRNOG00000008620	*Smad3*	1.97	2.92×10^{-2}
ENSRNOG00000027234	*Zfp367*	1.97	3.02×10^{-3}
ENSRNOG00000018517	*Trim21*	1.97	2.81×10^{-2}
ENSRNOG00000000142	*Plxdc2*	1.97	4.95×10^{-2}
ENSRNOG00000037835	*Catip*	1.96	4.24×10^{-2}
ENSRNOG00000039025	*AABR07051947.1*	1.96	2.76×10^{-2}
ENSRNOG00000013160	*Sash1*	1.96	2.75×10^{-2}
ENSRNOG00000036688	*Arhgdia*	1.96	2.84×10^{-3}
ENSRNOG00000010331	*Ctsb*	1.96	3.58×10^{-3}
ENSRNOG00000026136	*Tnfaip8*	1.96	2.75×10^{-2}
ENSRNOG00000022218	*Ifi44*	1.96	1.51×10^{-2}
ENSRNOG00000002969	*Itpkb*	1.95	1.60×10^{-2}
ENSRNOG00000010060	*Panx1*	1.95	2.41×10^{-2}
ENSRNOG00000004666	*Prr5l*	1.95	4.52×10^{-2}
ENSRNOG00000059015	*Triobp*	1.95	4.30×10^{-3}
ENSRNOG00000011313	*Sorcs1*	1.94	4.72×10^{-2}
ENSRNOG00000022636	*Alpk1*	1.94	2.75×10^{-2}
ENSRNOG00000042499	*Tmsb10*	1.94	8.05×10^{-3}
ENSRNOG00000019328	*Phgdh*	1.94	4.16×10^{-9}
ENSRNOG00000013798	*Fnbp1l*	1.93	1.68×10^{-4}
ENSRNOG00000007822	*Vgll4*	1.93	1.91×10^{-2}
ENSRNOG00000030537	*Slc26a11*	1.93	4.38×10^{-2}
ENSRNOG00000016244	*Mical2*	1.93	3.51×10^{-2}
ENSRNOG00000023226	*S100a10*	1.92	1.29×10^{-2}
ENSRNOG00000019604	*Pld2*	1.92	3.04×10^{-2}
ENSRNOG00000010233	*Cald1*	1.92	1.56×10^{-2}
ENSRNOG00000000164	*Lamp2*	1.91	1.84×10^{-2}
ENSRNOG00000038480	*Ppp1r36*	1.91	3.34×10^{-6}
ENSRNOG00000002278	*Tec*	1.91	9.19×10^{-4}
ENSRNOG00000002163	*Klf3*	1.91	9.91×10^{-3}
ENSRNOG00000002331	*Aldh3a1*	1.90	1.11×10^{-2}
ENSRNOG00000049471	*Steap3*	1.90	3.22×10^{-2}
ENSRNOG00000017496	*Cnp*	1.89	2.75×10^{-2}
ENSRNOG00000001242	*Gstt3*	1.89	2.41×10^{-2}
ENSRNOG00000016050	*Fgfr1*	1.89	2.33×10^{-2}
ENSRNOG00000009837	*Tmem131l*	1.89	2.51×10^{-2}
ENSRNOG00000019161	*Cpeb1*	1.88	1.11×10^{-2}
ENSRNOG00000026036	*Pdyn*	1.88	3.64×10^{-3}
ENSRNOG00000011521	*Filip1*	1.88	4.64×10^{-3}

ENSRNOG00000002963	*C1ql1*	1.88	1.99×10^{-3}
ENSRNOG00000017072	*Slc16a14*	1.88	3.45×10^{-2}
ENSRNOG00000003809	*Sat1*	1.88	5.46×10^{-3}
ENSRNOG00000015594	*Rftn2*	1.88	3.98×10^{-2}
ENSRNOG00000010984	*Anxa11*	1.88	4.51×10^{-2}
ENSRNOG00000052354	*Arhgef40*	1.87	3.67×10^{-2}
ENSRNOG00000019528	*Plekhg6*	1.87	3.54×10^{-2}
ENSRNOG00000017438	*AABR07007068.1*	1.87	1.31×10^{-2}
ENSRNOG00000025053	*Lrp1*	1.87	1.15×10^{-2}
ENSRNOG00000016334	*Ptbp3*	1.87	4.71×10^{-2}
ENSRNOG00000000521	*Cdkn1a*	1.87	1.58×10^{-3}
ENSRNOG00000015078	*Ifitm3*	1.86	1.97×10^{-2}
ENSRNOG00000031709	*Ppfibp1*	1.86	3.82×10^{-2}
ENSRNOG00000003453	*Lypd1*	1.86	4.70×10^{-3}
ENSRNOG00000001960	*Sult1d1*	1.86	4.16×10^{-3}
ENSRNOG00000020678	*Ifi35*	1.85	4.37×10^{-2}
ENSRNOG00000011861	*Aadat*	1.85	6.32×10^{-4}
ENSRNOG00000017933	*Ccdc3*	1.85	5.30×10^{-3}
ENSRNOG00000013886	*Fyb1*	1.84	1.97×10^{-2}
ENSRNOG00000001515	*Map3k20*	1.84	1.25×10^{-2}
ENSRNOG00000019977	*Ptprf*	1.84	4.39×10^{-2}
ENSRNOG00000001647	*Ets2*	1.84	1.54×10^{-3}
ENSRNOG00000004677	*Zeb2*	1.84	6.85×10^{-3}
ENSRNOG00000016265	*Acsl5*	1.84	1.12×10^{-2}
ENSRNOG00000016885	*Klf6*	1.83	1.13×10^{-4}
ENSRNOG00000014530	*Nav2*	1.83	1.54×10^{-2}
ENSRNOG00000056297	*Fmnl3*	1.83	1.57×10^{-2}
ENSRNOG00000053240	*Soga1*	1.82	4.93×10^{-2}
ENSRNOG00000024501	*Rgs3*	1.82	1.35×10^{-3}
ENSRNOG00000013589	*Cxcl12*	1.82	1.47×10^{-2}
ENSRNOG00000025764	*AC128848.1*	1.81	4.85×10^{-2}
ENSRNOG00000001169	*Slc37a1*	1.81	7.91×10^{-3}
ENSRNOG00000046005	*Scd2*	1.80	6.44×10^{-5}
ENSRNOG00000057620	*Slc6a8*	1.80	1.95×10^{-4}
ENSRNOG00000009982	*Pnp*	1.80	1.39×10^{-2}
ENSRNOG00000020936	*Nradd*	1.80	2.07×10^{-2}
ENSRNOG00000013973	*Lcn2*	1.80	3.32×10^{-2}
ENSRNOG00000046700	*Mettl27*	1.79	4.32×10^{-2}
ENSRNOG00000048922	*LOC100909664*	1.79	1.27×10^{-4}
ENSRNOG00000016254	*Sema4c*	1.79	1.20×10^{-2}
ENSRNOG00000048932	*Smagp*	1.79	1.54×10^{-2}
ENSRNOG00000015845	*Fam129b*	1.78	3.88×10^{-4}
ENSRNOG00000010964	*Akap13*	1.78	1.77×10^{-2}
ENSRNOG00000016541	*Enc1*	1.77	9.88×10^{-3}
ENSRNOG00000015401	*Mapk4*	1.77	3.31×10^{-3}
ENSRNOG00000000441	*Gpsm3*	1.77	1.37×10^{-2}
ENSRNOG00000003346	*Fancb*	1.77	4.43×10^{-3}
ENSRNOG00000020705	*Rnls*	1.77	2.57×10^{-2}
ENSRNOG00000023411	*Vsig10l*	1.76	4.49×10^{-4}

ENSRNOG00000000185	*Mpst*	1.76	1.64×10^{-2}
ENSRNOG00000015415	*Rhoq*	1.76	5.13×10^{-7}
ENSRNOG00000029242	*Plekho2*	1.76	1.61×10^{-2}
ENSRNOG00000001645	*Filip1l*	1.76	2.44×10^{-2}
ENSRNOG00000017610	*Nedd4l*	1.75	1.65×10^{-2}
ENSRNOG00000001295	*S100b*	1.75	1.21×10^{-2}
ENSRNOG00000032576	*Nlgn1*	1.75	4.43×10^{-2}
ENSRNOG00000012830	*Paqr8*	1.75	1.65×10^{-2}
ENSRNOG00000006227	*Ifih1*	1.74	1.65×10^{-2}
ENSRNOG00000021176	*Mtmr11*	1.74	1.13×10^{-2}
ENSRNOG00000055583	*Ttyh3*	1.73	4.04×10^{-2}
ENSRNOG00000025059	*Nxph4*	1.72	1.42×10^{-2}
ENSRNOG00000000936	*Stx2*	1.71	4.18×10^{-2}
ENSRNOG00000014142	*Ogfrl1*	1.71	2.53×10^{-2}
ENSRNOG00000002305	*Slc15a2*	1.71	4.36×10^{-2}
ENSRNOG00000014426	*Lox*	1.70	2.22×10^{-2}
ENSRNOG00000014079	*Stat1*	1.70	6.00×10^{-4}
ENSRNOG00000006388	*Pygl*	1.70	3.48×10^{-2}
ENSRNOG00000008482	*Rbms1*	1.70	4.52×10^{-2}
ENSRNOG00000023079	*Nras*	1.70	2.19×10^{-2}
ENSRNOG00000001385	*Plbd2*	1.69	1.67×10^{-2}
ENSRNOG00000020881	*Frmd8*	1.69	2.44×10^{-2}
ENSRNOG00000003538	*Adamts4*	1.68	3.56×10^{-2}
ENSRNOG00000007650	*Cd63*	1.68	1.63×10^{-2}
ENSRNOG00000021095	*Fxyd3*	1.68	3.78×10^{-2}
ENSRNOG00000015967	*Sh3bgrl3*	1.68	2.77×10^{-2}
ENSRNOG00000013783	*Efhd2*	1.68	2.32×10^{-2}
ENSRNOG00000027914	*Plscr3*	1.67	3.38×10^{-2}
ENSRNOG00000021403	*Rhob*	1.67	8.37×10^{-3}
ENSRNOG00000005708	*Mmp16*	1.67	4.06×10^{-2}
ENSRNOG00000040110	*Foxn2*	1.67	3.43×10^{-2}
ENSRNOG00000019742	*Stat3*	1.67	1.76×10^{-3}
ENSRNOG00000017311	*Me3*	1.67	1.31×10^{-2}
ENSRNOG00000040287	*Cyp1b1*	1.67	4.68×10^{-4}
ENSRNOG00000002553	*Smim14*	1.67	7.77×10^{-5}
ENSRNOG00000026128	*Cpne8*	1.66	2.44×10^{-2}
ENSRNOG00000052157	*Nav3*	1.66	2.81×10^{-2}
ENSRNOG00000026293	*Jun*	1.66	3.28×10^{-3}
ENSRNOG00000023116	*Agmo*	1.66	2.69×10^{-2}
ENSRNOG00000047307	*Cntfr*	1.65	6.20×10^{-3}
ENSRNOG00000020822	*Atp8b2*	1.64	4.07×10^{-3}
ENSRNOG00000024631	*Chadl*	1.64	3.03×10^{-2}
ENSRNOG00000054259	*Klf11*	1.64	1.38×10^{-2}
ENSRNOG00000012868	*Uaca*	1.64	1.63×10^{-2}
ENSRNOG00000039207	*Ccdc71l*	1.63	2.44×10^{-2}
ENSRNOG00000027722	*H1fx*	1.63	2.51×10^{-2}
ENSRNOG00000015177	*Sun2*	1.63	4.64×10^{-2}
ENSRNOG00000013228	*Scrg1*	1.63	1.62×10^{-2}
ENSRNOG00000006916	*Sardh*	1.63	4.37×10^{-2}

ENSRNOG00000007584	*Ehd4*	1.63	2.42×10^{-2}
ENSRNOG00000031824	*Slc44a2*	1.63	4.48×10^{-2}
ENSRNOG00000008626	*Manea*	1.62	1.49×10^{-3}
ENSRNOG00000013661	*Kif26a*	1.62	4.38×10^{-2}
ENSRNOG00000003273	*Spata20*	1.62	2.75×10^{-2}
ENSRNOG00000016357	*Casc4*	1.61	2.64×10^{-2}
ENSRNOG00000001409	*Gnb2*	1.61	1.12×10^{-2}
ENSRNOG00000026212	*Micall1*	1.61	1.85×10^{-6}
ENSRNOG00000011582	*Rab3d*	1.60	4.80×10^{-2}
ENSRNOG00000039415	*Fchsd1*	1.60	1.21×10^{-2}
ENSRNOG00000019496	*Stat5a*	1.60	4.46×10^{-2}
ENSRNOG00000018205	*Ttl*	1.60	1.75×10^{-2}
ENSRNOG00000007177	*Kdelc2*	1.60	3.91×10^{-2}
ENSRNOG00000045948	*Arl10*	1.59	1.35×10^{-2}
ENSRNOG00000003289	*Lap3*	1.58	4.03×10^{-2}
ENSRNOG00000010941	*Tifa*	1.58	3.56×10^{-2}
ENSRNOG00000005048	*Trhr*	1.58	3.51×10^{-2}
ENSRNOG00000007713	*Tmcc3*	1.58	4.52×10^{-2}
ENSRNOG00000017354	*Zyx*	1.58	3.13×10^{-2}
ENSRNOG00000023896	*Dusp6*	1.58	2.00×10^{-2}
ENSRNOG00000042477	*Clrn1*	1.58	7.03×10^{-3}
ENSRNOG00000028649	*Tox3*	1.57	2.84×10^{-2}
ENSRNOG00000048723	*Pros1*	1.57	4.09×10^{-2}
ENSRNOG00000001930	*Ccdc50*	1.57	1.82×10^{-2}
ENSRNOG00000047817	*Enox1*	1.57	2.38×10^{-2}
ENSRNOG00000049507	*Sept10*	1.56	1.08×10^{-2}
ENSRNOG00000017783	*Sfrp1*	1.56	1.63×10^{-2}
ENSRNOG00000016733	*Rab13*	1.56	2.21×10^{-2}
ENSRNOG00000026985	*Phldb1*	1.56	4.80×10^{-2}
ENSRNOG00000019859	*Pla2g15*	1.55	8.31×10^{-3}
ENSRNOG00000012561	*Arhgef10*	1.55	2.55×10^{-2}
ENSRNOG00000007345	*Amot*	1.55	2.03×10^{-3}
ENSRNOG00000031851	*Ndufa4l2*	1.55	8.93×10^{-3}
ENSRNOG00000011445	*Nkain1*	1.55	3.91×10^{-2}
ENSRNOG00000012053	*S100a16*	1.55	5.35×10^{-3}
ENSRNOG00000018279	*Sfxn1*	1.54	1.18×10^{-2}
ENSRNOG00000034254	*Actb*	1.54	2.51×10^{-2}
ENSRNOG00000017057	*Tbc1d22a*	1.53	1.09×10^{-2}
ENSRNOG00000010381	*Mknk1*	1.53	4.54×10^{-2}
ENSRNOG00000024595	*Cers6*	1.53	4.70×10^{-2}
ENSRNOG00000030055	*Vamp3*	1.53	2.60×10^{-2}
ENSRNOG00000011775	*Mfap3l*	1.52	9.82×10^{-3}
ENSRNOG00000010358	*Sh3rf1*	1.52	2.77×10^{-2}
ENSRNOG00000025040	*Gng10*	1.52	2.01×10^{-3}
ENSRNOG00000009076	*Ttpal*	1.51	4.07×10^{-2}
ENSRNOG00000003248	*Mpzl1*	1.51	1.05×10^{-2}
ENSRNOG00000013280	*Olfm4*	1.51	4.54×10^{-2}
ENSRNOG00000011203	*Farp1*	1.51	3.03×10^{-2}
ENSRNOG00000015948	*Slc1a5*	1.51	1.61×10^{-5}

ENSRNOG00000010219	*Ralgds*	1.51	4.58×10^{-2}
ENSRNOG00000014170	*Dbn1*	1.51	2.13×10^{-2}
ENSRNOG00000013035	*Rab33b*	1.50	2.61×10^{-2}
ENSRNOG00000017737	*Dgkz*	1.50	4.41×10^{-2}
ENSRNOG00000009687	*Ccdc120*	1.50	1.28×10^{-2}
ENSRNOG00000021117	*Rps6ka4*	1.50	2.44×10^{-2}
ENSRNOG00000012821	*Cnmd*	1.50	4.85×10^{-2}
ENSRNOG00000001277	*Mafk*	1.50	1.25×10^{-4}
ENSRNOG00000015518	*Rbp4*	1.50	4.80×10^{-3}
ENSRNOG00000014276	*Plce1*	1.50	1.16×10^{-2}
ENSRNOG00000017441	*Tpm3*	1.50	1.50×10^{-2}
ENSRNOG00000007654	*Lrig3*	1.49	4.43×10^{-2}
ENSRNOG00000009983	*Lrrc42*	1.49	3.02×10^{-2}
ENSRNOG00000000219	*Ano10*	1.48	2.33×10^{-2}
ENSRNOG00000012404	*Thrsp*	1.48	1.67×10^{-2}
ENSRNOG00000002280	*Sh3bgrl*	1.48	5.61×10^{-4}
ENSRNOG00000011424	*Cldn23*	1.48	4.95×10^{-2}
ENSRNOG00000010635	*Igfbp4*	1.48	1.89×10^{-3}
ENSRNOG00000042576	*Tcp11l1*	1.48	3.51×10^{-2}
ENSRNOG00000018536	*Pck2*	1.47	3.53×10^{-2}
ENSRNOG00000001431	*Rasa4*	1.47	4.51×10^{-2}
ENSRNOG00000046905	*Sgce*	1.47	4.30×10^{-2}
ENSRNOG00000010489	*Samd4a*	1.46	9.41×10^{-3}
ENSRNOG00000007136	*Anxa7*	1.46	4.07×10^{-2}
ENSRNOG00000016670	*AABR07028769.1*	1.46	4.94×10^{-2}
ENSRNOG00000012473	*Cflar*	1.45	2.79×10^{-2}
ENSRNOG00000047931	*Tmsb4x*	1.45	3.84×10^{-3}
ENSRNOG00000056216	*Casp7*	1.45	3.48×10^{-2}
ENSRNOG00000054080	*Cgnl1*	1.45	1.09×10^{-2}
ENSRNOG00000010058	*Spry2*	1.44	2.32×10^{-2}
ENSRNOG00000057713	*Cav2*	1.44	6.94×10^{-3}
ENSRNOG00000006813	*Sumf1*	1.43	6.40×10^{-3}
ENSRNOG00000018390	*Pld3*	1.43	1.67×10^{-2}
ENSRNOG00000058003	*Spon1*	1.43	2.26×10^{-2}
ENSRNOG00000015072	*Ptgr1*	1.43	1.15×10^{-2}
ENSRNOG00000007152	*Bhlhe40*	1.43	6.19×10^{-3}
ENSRNOG00000018262	*Ampd3*	1.42	4.09×10^{-2}
ENSRNOG00000019356	*Lrfn4*	1.42	4.87×10^{-2}
ENSRNOG00000006526	*Sema3c*	1.42	2.97×10^{-2}
ENSRNOG00000002896	*Prdx6*	1.41	3.07×10^{-3}
ENSRNOG00000016043	*Aqp4*	1.41	4.37×10^{-2}
ENSRNOG00000013911	*Nagk*	1.41	4.14×10^{-2}
ENSRNOG00000000247	*Mfsd11*	1.41	3.12×10^{-2}
ENSRNOG00000047319	*Caprin2*	1.40	2.23×10^{-2}
ENSRNOG00000042838	*Junb*	1.39	4.34×10^{-3}
ENSRNOG00000011798	*Mapre1*	1.39	2.75×10^{-2}
ENSRNOG00000001057	*Ctxn1*	1.39	8.51×10^{-3}
ENSRNOG00000015054	*Rcbtb2*	1.39	4.53×10^{-2}
ENSRNOG00000046207	*Cbx4*	1.39	1.11×10^{-2}

ENSRNOG00000056243	*ST7*	1.38	2.32×10^{-2}
ENSRNOG00000018765	*Pold4*	1.38	1.21×10^{-2}
ENSRNOG00000019692	*Metrn*	1.37	5.76×10^{-5}
ENSRNOG00000016671	*Dtna*	1.36	3.43×10^{-2}
ENSRNOG00000014013	*Map4k4*	1.36	4.18×10^{-2}
ENSRNOG00000022325	*Smc2*	1.35	2.79×10^{-2}
ENSRNOG00000007307	*Syde1*	1.34	3.50×10^{-2}
ENSRNOG00000000123	*Rnf19b*	1.34	6.36×10^{-3}
ENSRNOG00000045779	*AABR07039210.2*	1.34	4.46×10^{-2}
ENSRNOG00000003891	*AC118957.1*	1.31	3.99×10^{-2}
ENSRNOG00000052232	*Cyb561d1*	1.31	2.09×10^{-2}
ENSRNOG00000010574	*Ptpn1*	1.30	2.18×10^{-2}
ENSRNOG00000027622	*Slc35e3*	1.30	1.43×10^{-2}
ENSRNOG00000005615	*Gadd45a*	1.29	9.36×10^{-3}
ENSRNOG00000001964	*Cd47*	1.29	4.52×10^{-2}
ENSRNOG00000011501	*Atp1b3*	1.29	3.38×10^{-2}
ENSRNOG00000002529	*Rap2c*	1.29	2.51×10^{-2}
ENSRNOG00000001415	*Ap1s1*	1.29	1.82×10^{-2}
ENSRNOG00000003687	*Rgs2*	1.29	1.54×10^{-2}
ENSRNOG00000025970	*AABR07068127.1*	1.28	5.54×10^{-3}
ENSRNOG00000015544	*LOC100909441*	1.28	4.28×10^{-2}
ENSRNOG00000012827	*Mlf1*	1.27	3.30×10^{-2}
ENSRNOG00000013971	*Psat1*	1.27	1.31×10^{-2}
ENSRNOG00000025695	*Tns3*	1.27	4.02×10^{-2}
ENSRNOG00000019568	*Jund*	1.27	2.84×10^{-2}
ENSRNOG00000008298	*Dock7*	1.27	3.97×10^{-2}
ENSRNOG00000001174	*Pde9a*	1.26	2.73×10^{-2}
ENSRNOG00000005865	*Itprid2*	1.26	6.17×10^{-3}
ENSRNOG00000012333	*Kbtbd11*	1.26	3.30×10^{-2}
ENSRNOG00000004147	*Abca8a*	1.23	4.58×10^{-3}
ENSRNOG00000054560	*Sh2d3c*	1.23	3.41×10^{-2}
ENSRNOG00000001785	*Etv5*	1.22	1.24×10^{-2}
ENSRNOG00000060087	*Adra1b*	1.22	1.46×10^{-2}
ENSRNOG00000016791	*Chka*	1.22	2.30×10^{-2}
ENSRNOG00000004003	*Dusp10*	1.22	4.35×10^{-2}
ENSRNOG00000030771	*Dgkb*	1.21	3.91×10^{-2}
ENSRNOG00000037871	*Sfxn5*	1.21	6.68×10^{-4}
ENSRNOG00000011561	*Nln*	1.20	7.14×10^{-3}
ENSRNOG00000000186	*Tst*	1.20	2.71×10^{-2}
ENSRNOG00000015473	*Phactr2*	1.20	2.81×10^{-2}
ENSRNOG00000050828	*Vkorc1*	1.20	1.74×10^{-2}
ENSRNOG00000060753	*Esyt1*	1.19	2.07×10^{-2}
ENSRNOG00000014479	*Cttnbp2nl*	1.19	2.61×10^{-2}
ENSRNOG00000020376	*Stn1*	1.17	4.48×10^{-2}
ENSRNOG00000009330	*Slc17a5*	1.17	4.15×10^{-2}
ENSRNOG00000010731	*Gpm6a*	1.17	1.96×10^{-2}
ENSRNOG00000019698	*Ssbp4*	1.16	1.42×10^{-2}
ENSRNOG00000025602	*Cdk4*	1.15	4.90×10^{-2}
ENSRNOG00000000697	*Coro1c*	1.15	1.12×10^{-2}

ENSRNOG00000051204	*Dop1b*	1.14	3.95×10^{-2}
ENSRNOG00000022507	*Twf1*	1.13	3.86×10^{-3}
ENSRNOG00000025372	*Glce*	1.12	4.71×10^{-2}
ENSRNOG00000011989	*Vat1l*	1.10	4.10×10^{-2}
ENSRNOG00000009636	*Scrn1*	1.10	3.76×10^{-2}
ENSRNOG00000003917	*Uck2*	1.09	2.52×10^{-2}
ENSRNOG00000002182	*Sept11*	1.07	3.93×10^{-2}
ENSRNOG00000009683	*Sdcbp*	1.07	1.31×10^{-2}
ENSRNOG00000045605	*Uxs1*	1.06	4.76×10^{-2}
ENSRNOG00000018494	*Ppp1r3c*	1.05	9.43×10^{-3}
ENSRNOG00000017132	*Snx30*	1.04	3.31×10^{-2}
ENSRNOG00000014293	*Nkd1*	1.04	3.91×10^{-2}
ENSRNOG00000049983	*Shd*	1.04	4.69×10^{-3}
ENSRNOG00000048088	*Mest*	1.03	2.97×10^{-2}
ENSRNOG00000047211	*Fzd3*	1.02	2.00×10^{-2}
ENSRNOG00000028746	*Gsto1*	1.02	3.11×10^{-2}

P_{corr}: corrected *P*-value

Supplementary table 3: Downregulated differentially expressed genes in rat retina with optic nerve injury.

Gene identity	Gene name	$\log_2$ Fold change	P_{corr}
ENSRNOG00000006491	*Foxi3*	-6.93	2.29×10^{-3}
ENSRNOG00000045686	*Nfs1*	-6.34	1.93×10^{-5}
ENSRNOG00000020025	*LOC108348052*	-5.37	4.52×10^{-2}
ENSRNOG00000003737	*Best2*	-5.31	3.48×10^{-3}
ENSRNOG00000061918	*AABR07069008.3*	-5.09	3.91×10^{-2}
ENSRNOG00000055862	*AABR07012762.1*	-4.94	2.71×10^{-2}
ENSRNOG00000046192	*LOC103690007*	-4.71	3.65×10^{-3}
ENSRNOG00000050441	*AABR07028989.1*	-4.61	8.29×10^{-3}
ENSRNOG00000030463	*Faim*	-4.39	4.26×10^{-2}
ENSRNOG00000009047	*Sln*	-4.02	4.28×10^{-2}
ENSRNOG00000001756	*LOC100911374*	-3.97	1.96×10^{-2}
ENSRNOG00000048561	*Hprt1*	-3.96	5.59×10^{-3}
ENSRNOG00000011184	*Slc13a4*	-3.85	8.21×10^{-3}
ENSRNOG00000054314	*Kcng1*	-3.83	1.29×10^{-2}
ENSRNOG00000050450	*Kcnip2*	-3.81	1.49×10^{-2}
ENSRNOG00000055179	*Tsen34l1*	-3.47	9.37×10^{-3}
ENSRNOG00000012720	*Irx4*	-3.25	3.91×10^{-2}
ENSRNOG00000057369	*AABR07027240.1*	-3.21	3.77×10^{-3}
ENSRNOG00000059883	*LOC100912578*	-3.16	4.58×10^{-9}
ENSRNOG00000030530	*Gzmm*	-3.10	1.24×10^{-2}
ENSRNOG00000015904	*Wfdc1*	-3.08	9.97×10^{-3}
ENSRNOG00000015896	*Rbpms2*	-2.94	1.04×10^{-3}
ENSRNOG00000050767	*Nrn1*	-2.75	8.48×10^{-3}
ENSRNOG00000004089	*Enpp2*	-2.69	9.66×10^{-3}
ENSRNOG00000054495	*Cldn2*	-2.64	2.19×10^{-4}
ENSRNOG00000009437	*LOC100912481*	-2.53	4.63×10^{-2}
ENSRNOG00000046973	*Tmco3*	-2.50	2.07×10^{-2}
ENSRNOG00000046602	*AABR07006030.1*	-2.49	3.51×10^{-2}
ENSRNOG00000049397	*LOC103689941*	-2.46	2.79×10^{-2}
ENSRNOG00000047516	*LOC100910771*	-2.43	3.01×10^{-2}
ENSRNOG00000058692	*Slc26a4*	-2.40	9.18×10^{-3}
ENSRNOG00000012876	*Slc6a13*	-2.40	8.48×10^{-4}
ENSRNOG00000008716	*Nefh*	-2.33	1.01×10^{-2}
ENSRNOG00000030069	*AABR07071258.1*	-2.29	4.80×10^{-2}
ENSRNOG00000055506	*1700020D05Rik*	-2.27	9.64×10^{-3}
ENSRNOG00000048973	*Ccnj*	-2.19	1.12×10^{-2}
ENSRNOG00000051688	*Syt15*	-2.11	3.26×10^{-2}
ENSRNOG00000053355	*RF00100*	-2.00	2.49×10^{-2}
ENSRNOG00000007044	*L3mbtl1*	-1.91	2.82×10^{-3}
ENSRNOG00000021098	*Rasgrp2*	-1.86	4.15×10^{-2}
ENSRNOG00000006025	*Lamb3*	-1.85	8.89×10^{-4}
ENSRNOG00000016983	*Myh7*	-1.78	1.11×10^{-3}
ENSRNOG00000042321	*AABR07052588.1*	-1.77	3.28×10^{-3}
ENSRNOG00000051438	*Tex28*	-1.73	1.74×10^{-7}
ENSRNOG00000051529	*Opn1mw*	-1.65	3.73×10^{-5}

ENSRNOG00000009979	*Sebox*	-1.65	3.22×10^{-2}
ENSRNOG00000048264	*Hist1h1d*	-1.64	4.79×10^{-3}
ENSRNOG00000031785	*Krt76*	-1.63	5.22×10^{-3}
ENSRNOG00000007078	*Ccn4*	-1.61	1.94×10^{-2}
ENSRNOG00000030434	*Rs1*	-1.57	1.76×10^{-3}
ENSRNOG00000057941	*AABR07036331.2*	-1.57	4.48×10^{-2}
ENSRNOG00000052051	*Slc24a1*	-1.53	1.82×10^{-5}
ENSRNOG00000059237	*AABR07068851.1*	-1.48	2.61×10^{-6}
ENSRNOG00000017589	*Gnat1*	-1.48	1.25 x 10-3
ENSRNOG00000005957	*Slc4a7*	-1.47	1.62×10^{-6}
ENSRNOG00000001194	*Rrp1b*	-1.42	1.09×10^{-3}
ENSRNOG00000011648	*Aqp1*	-1.42	4.46×10^{-5}
ENSRNOG00000021493	*Prdm9*	-1.41	4.36×10^{-2}
ENSRNOG00000056553	*Rdh12*	-1.41	3.03×10^{-4}
ENSRNOG00000038752	*AABR07009538.1*	-1.41	3.66×10^{-2}
ENSRNOG00000061572	*Smarcd1*	-1.39	2.08×10^{-3}
ENSRNOG00000017941	*Optn*	-1.39	2.40×10^{-4}
ENSRNOG00000004473	*Ppargc1a*	-1.39	3.11×10^{-3}
ENSRNOG00000037801	*AABR07036374.1*	-1.36	1.23×10^{-2}
ENSRNOG00000051911	*Rbp3*	-1.35	7.59×10^{-4}
ENSRNOG00000006116	*Hk2*	-1.34	2.37×10^{-3}
ENSRNOG00000002904	*Arr3*	-1.34	1.28×10^{-2}
ENSRNOG00000021105	*Gabpb2*	-1.33	1.81×10^{-3}
ENSRNOG00000000716	*Htr1f*	-1.33	1.70×10^{-2}
ENSRNOG00000010486	*Mpp4*	-1.32	9.78×10^{-6}
ENSRNOG00000011332	*Clspn*	-1.31	2.54×10^{-3}
ENSRNOG00000033262	*Reep6*	-1.30	3.57×10^{-2}
ENSRNOG00000007561	*Glb1l2*	-1.30	4.99×10^{-4}
ENSRNOG00000019086	*Gucy2f*	-1.30	4.12×10^{-4}
ENSRNOG00000003064	*Bst1*	-1.29	8.42×10^{-4}
ENSRNOG00000012479	*Impg1*	-1.28	1.63×10^{-2}
ENSRNOG00000010888	*Ankrd33b*	-1.28	9.79×10^{-3}
ENSRNOG00000018430	*Grk1*	-1.28	4.25×10^{-3}
ENSRNOG00000003800	*Rgs9*	-1.28	1.89×10^{-3}
ENSRNOG00000025757	*Myh6*	-1.27	9.04×10^{-3}
ENSRNOG00000016638	*Gnb1*	-1.27	1.22×10^{-3}
ENSRNOG00000013515	*Ptpru*	-1.26	2.15×10^{-3}
ENSRNOG00000042277	*AABR07016578.1*	-1.26	2.23×10^{-2}
ENSRNOG00000007687	*Sema7a*	-1.26	2.88×10^{-4}
ENSRNOG00000017260	*Cdr2*	-1.26	1.23×10^{-2}
ENSRNOG00000008510	*Abtb2*	-1.26	4.04×10^{-2}
ENSRNOG00000016827	*Slc38a3*	-1.25	1.90×10^{-3}
ENSRNOG00000004162	*Pfkfb2*	-1.25	2.01×10^{-3}
ENSRNOG00000022331	*Ccdc78*	-1.24	3.86×10^{-3}
ENSRNOG00000011144	*Rho*	-1.23	1.13×10^{-2}
ENSRNOG00000012892	*Abca4*	-1.23	7.20×10^{-4}
ENSRNOG00000017816	*Pde6a*	-1.23	2.08×10^{-3}
ENSRNOG00000000065	*Pde6b*	-1.22	6.45×10^{-3}
ENSRNOG00000031773	*Cngb1*	-1.22	8.96×10^{-3}

ENSRNOG00000013330	*Cdhr1*	-1.22	2.80×10^{-3}
ENSRNOG00000007304	*Herc3*	-1.22	2.28×10^{-3}
ENSRNOG00000006860	*Itk*	-1.22	4.23×10^{-2}
ENSRNOG00000017930	*Lpcat1*	-1.22	4.69×10^{-3}
ENSRNOG00000025979	*Gramd2a*	-1.21	4.60×10^{-2}
ENSRNOG00000004158	*Hdac9*	-1.21	4.52×10^{-2}
ENSRNOG00000047517	*Oxld1*	-1.21	2.81×10^{-2}
ENSRNOG00000023561	*Ano2*	-1.20	4.54×10^{-3}
ENSRNOG00000019914	*Fam57b*	-1.20	2.28×10^{-3}
ENSRNOG00000031671	*Rasgef1a*	-1.20	2.67×10^{-3}
ENSRNOG00000019752	*Slc29a1*	-1.20	3.27×10^{-4}
ENSRNOG00000007637	*Acer2*	-1.20	1.16×10^{-2}
ENSRNOG00000026171	*Bbs4*	-1.19	2.01×10^{-3}
ENSRNOG00000061348	*Fam53b*	-1.19	4.43×10^{-3}
ENSRNOG00000056434	*RF01233*	-1.19	1.61×10^{-2}
ENSRNOG00000047873	*Sec22c*	-1.19	7.62×10^{-3}
ENSRNOG00000054355	*AC128212.1*	-1.18	1.20×10^{-2}
ENSRNOG00000029321	*Ccser1*	-1.18	3.67×10^{-3}
ENSRNOG00000053979	*Egf*	-1.18	1.09×10^{-2}
ENSRNOG00000007764	*Frmd4b*	-1.18	2.16×10^{-2}
ENSRNOG00000006575	*Ccdc96*	-1.17	2.01×10^{-3}
ENSRNOG00000018378	*Cacnb2*	-1.17	7.98×10^{-3}
ENSRNOG00000003738	*Ush2a*	-1.17	5.36×10^{-4}
ENSRNOG00000016353	*Nim1k*	-1.16	6.36×10^{-3}
ENSRNOG00000005271	*Rapgef5*	-1.16	2.67×10^{-3}
ENSRNOG00000030232	*Hacd3*	-1.16	8.15×10^{-5}
ENSRNOG00000007931	*Gucy2d*	-1.16	1.51×10^{-2}
ENSRNOG00000016505	*Fam169a*	-1.16	2.26×10^{-3}
ENSRNOG00000011809	*AABR07072449.1*	-1.15	1.21×10^{-3}
ENSRNOG00000031760	*Fmn1*	-1.15	8.02×10^{-3}
ENSRNOG00000049517	*Tnfaip3*	-1.15	1.00×10^{-2}
ENSRNOG00000022044	*Cabp4*	-1.15	2.67×10^{-2}
ENSRNOG00000018373	*Tln2*	-1.15	6.11×10^{-3}
ENSRNOG00000026226	*Hook1*	-1.15	2.51×10^{-2}
ENSRNOG00000054770	*Rdh11*	-1.14	3.71×10^{-2}
ENSRNOG00000050690	*Nr2e3*	-1.14	1.61×10^{-2}
ENSRNOG00000019873	*Eml3*	-1.14	8.41×10^{-3}
ENSRNOG00000021628	*Wdr89*	-1.14	3.18×10^{-3}
ENSRNOG00000004402	*Lpgat1*	-1.14	1.27×10^{-4}
ENSRNOG00000004778	*Cnga1*	-1.13	4.68×10^{-3}
ENSRNOG00000013057	*Prc1*	-1.13	3.61×10^{-2}
ENSRNOG00000016356	*Got1*	-1.13	5.60×10^{-3}
ENSRNOG00000009773	*Elovl4*	-1.12	1.73×10^{-2}
ENSRNOG00000019645	*Osbp2*	-1.12	7.20×10^{-4}
ENSRNOG00000012343	*Pdp2*	-1.12	2.31×10^{-3}
ENSRNOG00000048847	*Wdr17*	-1.12	3.39×10^{-2}
ENSRNOG00000019005	*Pde8a*	-1.12	3.97×10^{-2}
ENSRNOG00000008209	*St3gal1*	-1.12	4.47×10^{-3}
ENSRNOG00000011644	*Slc1a7*	-1.11	1.31×10^{-2}

ENSRNOG00000013583	*Tbc1d8*	-1.11	2.89×10^{-3}
ENSRNOG00000001205	*Agpat3*	-1.11	2.36×10^{-3}
ENSRNOG00000003633	*Rcvrn*	-1.10	5.02×10^{-3}
ENSRNOG00000017405	*Raly*	-1.10	1.42×10^{-2}
ENSRNOG00000008471	*Kif21b*	-1.10	7.97×10^{-4}
ENSRNOG00000015816	*Bbs7*	-1.09	1.47×10^{-2}
ENSRNOG00000006967	*Xiap*	-1.09	1.46×10^{-2}
ENSRNOG00000008031	*Cacna2d4*	-1.09	3.44×10^{-2}
ENSRNOG00000008323	*Pitpnm3*	-1.09	1.49×10^{-2}
ENSRNOG00000021101	*Sema6c*	-1.09	2.95×10^{-2}
ENSRNOG00000020770	*Arl4d*	-1.08	4.46×10^{-2}
ENSRNOG00000008609	*Capn3*	-1.08	4.20×10^{-2}
ENSRNOG00000009019	*Slc6a6*	-1.08	1.59×10^{-2}
ENSRNOG00000019858	*Rom1*	-1.08	7.98×10^{-3}
ENSRNOG00000043387	*Cpe*	-1.08	3.31×10^{-2}
ENSRNOG00000000177	*Plpp2*	-1.08	1.54×10^{-2}
ENSRNOG00000050277	*Uckl1*	-1.07	4.89×10^{-3}
ENSRNOG00000021056	*Kcnj14*	-1.07	2.46×10^{-2}
ENSRNOG00000010280	*Pde8b*	-1.07	4.92×10^{-3}
ENSRNOG00000016011	*Plekhg1*	-1.06	4.94×10^{-2}
ENSRNOG00000016998	*Atxn1*	-1.06	3.23×10^{-3}
ENSRNOG00000021359	*Mtfr1*	-1.06	2.81×10^{-2}
ENSRNOG00000001151	*Sirt4*	-1.06	1.42×10^{-2}
ENSRNOG00000003033	*Plcd3*	-1.06	2.26×10^{-2}
ENSRNOG00000012495	*Podxl*	-1.05	5.30×10^{-3}
ENSRNOG00000055779	*Tdrd7*	-1.05	2.81×10^{-2}
ENSRNOG00000022166	*Ammecr1*	-1.05	1.38×10^{-2}
ENSRNOG00000036813	*AABR07054419.1*	-1.05	1.19×10^{-2}
ENSRNOG00000006689	*Chd7*	-1.05	2.66×10^{-3}
ENSRNOG00000010348	*Cacna1f*	-1.05	2.53×10^{-3}
ENSRNOG00000016635	*LOC361646*	-1.05	6.68×10^{-3}
ENSRNOG00000011160	*AC126641.1*	-1.04	2.29×10^{-3}
ENSRNOG00000038200	*Rttn*	-1.04	1.57×10^{-2}
ENSRNOG00000022268	*Pnpla3*	-1.04	3.83×10^{-2}
ENSRNOG00000024798	*Bora*	-1.04	1.75×10^{-2}
ENSRNOG00000015718	*RGD1307461*	-1.04	1.62×10^{-3}
ENSRNOG00000007172	*Map4k3*	-1.03	4.36×10^{-2}
ENSRNOG00000029061	*Hirip3*	-1.03	4.65×10^{-2}
ENSRNOG00000061097	*Lrit3*	-1.03	2.18×10^{-2}
ENSRNOG00000005096	*Bzw2*	-1.03	1.68×10^{-2}
ENSRNOG00000007323	*Ric8b*	-1.03	1.35×10^{-2}
ENSRNOG00000024364	*Enkd1*	-1.03	3.71×10^{-2}
ENSRNOG00000053430	*Slco4a1*	-1.03	3.11×10^{-2}
ENSRNOG00000047799	*Gnb5*	-1.03	2.37×10^{-2}
ENSRNOG00000025527	*Mtcl1*	-1.02	5.03×10^{-3}
ENSRNOG00000017404	*Pcmtd2*	-1.02	1.42×10^{-2}
ENSRNOG00000016398	*Clstn1*	-1.02	5.35×10^{-3}
ENSRNOG00000018526	*Dlg4*	-1.02	7.98×10^{-3}
ENSRNOG00000016593	*Pde6c*	-1.01	1.46×10^{-2}

ENSRNOG00000023238	*Dgkd*	-1.01	1.42×10^{-2}
ENSRNOG00000008961	*Mapre3*	-1.01	1.67×10^{-2}
ENSRNOG00000020263	*Atp1a3*	-1.01	3.87×10^{-2}
ENSRNOG00000024085	*Tmem237*	-1.01	2.51×10^{-2}
ENSRNOG00000029826	*Cep164*	-1.01	1.60×10^{-2}
ENSRNOG00000014573	*Ckmt1*	-1.00	1.22×10^{-2}
ENSRNOG00000000323	*Prdm1*	-1.00	4.15×10^{-2}
ENSRNOG00000001347	*Adam1a*	-1.00	3.24×10^{-2}

P_{corr}: corrected *P*-value

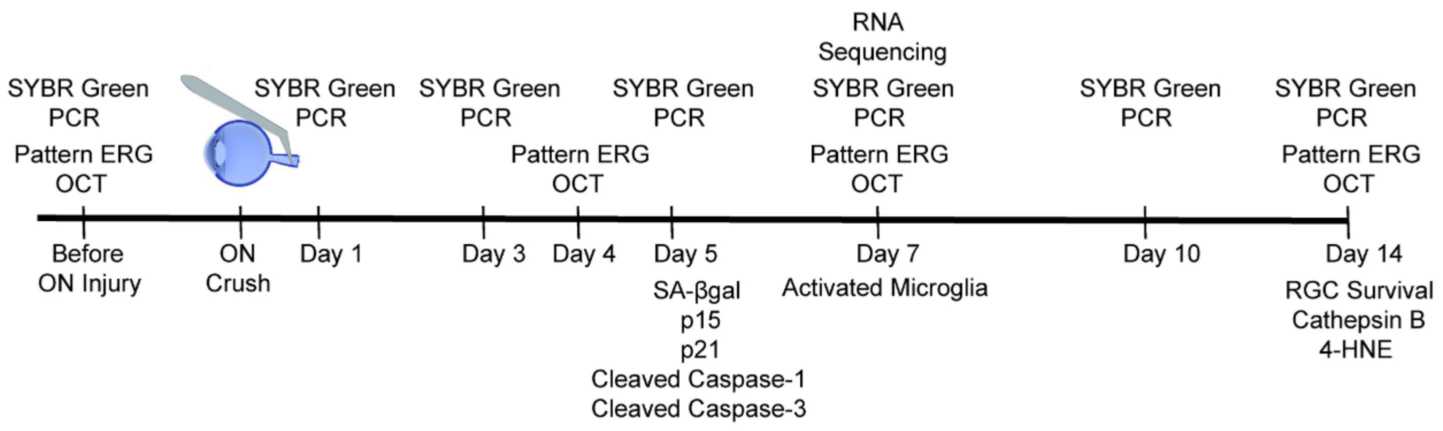

Supplementary figure 1: Schematic diagram of optic nerve crush injury, treatments, and experimental assessments.

The rats/mice were anesthetized, and optic nerve (ON) injury was induced by crushing the ON at 1 – 1.5 mm behind the eyeball for 5 sec without damaging the ophthalmic artery. RNA sequencing analysis was performed using the rat retina at Day 7 after ON injury, while the target gene validated by SYBR green PCR using the mouse retinas before the ON injury and at Day 1, 3, 5, 7, 10, and 14 after ON injury. The mice were intragastrically fed with LY2109761, dasatinib, and quercetin every day for 14 days after ON crush injury. Retinal ganglion cell (RGC) survival and the expression of cathepsin B and 4-hydroxynonenal (4-HNE) on mouse retinas were evaluated at Day 14 after ON injury. The retinal thickness and function of RGC were evaluated by optical coherence tomography (OCT) and pattern electroretinography (ERG) respectively in mice before the ON injury and at Day 4, 7, and 14 after ON injury. Microglial activation was evaluated on mouse retinas at Day 7 after ON injury. Senescence-associated β-galactosidase (SA-βgal) activity and the expression of $p15^{Ink4b}$, $p21^{Cip1}$, cleaved caspase-3, and cleaved caspase-1 protein was evaluated on mouse retinas at Day 5 after ON injury.
